# FRAP to Characterize Molecular Diffusion and Interaction in Various Membrane Environments

**DOI:** 10.1371/journal.pone.0158457

**Published:** 2016-07-07

**Authors:** Frédéric Pincet, Vladimir Adrien, Rong Yang, Jérôme Delacotte, James E. Rothman, Wladimir Urbach, David Tareste

**Affiliations:** 1 Laboratoire de Physique Statistique, Ecole Normale Supérieure, CNRS UMR 8550, Université Pierre et Marie Curie, Sorbonne Universités, Paris, France; 2 Department of Cell Biology, School of Medicine, Yale University, New Haven, CT, United States of America; 3 Laboratoire de Cristallographie et RMN Biologiques, CNRS UMR 8015, Université Paris Descartes, Sorbonne Paris Cité, Paris, France; 4 Department of Physiology and Cellular Biophysics, Columbia University, New York, United States of America; 5 UFR Biomédicale, Université Paris Descartes, Sorbonne Paris Cité, Paris, France; 6 Membrane Traffic in Health & Disease, INSERM ERL U950, Université Paris Diderot, Sorbonne Paris Cité, Paris, France; 7 Institut Jacques Monod, CNRS UMR 7592, Université Paris Diderot, Sorbonne Paris Cité, Paris, France; Oregon State University, UNITED STATES

## Abstract

Fluorescence recovery after photobleaching (FRAP) is a standard method used to study the dynamics of lipids and proteins in artificial and cellular membrane systems. The advent of confocal microscopy two decades ago has made quantitative FRAP easily available to most laboratories. Usually, a single bleaching pattern/area is used and the corresponding recovery time is assumed to directly provide a diffusion coefficient, although this is only true in the case of unrestricted Brownian motion. Here, we propose some general guidelines to perform FRAP experiments under a confocal microscope with different bleaching patterns and area, allowing the experimentalist to establish whether the molecules undergo Brownian motion (free diffusion) or whether they have restricted or directed movements. Using *in silico* simulations of FRAP measurements, we further indicate the data acquisition criteria that have to be verified in order to obtain accurate values for the diffusion coefficient and to be able to distinguish between different diffusive species. Using this approach, we compare the behavior of lipids in three different membrane platforms (supported lipid bilayers, giant liposomes and sponge phases), and we demonstrate that FRAP measurements are consistent with results obtained using other techniques such as Fluorescence Correlation Spectroscopy (FCS) or Single Particle Tracking (SPT). Finally, we apply this method to show that the presence of the synaptic protein Munc18-1 inhibits the interaction between the synaptic vesicle SNARE protein, VAMP2, and its partner from the plasma membrane, Syn1A.

## Introduction

**Fig 1:**
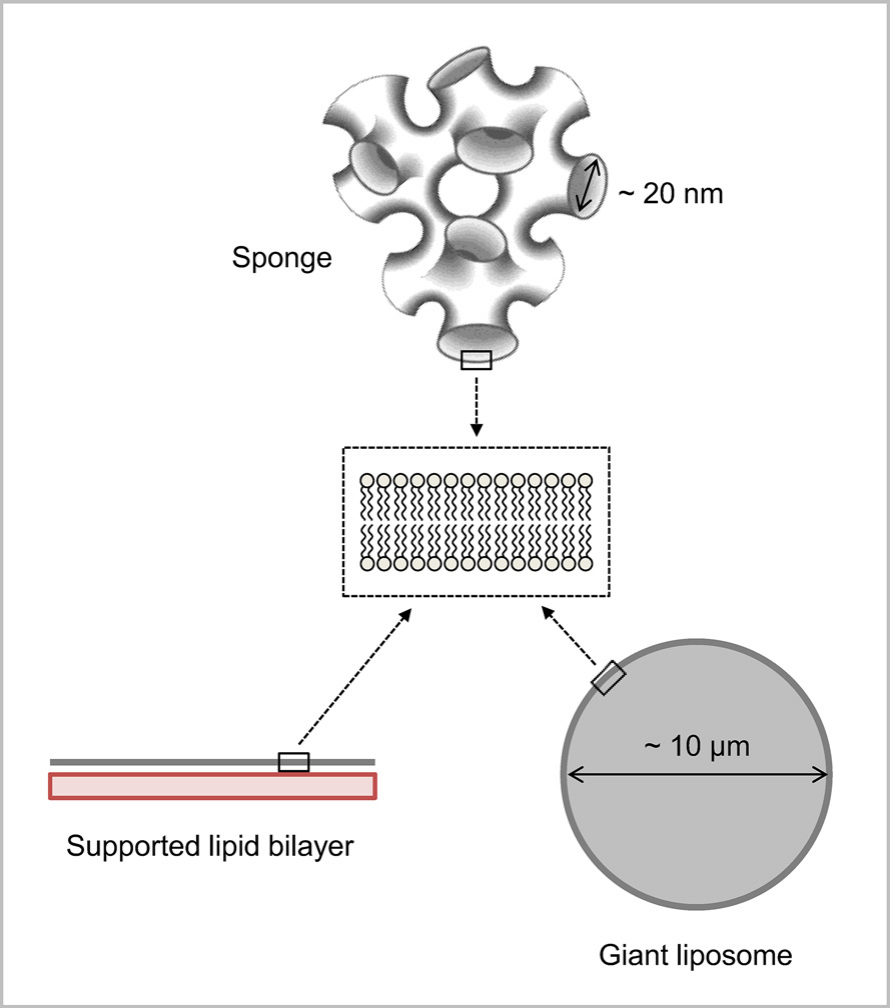
Various lipidic platforms used in this work to study the mobility and interaction of lipids and proteins within membranes. Supported lipid bilayers are formed by the Langmuir-Blodgett deposition technique, which allows the formation of asymmetrical membranes (inner and outer monolayers can be of different lipid compositions). Giant liposomes are free standing membranes of 10–100 μm diameter. Sponge phases consist of interconnected bilayers forming aqueous channels whose diameter can be varied from 6 to 30 nm.

Living cells are highly dynamic multi-compartment systems, whose main constituents (proteins and lipids) are in constant movement within and across compartments. This permanent intracellular motion is notably important for the proper localization and lateral organization of membrane proteins at their site of action. Various model lipidic platforms are now available to reconstitute and study *in vitro* the distribution and mobility of proteins within the plane of membranes, as well as their interaction with lipids and other (membrane or soluble) proteins [1,2]. These include supported lipid bilayers, giant liposomes and sponge phases (Fig 1) that all have specific advantages and limitations (Table 1). Supported lipid bilayers formed by the Langmuir-Blodgett deposition technique can mimic the asymmetric distribution of lipids between the two leaflets, as found in biological membranes. But the presence of the underlying substrate induces some friction forces, leading to a reduction of lateral diffusion and even the absence of mobility in the case of transmembrane proteins [1]. Alternative methods have been developed to address this problem, including the formation of bilayers on polymer cushions [3] or over holes [4]. This issue can also be overcome with giant liposomes that are free standing, micromanipulable, lipid bilayers. Lipid composition asymmetry is more difficult to recapitulate in this system although some recent double-emulsion and microfluidics approaches have allowed the reconstitution of fully functional transmembrane proteins into asymmetrical giant liposomes [5]. Sponge phases consist of a network of interconnecting model bilayers whose hydrophobic thickness and separating distance can be easily modulated, by adding the appropriate (hydrophobic or aqueous) solvent [6,7]. This system thus provides a powerful tool to follow the mobility of transmembrane proteins, as well as their interactions within or across membranes [8,9].

**Table 1:** Advantages and disadvantages of the various *in vitro* membrane platforms available to study FRAP by confocal microscopy.

	Supported lipid bilayers	Giant liposomes	Sponge phases
**Bleaching geometry**	Disks	Disks	Disks
	Fringes		Fringes
**Molecules**	Lipids	Lipids	Lipids
	Lipid-bound proteins	Lipid-bound proteins	Lipid-bound proteins
		Transmembrane proteins	Transmembrane proteins
			Soluble proteins
**Advantages**	Bilayer asymmetry	Free-standing membrane	Free-standing membrane
		Micromanipulable	Few molecules required
			Easy and fast preparation
**Disadvantages**	Not appropriate for transmembrane proteins	Difficult to incorporate functional transmembrane proteins	Not exclusively made of lipids
	Friction with the substrate		

Fluorescence recovery after photobleaching (FRAP) measurements have been widely used to monitor the mobility and the interaction of fluorescently-labeled biological molecules within living cells as well as model membrane systems [10–12]. The FRAP methodology has become accessible to most laboratories about 20 years ago thanks to the development of (i) powerful, commercially available, confocal microscopes with the required temporal and spatial resolutions, and (ii) various labeling techniques based on genetic or chemical modifications of the molecule of interest. Yet, the methods of FRAP data acquisition and analysis may vary from one lab to another [13–18]. One major difficulty of FRAP is to accurately estimate the intrinsic photobleaching of the fluorescent molecules. This issue can be resolved by using fluorescence recovery after pattern photobleaching (FRAPP) in which the differential intensity between bleached (dark) and non-bleached (bright) regions is monitored. This procedure allows the experimentalist to record simultaneously fluorescence gain and fluorescence loss for the same molecules, and thus to suppress any contribution from intrinsic photobleaching. Another difficulty is to determine whether the movement of the molecules is Brownian or not. This can be done in both FRAP and FRAPP experiments by systematically varying the size of the bleached area. Finally, the identification of several mobile species corresponding, for instance, to various degrees of oligomerization or to molecules having different interactions with the membranes, can be more easily determined by FRAPP than by FRAP [11]. In this work, we propose some general guidelines to investigate the mobility and interaction of biological molecules by FRAP or FRAPP that can be easily implemented on all confocal microscopes equipped with imaging software and that are immediately applicable to characterize molecular diffusion and interaction in various artificial membrane platforms as well as in cellular contexts.

## Results & Discussion

### Optimization of FRAP data collection and analysis

**Fig 2:**
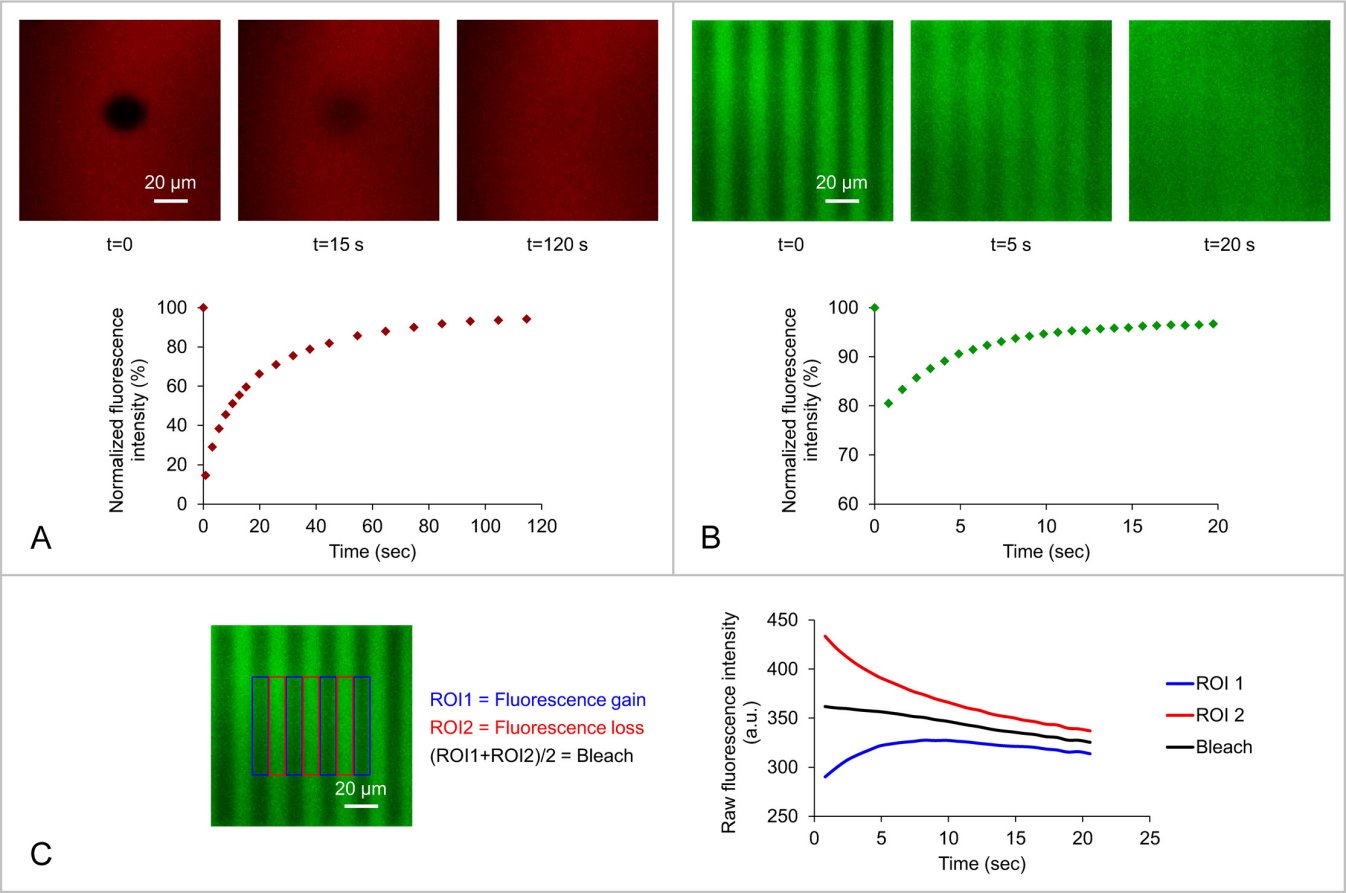
Fluorescence recovery of (A) DOPE-Rho or (B) DOPE-NBD lipids in the outer monolayer of a supported DOPC lipid bilayer following photobleaching of (A) a fluorescent disk of d = 20 μm diameter or (B) a pattern of fluorescent fringes separated by f = 20 μm. The recovery curve (result of 1 bleaching experiment on the same region in A and average of 3 bleaching experiments on the same region in B) can be fitted with (A) a Bessel function or (B) an Exponential function. (C) The contribution of the intrinsic photobleaching occurring during fluorescence reading (bleach) in the case of DOPE-NBD lipids can be removed in the fringe system by using the average of fluorescence gain in the dark fringes and fluorescence loss in the bright fringes. No intrinsic photobleaching was observed with DOPE-Rho lipids.

The imaging software of confocal microscopes allows the design of complex and precise bleaching patterns. In this work, we have generated two different bleaching geometries (regions of interest): a disk or a pattern of fringes to perform respectively FRAP or FRAPP experiments (Fig 2). A typical fluorescence recovery after photobleaching experiment consists of a bleaching phase obtained by exposing the region of interest (ROI) to a high-intensity laser beam, followed by a recovery phase monitored with a low-intensity laser beam. The recovery of fluorescence results from the diffusion of unbleached molecules toward the ROI and bleached molecules out of the ROI. The shape of the recovery curve depends on the mobility of the molecules, but also on the form and size of the bleached area.

In the case of a disk-shaped bleaching geometry (FRAP experiments, Fig 2A), the recovery process over the whole bleached area is described by the function [19,20]:

$$I\left(t\right)=I_{0}+\sum \left(I_{n\infty }-I_{0}\right)\;e^{-\frac{{2\tau }_{n}}{t}}\left(J_{0}\left(\frac{{2\tau }_{n}}{t}\right)+J_{1}\left(\frac{{2\tau }_{n}}{t}\right)\right)$$ (1)

Where I_0_ is the fluorescence intensity just after the bleach, J_0_ and J_1_ are the modified Bessel functions of order 0 and 1, I_n∞_ is the intensity contribution of species n at t = ∞ and τ_n_ is the characteristic diffusion time of species n. The details of the calculation leading to Eq 1 are given in reference [19].

Such fitting procedure is quite complex, notably because it requires the determination of three parameters: I_0_, I_∞_ and τ (in the simple case where there is only one diffusive species). In order to optimize data acquisition and fitting, we have used *in silico* simulations of FRAP experiments [21,22]. These tests (see methods section and S1–S3 Figs for details) have allowed us to show that, assuming there is a single diffusive species and a typical signal-to-noise ratio of 10: (i) the fluorescence should be monitored during at least 6 times the characteristic diffusion time τ, and (ii) the image acquisition frequency (frame rate) should be at least 2/τ. One should note that, in the various experiments we have performed with disk-shaped bleaching geometries, we did not succeed in accurately differentiating several species with Eq 1. Nevertheless, our simulations predict that this should be feasible in FRAP experiments with very good signal-to-noise ratios (greater than 500) and for species whose characteristic diffusion times differ by at least a factor of 4 (see S4 Fig for details).

In the case of a fringe-shaped bleaching geometry (FRAPP experiments, Fig 2B), the fluorescence signal is recorded both in the bleached and unbleached fringes (Fig 2C), which allows removing the contribution from intrinsic bleaching that inevitably occurs, even at low-intensity laser beam, with photosensitive probes. Fluorescence gain is recorded in the dark fringes (region of interest 1, ROI 1) and fluorescence loss is recorded in the bright fringes (region of interest 2, ROI 2). The intrinsic photobleaching occurring during fluorescence reading is then given by:

$$I_{b}\left(t\right)\;=\frac{\left(I_{ROI1}+I_{ROI2}\right)}{2}$$ (2)

And the corrected intensity is therefore:

$$I_{c}\left(t\right)\;=\frac{I_{ROI2}\left(t\right)}{I_{b}\left(t\right)}$$ (3)

I_c_(t) then follows [23]:

$$I_{c}\left(t\right)=I_{0}+\sum I_{n\infty }\;\left(1-e^{-\frac{t}{\tau_{n}}}\right)$$ (4)

Because exponential fits are much easier to perform than Bessel function fits, it is possible to determine the presence of multiple species in FRAPP experiments with signal-to-noise ratios greater than 200 as long as the characteristic diffusion times differ by at least a factor of 2 (see S5 Fig for details).

Two important parameters can be deduced from the fluorescence recovery curves described by Eq 1 in the case of FRAP and Eq 4 in the case of FRAPP: (i) the kinetics of recovery (characterized by τ) that gives access to the mobility of the studied molecules, and (ii) the extent of recovery that provides information about the potential presence of immobile molecules within the sample.

For FRAP experiments described by Eq 1, the immobile fraction is given by:

$$F_{imm}\;=\frac{I_{init}-\sum I_{n\infty }}{I_{init}-I_{0}}$$ (5)

Where I_init_ is the intensity before the bleach.

In the case of FRAPP experiments described by Eq 4, it is given by:

$$F_{imm}=\frac{1-\sum I_{n\infty }}{1-I_{0}}$$ (6)

The immobile fraction is easily visualized in FRAPP experiments. Indeed, when there is no immobile fraction, I_ROI1_(t) and I_ROI2_(t) must converge to the same value. For instance, in Fig 2C, the fact that I_ROI1_(t) and I_ROI2_(t) are parallel to the intrinsic photobleaching curve I_b_(t) but do not converge to I_b_(t) at infinite time shows that there is a fraction of immobile molecules.

### Diffusion coefficient measurement

Recovery after photobleaching can occur through various relaxation processes, including Brownian diffusion, convection or even reversible photobleaching of the fluorescent probe. The measurement of a single characteristic diffusion time is not enough to discriminate between these different phenomena. In order to confirm that diffusion is driven by pure Brownian motion, one needs to monitor fluorescence recovery in ROI of various dimensions [24], *i*.*e*. disks of various diameters d, or networks of fringes with various interfringes f (defined as the distance between the centers of two consecutive bright fringes). For both disk- and fringe-shaped bleaching geometries, the shape of the plot s^2^ (d^2^ or f^2^) *versus* τ indicates the type of diffusion. It is usually a straight line whose intercept with the time-axis allows the determination of the diffusion mechanism, as established by simulation of Fluorescence Correlation Spectroscopy (FCS) experiments [25,26]. If this straight line passes through the origin, the diffusion is controlled by pure Brownian motion. If the intercept with the time-axis, τ_0_, is not 0, the diffusion is not perfectly Brownian. When τ_0_ > 0, the observed behavior can be explained by the presence of confinement micro-domains in which the labeled molecule is transiently trapped. In this case, the molecule has a Brownian motion over a short time scale within the micro-domain (the characteristic confinement time is directly related to τ_0_), and needs to cross an energy barrier to freely diffuse to another micro-domain. Such diffusion process is observed for instance with molecules confined in membrane raft domains [26]. When τ_0_ < 0, the diffusion mechanism can be interpreted by the presence of a meshwork that hinders diffusion: the molecules have to cross a physical barrier to visit another mesh. This occurs for example with molecules of the plasma membrane that are trapped in cytoskeletal corrals [26]. In the various platforms we used here with model membranes, we always observed τ_0_ = 0. In this case, the slope of the plot s^2^ (d^2^ or f^2^) *versus* τ directly gives the diffusion coefficient, D.

In the case of a disk-shaped bleaching geometry (FRAP experiments) [19]:

$$D=\frac{d^{2}}{16\tau }$$ (7)

Where d is the disk diameter.

In the case of a fringe-shaped bleaching geometry (FRAPP experiments) [23]:

$$D=\frac{f^{2}}{4\pi^{2}\tau }$$ (8)

Where f is the interfringe.

**Fig 3:**
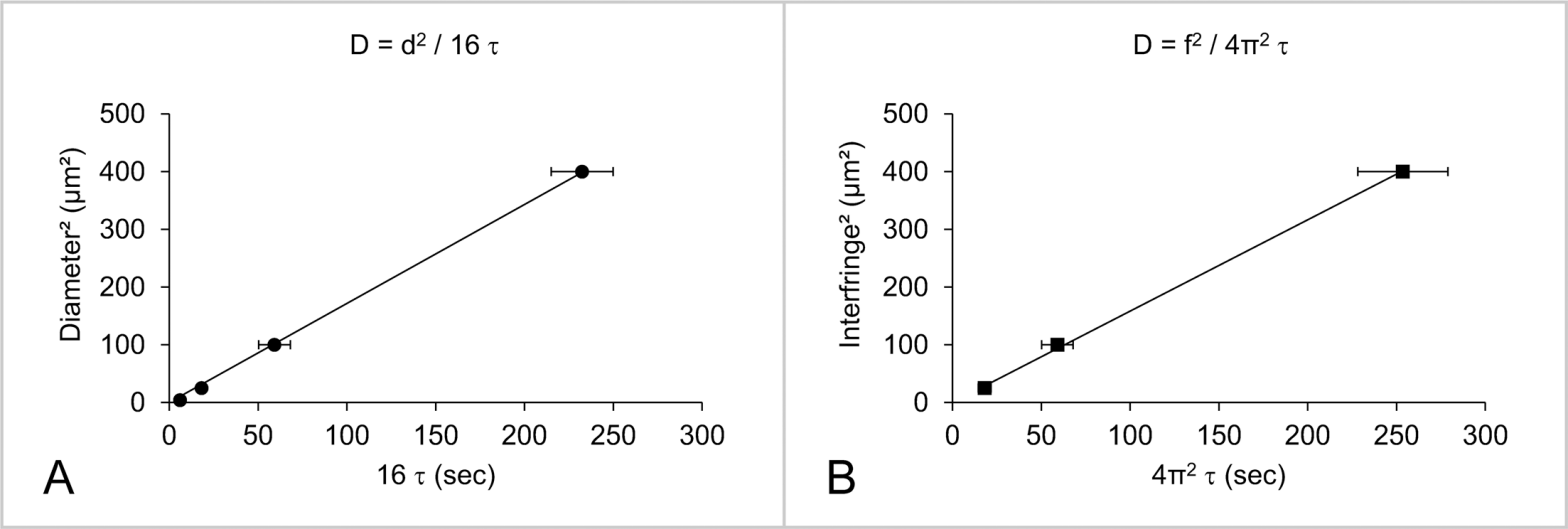
Diffusion coefficient (D) of DOPE-Rho lipids in the outer leaflet of a supported DOPC lipid bilayer deduced by varying the area of the bleached region (disks of diameters d = 2, 5, 10 or 20 μm in A; networks of fringes with an interfringe f = 5, 10 or 20 μm in B). The linear relationship between the bleaching area s^2^ (d^2^ or f^2^) and the recovery time (τ) proves that lipid diffusion is controlled by Brownian motion. The diffusion coefficient (D) is calculated from the slope of this straight line: D = d^2^ / 16 τ in the case of the disk system, and D = f^2^ / 4π^2^ τ in the case of the fringe system. Each data point in A or B is the average of N = 3 independent bleaching experiments on different regions of the same bilayer, and the error bars correspond to the standard deviations from these averages. These experiments were reproduced with 4 independent (freshly prepared) bilayers using either the disk- or the fringe-shaped bleaching geometry, leading to D = (1.9 ± 0.3) μm^2^/s.

As an example, using a glass supported DOPC bilayer, we have monitored fluorescence recovery of DOPE-Rho lipids in its outer leaflet after bleaching with disks or fringes of various sizes. For both geometries, the lipid follows unrestricted Brownian motion with a diffusion coefficient D = (1.9 ± 0.3) μm^2^/s (Fig 3 and Table 2). Similar results were obtained with DOPE-NBD lipids that also diffused at D = (1.9 ± 0.4) μm^2^/s in the outer layer of a supported DOPC bilayer (Table 2), in agreement with previous studies using FRAP [3,27] or FCS experiments [28].

**Table 2:** Diffusion coefficient of various headgroup-labeled DOPE lipids in different artificial membrane environments. Two-sample *t*-tests: supported lipid bilayers vs. giant liposomes (p<0.01); supported lipid bilayers vs. sponge phases (p<0.001); giant liposomes vs. sponge phases (non-significant).

	Supported bilayers (μm^2^/s)	Giant liposomes (μm^2^/s)	Sponge phases (μm^2^/s)
**DOPE-Rho**	1.9 ± 0.3	3.7 ± 0.5	4.1 ± 0.4
**DOPE-NBD**	1.9 ± 0.4	3.4 ± 0.7	4.2 ± 0.8
**DOPE-CF**	N/A	3.7 ± 0.4	5.5 ± 0.1

Either the disk- or the fringe-shaped bleaching geometries can thus be used to characterize the mobility of fluorescent molecules within supported lipid bilayers. The fringe pattern presents two main advantages: (i) to accurately remove the contribution from intrinsic photobleaching through the simultaneous measurement of fluorescence in the bright and dark fringes (as illustrated in Fig 2C with DOPE-NBD lipids), and (ii) to more easily identify several diffusive species through the shape of the recovery curve (simple exponential in the case of a single diffusing species and multiple exponential for several diffusing species). This geometry could also be used to follow the mobility of molecules within the plasma membrane of cells *ex vivo*. However, because the bleaching phase is performed over a large area, one cannot always bleach efficiently highly photostable molecules, such as DOPE-Rho. In addition, such large bleaching area might not be appropriate when working with smaller structures such as giant liposomes *in vitro* or intracellular compartments *ex vivo* (*e*.*g*. nucleus, endoplasmic reticulum, Golgi apparatus), whose observable surface might not accommodate the fringe pattern. In this case, the disk-shaped geometry is better suited.

### Influence of the substrate

**Fig 4:**
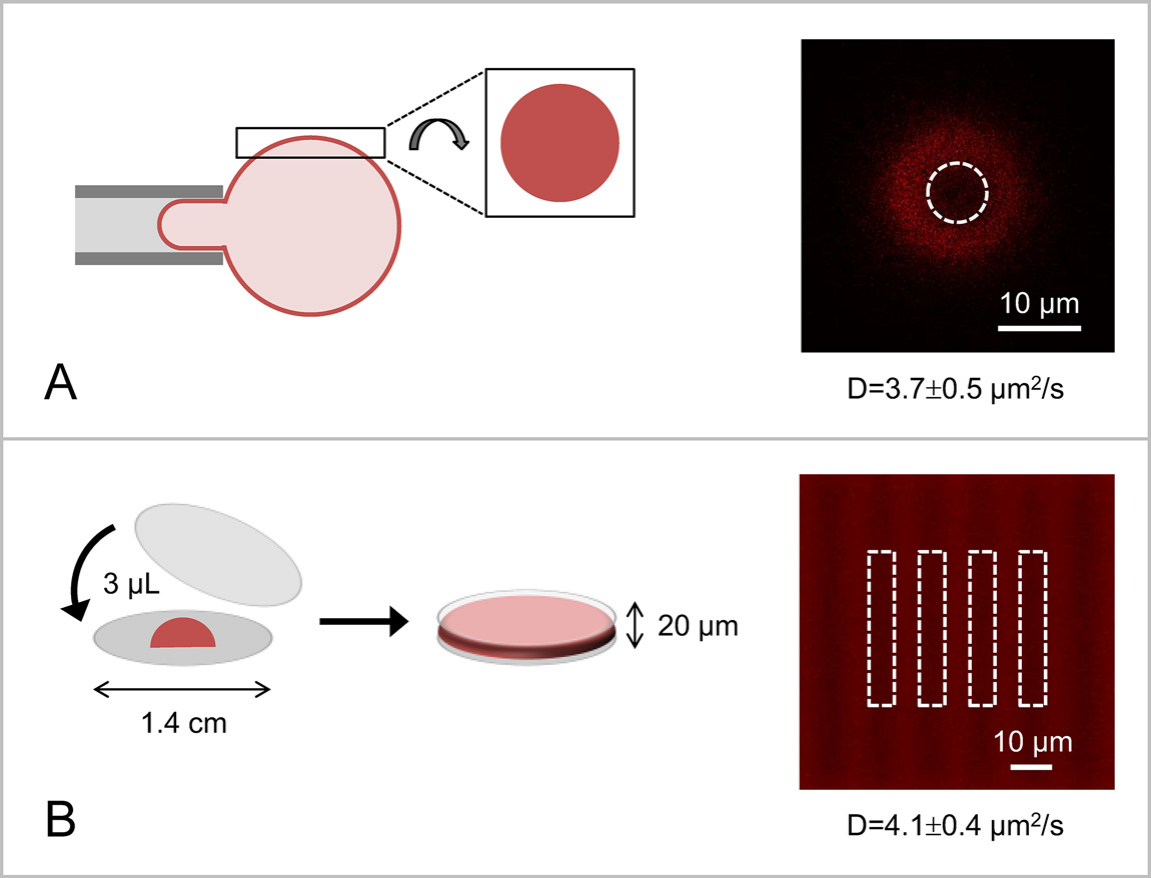
Experimental set-up used to perform FRAP experiments on (A) giant liposomes or (B) sponge phases. (A) To prevent liposome movement during FRAP measurements, giant liposomes are confined in a closed chamber of ~500 μm height (not shown here) or ideally (as shown here) held through micromanipulation. Fluorescence bleaching and recovery measurements are performed at the top (pole) of the giant liposome, which appears as a fluorescent disk in the confocal microscope. In this system, FRAP measurements are performed using the disk-shaped geometry. To ensure that the bleached spherical cap can be treated as a disk, the diameter of the bleaching disk should not exceed 25% of the giant liposome diameter. The confocal picture on the right shows the result of bleaching a 7 μm diameter fluorescent disk in the membrane of a DOPC:DOPE-Rho (99:1) giant liposome of 45 μm diameter. (B) Sponge phases (3 μL solution) are sandwiched between two glass surfaces (1.5 cm diameter) to form a liquid layer of ~20 μm height; this allows bleaching at all z-values within the sponge phase and thus measuring 2-dimensional fluorescence recovery. Here, bleaching is performed using the fringe-shaped geometry (interfringe f = 12 μm). The two systems were used to study the mobility of DOPE-Rho lipids. In both cases, DOPE-Rho lipids diffused faster than in supported lipid bilayers (D = 3.7 ± 0.5 μm^2^/s in the membrane of giant liposomes and D = 4.1 ± 0.4 μm^2^/s in sponge phases), which we attribute to the reduction of friction forces between the outer and the inner leaflets when the membrane is not linked to a solid substrate.

In order to compare the mobility of lipids in the various available artificial membrane platforms, we have next reconstituted DOPE-Rho lipids in giant DOPC liposomes and in sponge phases made of the nonionic surfactant pentaethylene glycol monododecyl ether, C_12_E_5_. FRAP measurements on giant liposomes were conducted at the top of liposomes, where the surface is seen as a disk of finite size by fluorescence microscopy. To prevent their movement, giant liposomes were either held by a micropipette (ideal) or confined within a closed chamber of ~500 μm height (Fig 4A). DOPE-Rho lipids diffused at D = (3.7 ± 0.5) μm^2^/s within the membrane of giant DOPC liposomes, which is about 2 times faster than in supported lipid bilayers in good agreement with previous studies [29]. FRAPP experiments on sponge phases were performed on ~20 μm thick sample in order to be able to bleach at all z-values within the sponge phase (using a 20X objective; see the method section for details) and thus to measure 2-dimensional fluorescence recovery (Fig 4B). In this system, DOPE-Rho lipids moved also faster than in supported lipid bilayers, with a diffusion coefficient D = (4.1 ± 0.4) μm^2^/s. Comparable results were obtained with other headgroup labeled phosphatidylethanolamine lipids, namely DOPE-NBD and DOPE-CF (Table 2). These values obtained by FRAP and FRAPP are consistent with measurements of lipid mobility in giant liposomes using FCS [30] or Single Particle Tracking (SPT) of quantum dots coupled to lipids [31]. One should note that, because of the complex topology of the sponge phase, the measured diffusion coefficient in this system is underestimated by a factor of at least 3/2 [32]. We attribute the slower diffusion rate measured in supported lipid bilayers to the contribution of friction forces between the outer and the inner leaflets when the bilayer is closely apposed to a solid substrate [33]. When transmembrane proteins are reconstituted into supported lipid bilayers, these friction forces can even lead to loss of protein mobility [1,3]. In this case, it is therefore recommended to work with free-standing systems, such as giant liposomes [30,31,34] or sponge phases [8], which do not impede the lateral diffusion of transmembrane domains.

### Application to SNARE proteins interaction

Sponge phases present the further advantage of allowing the measurement of soluble proteins diffusion within the sponge channels, and their potential interaction with components of the sponge membrane [9,35] (lipids, lipid-bound proteins, transmembrane proteins, *etc*.). To illustrate this, we have used sponge phases to study how the interaction between the t-SNARE protein, Syn1A, and its cognate v-SNARE protein, VAMP2, is modulated by the regulatory protein Munc18-1. SNARE proteins constitute the core molecular machinery of intracellular membrane trafficking: v-SNARE proteins residing in the membrane of transport vesicles assemble, in a zipper-like fashion, with their partner t-SNARE proteins residing in the target membrane, to form a highly stable membrane-bridging complex, the SNAREpin that triggers close membrane apposition and fusion [36–38]. In many instances, the specificity and spatial integrity of these fusion events is conferred by proteins from the Sec1/Munc18 (SM) family. The crystal structure of SM proteins revealed a horseshoe geometry with the central cavity being involved in SNARE binding [39]. The neuronal SM protein Munc18-1 binds to the closed (fusion inactive) form of the t-SNARE protein Syn1A [40], but also to the assembling SNAREpin consisting of the v-SNARE protein VAMP2 and the t-SNARE complex Syn1A/SNAP25 (with Syn1A in its open form) [41,42]. The first binding mode may prevent Syn1A from undesirable interactions during its synthesis and transport toward the target membrane [43], whereas the second binding mode may stabilize SNAREpins and thus facilitate fusion [42].

**Fig 5:**
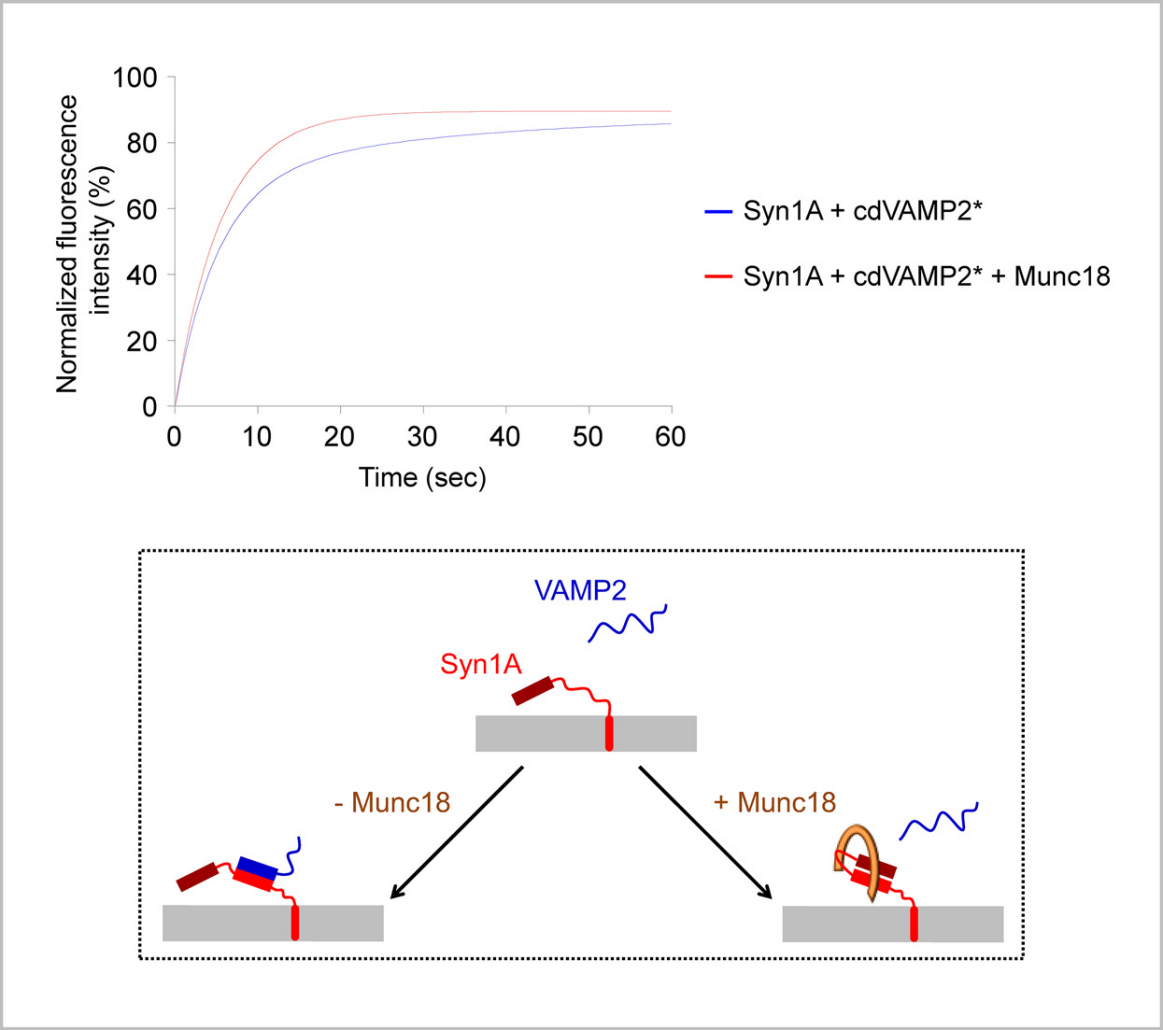
Interaction between SNARE proteins in a sponge phase. The full length Syn1A protein and the cytoplasmic domain of FITC-labeled VAMP2 protein (cdVAMP2*) were reconstituted into two separate sponge phases (at the respective lipid-to-protein molar ratios of 20,000 and 80,000). The Syn1A sponge was pre-incubated for 1 hour at room temperature with or without Munc18-1 protein (1:1 molar ratio between Syn1A and Munc18-1). The Syn1A ± Munc18-1 sponge and the cdVAMP2* sponge were then mixed and allowed to interact for 1 hour at room temperature. In the absence of Munc18-1 (blue fitting curve), cdVAMP2* displays two diffusion coefficients: a slow diffusion coefficient (2.0 ± 0.1 μm^2^/s) corresponding to cdVAMP2* bound to Syn1A in the sponge membrane and a fast diffusion coefficient (9.7 ± 1.4 μm^2^/s) corresponding to free (unbound) cdVAMP2* in the sponge channels. In the presence of Munc18-1 (red fitting curve), cdVAMP2* displays a single, fast, diffusion coefficient (6.4 ± 1.1 μm^2^/s), as observed when it is added to a protein-free sponge phase, showing that Munc18-1 inhibits the interaction between Syn1A and cdVAMP2* (see also Table 3).

To study the interaction between Syn1A and VAMP2, we have mixed a sponge phase containing unlabeled Syn1A in its membrane with a sponge phase containing or not the cytoplasmic domain of FITC-labeled VAMP2 (FITC-cdVAMP2) in its channels. In their respective sponge phase, FITC-labeled Syn1A and cdVAMP2 diffused as a single species at D_FITC-Syn1A_ = (2.3 ± 0.6) μm^2^/s and D_FITC-cdVAMP2_ = (5.5 ± 0.8) μm^2^/s (Table 3). The diffusion coefficient of cdVAMP2 is slightly lower than expected for a soluble protein [8], suggesting that it interacts, at least transiently, with the sponge membrane. This is consistent with the previously described transient association of the cytoplasmic domain of VAMP2 with the lipid bilayer interface [44]. When added to an unlabeled Syn1A sponge, FITC-cdVAMP2 displayed two diffusion coefficients: D_slow/FITC-cdVAMP2_ = (2.0 ± 0.1) μm^2^/s and D_fast/FITC-cdVAMP2_ = (9.7 ± 1.4) μm^2^/s (Fig 5A and Table 3). The slower diffusion coefficient corresponds to cdVAMP2 molecules that interact with Syn1A molecules and thus diffuse as a membrane-embedded protein. The faster diffusion coefficient corresponds to unbound cdVAMP2 molecules. These molecules move faster than in Syn1A-free sponges, which we attribute to a reduction of the cdVAMP2/membrane interaction when Syn1A is present in the sponge bilayer. Interestingly, when Syn1A was pre-incubated with Munc18-1 prior to cdVAMP2 addition, the slower diffusion coefficient disappeared and cdVAMP2 diffused as a single species at D_FITC-cdVAMP2_ = (6.4 ± 1.1) μm^2^/s (Fig 5A and Table 3). This shows that the Syn1A/cdVAMP2 interaction is inhibited in the presence of Munc18-1 (Fig 5B), consistent with previous work [45–47].

**Table 3:** Interaction parameters (diffusion coefficients and mobile fractions) of Syn1A and cdVAMP2 in a sponge phase, in the absence or presence of Munc18-1. FITC-labeled molecules are indicated with a star. Two-sample *t*-tests on fast diffusion components: cdVAMP2* vs. Syn1A+cdVAMP2* (p<0.05); cdVAMP2* vs. Syn1A+cdVAMP2*+Munc18 (non-significant); Syn1A+cdVAMP2* vs. Syn1A+cdVAMP2*+Munc18 (p<0.05). Two-sample *t*-test on slow diffusion components: Syn1A* vs. Syn1A+cdVAMP2* (non-significant).

	Syn1A*	cdVAMP2*	Syn1A+cdVAMP2*	Syn1A+cdVAMP2*+Munc18
**D/fast (μm**^**2**^**/s)**	N/A	5.5 ± 0.8	9.7 ± 1.4	6.4 ± 1.1
**Mobile/fast (%)**	N/A	97 ± 1	64 ± 4	87 ± 4
**D/slow (μm**^**2**^**/s)**	2.3 ± 0.6	N/A	2.0 ± 0.1	N/A
**Mobile/slow (%)**	99 ± 1	N/A	28 ± 8	N/A

FRAPP experiments performed on proteins in a sponge phase thus constitute a powerful tool to investigate protein/protein interaction through the change of their diffusion coefficient, for example when they transit from a soluble to a membrane-bound form [9] (as in the example above).

## Conclusion

Fluorescence recovery after photobleaching (FRAP) is a powerful technique to investigate the lateral mobility, distribution and interaction of lipids and proteins in artificial membrane systems as well as in living cells. In order to obtain accurate measurements that can be compared to those obtained using other techniques, one must however be careful with the way to collect and analyze data. Using *in silico* simulations, we have shown that FRAP data should be recorded during at least 6 times the characteristic diffusion time τ, and that the image acquisition frequency should be at least 2/τ. The diffusion coefficient should be deduced from several FRAP experiments using different bleaching areas. The proportionality between the characteristic diffusion time τ and the bleaching area s^2^ ensures that the mobility is controlled by pure Brownian motion, and the slope of the plot directly provides the diffusion coefficient, D. Using these guidelines for data collection and analysis, we have characterized the mobility of lipids in various artificial membrane platforms (supported lipid bilayers, giant liposomes and sponge phases) and deduced D values in good agreement with those previously obtained by fluorescence correlation spectroscopy (FCS) and single particle tracking (SPT). These experiments also showed that the disk- and the fringe-shaped bleaching geometries lead to the same results, proving that they can both be used to study the diffusion of molecules with a confocal microscope. Here, we have used the fringe-shaped bleaching geometry to study the 3-way interaction, in sponge phases, between the SNARE proteins, Syn1A and VAMP2, and their regulator, Munc18-1. Similar procedures, using either geometry (disk or fringe, depending on the topology of the studied compartment), should be followed when performing FRAP experiments on living cells [48]. In a cellular context, it would notably be interesting to use the law τ = f (s^2^) deduced from FRAP experiments on lipids or proteins to characterize the mechanisms underlying their diffusion process (purely Brownian, restricted by lipid micro-domains or trapped in a cytoskeleton-mediated meshwork, *etc*.) as previously done using FCS experiments [26].

## Materials and Methods

### Chemicals

The lipids used in this study– 1,2-dioleoyl-*sn*-glycero-3-phosphocholine (DOPC), 1,2-dioleoyl-*sn*-glycero-3-phosphoethanolamine-N-(7-nitro-2-1,3-benzoxadiazol-4-yl) (ammonium salt) (DOPE-NBD), 1,2-dioleoyl-*sn*-glycero-3-phosphoethanolamine-N-(lissamine rhodamine B sulfonyl) (ammonium salt) (DOPE-Rho) and 1,2-dioleoyl-*sn*-glycero-3-phosphoethanolamine-N-(carboxyfluorescein) (ammonium salt) (DOPE-CF)–were purchased as chloroform solutions from Avanti Polar Lipids. 4-(2-hydroxyethyl)piperazine-1-ethanesulfonic acid (HEPES), potassium hydroxide (KOH), potassium chloride (KCl), glycerol, octyl β-D-glucopyranoside (β-OG), tris(2-carboxyethyl)phosphine hydrochloride (TCEP), and pentaethylene glycol monododecyl ether (C_12_E_5_) were purchased from Sigma-Aldrich with the BioChemika Ultra grade for molecular biology. All aqueous solutions were prepared using 18.2 M ultra-pure water and filtered through 0.2 μm hydrophilic membranes.

### Protein purification and labeling

Syn1A (full length rat Syn1A-His_6_), cdVAMP2 (the cytoplasmic domain of mouse His_6_-VAMP2) and Munc18-1 (full length rat Munc18-1) proteins were expressed in the BL21(DE3) *Escherichia coli* bacterial strain and purified by nickel affinity chromatography as described previously [34,38,49]. After purification, Syn1A was dialyzed against (25 mM HEPES/KOH, pH 7.7; 100 mM KCl; 10% (v/v) Glycerol; 1% β-OG; 0.25 mM TCEP); cdVAMP2 and Munc18-1 were dialyzed against (25 mM HEPES/KOH, pH 7.7; 100 mM KCl; 10% (v/v) Glycerol; 0.25 mM TCEP). Syn1A and cdVAMP2 were labeled with fluorescein-5-isothiocyanate (FITC, Molecular Probe) following the manufacturer's instructions. Protein aliquots (100 μL at ~100 μM) were mixed with 10 μL sodium bicarbonate at 1M; FITC was dissolved at 25 mM in Dimethyl sulfoxide (DMSO) and 10 μL was added to the protein sample (1 mol of protein for 25 mol of FITC). The reaction was incubated for 2 hours at room temperature with continuous stirring. Free FITC was separated from FITC-protein complex on a Sephadex G-50 column equilibrated with the protein buffer, and the degree of labeling (DOL) was determined using absorption measurements at 280 nm and 494 nm using the following equation:

$$DOL=\frac{\varepsilon_{protein}}{\varepsilon_{FITC}}\frac{A_{494\;complex}}{\left(A_{280\;complex}-\frac{A_{280\;free\;FITC}}{A_{494\;free\;FITC}}A_{494\;complex}\right)}$$ (9)

Using this protocol, we could typically obtain 1 mL of FITC-labeled SNARE proteins at ~10 μM with a DOL of 0.8–1, suggesting that FITC specifically labeled the N-terminus of SNARE proteins.

### Preparation of artificial membrane platforms

Supported lipid bilayers were formed onto the glass-bottom part of a petri dish using the Langmuir-Blodgett deposition technique [50]. Glass-bottom petri dishes (35 mm dishes from MatTek with uncoated glass cover slip of 14 mm diameter, thickness number 1.5) were soaked for 1 hour at ~ 60°C in 2% v/v cleaning detergent (MICRO-90, VWR), thoroughly rinsed with 18.2 MΩ ultra-pure water, and then dried under a stream of nitrogen. A chloroform solution of the lipid mixture constituting the inner monolayer was first spread on degassed water in a NIMA Langmuir trough (model 611 equipped with the PS4 surface pressure sensor) and allowed to dry for 15 min at room temperature. After solvent evaporation, the film was compressed up to 35 mN/m and the monolayer was transferred onto the glass cover slip (with its lipid headgroups facing the glass surface) as the petri dish was slowly (0.5 cm/min) raised out of water. This first monolayer was allowed to dry for 15 min at room temperature. Meanwhile, a chloroform solution of the lipid mixture constituting the outer monolayer was spread at the air/water interface. After 15 minutes, this second monolayer was transferred at a constant pressure of 35 mN/m and with a dipping speed of 0.5 cm/min onto the first monolayer (with its lipid headgroups facing the aqueous medium). The glass-bottom dish was next carefully transferred into a beaker containing 1 L of buffer A (25 mM HEPES/KOH, pH 7.7; 100 mM KCl; 10% v/v glycerol), the bilayers remaining immersed in aqueous medium throughout. Buffer exchange proceeded for 15 minutes and then the glass-bottom dish was moved out of the beaker.

Giant liposomes were generated using the electroformation method [51] in a 3X3 cm^2^ chamber consisting of two indium tin oxide (ITO) conductive plaques (30 Ω) separated by a ~2 mm polydimethylsiloxane (PDMS) spacer. 20 μL of a 500 μM lipid mixture (in chloroform) was deposited onto the conductive side of one of the two ITO surfaces by drops of 1 μL, and dried under vacuum for 1 hour. The second ITO plaque was then placed on top of the PDMS spacer, with its conductive side facing the lipid films. The dried lipid films were rehydrated with 2 mL of sucrose at 220 mOsm in 10% v/v glycerol, and the following sinusoidal electrical fields were applied between the two ITO plaques: 8 Hz, 50, 100, 200, 300, 500, 700 and 900 mV for 5 min each; 8 Hz, 1.1 V for 2 hours; 4 Hz, 1.5 V for 30 min. Giant liposomes were kept overnight at 4°C before being harvested, and were observed in buffer A (25 mM HEPES/KOH, pH 7.7; 100 mM KCl; 10% v/v glycerol). This buffer is slightly hyper-osmotic (equivalent of 230 mOsm in 10% v/v glycerol) compared to the sucrose solution, which deflates the giant liposomes and thus allows their micromanipulation. Samples were prepared in a glass-bottom MatTek petri dish (35 mm dishes with uncoated glass cover slip of 14 mm diameter, thickness number 1.5), pre-coated with 10% w/v BSA for 30 min at room temperature (to prevent giant liposomes from sticking to the glass surface). 50 μL of giant liposomes was added to 250 μL of buffer A and allowed to sediment for 15 min at room temperature. The central 300 μL drop was next slowly grown to 3 ml by addition of buffer A to its edge. FRAP experiments were performed on giant liposomes that were either confined in a closed chamber of ~500 μm height or held by a micropipette. The closed chamber was made by putting a 25 mm diameter cover slip on top of the central well. The micropipette (~8 μm in external diameter) was incubated with 10% w/v BSA during 30 min at room temperature and then rinsed with buffer A. Giant liposomes were caught by the micropipette using a slight aspiration, and were lifted ~50 μm off the bottom of the dish. The focus was first made at the equator of the giant liposome, where the fluorescent membrane appears clearly detachable from the background, taking the form of a circle whose diameter can easily be measured. Then, the focus was made above the giant liposome’s equatorial plan at a distance of approximately the radius of the giant liposome minus 5 μm. At this focus, only the homogeneously fluorescent pole of the giant liposome is observable and is seen as a fluorescent disk.

Sponge phases were prepared as a mixture between water, the non-ionic surfactant pentaethylene glycol monododecyl ether (C_12_E_5_) and the co-surfactant octyl β-D-glucopyranoside (β-OG). Such surfactant bilayers were shown to fully preserve the activity of transmembrane proteins [52]. In practice, the molar ratio n(C_12_E_5_)/n(β-OG) is always kept constant (at 6.9, which corresponds to 100 μg of β-OG for 1 μL of C_12_E_5_), whereas the volume ratio V(C_12_E_5_)/V(aqueous buffer) can be varied and determines the size of the sponge channels. For example, sponges of 13 nm or 24 nm channels (as in Figs 4 and 5, respectively) were obtained with 9% v/v (respectively, 4% v/v) of C_12_E_5_ (at 963 mg/ml). Sponge phases with large channels (24 nm) are preferred when working with proteins in order to prevent interactions between proteins embedded in two opposing bilayers. To prepare 13 nm sponge phases containing 0.5 mol% of lipids (molar ratio between the lipid and C_12_E_5_), 0.1 μmol of the lipid to be incorporated was dried for 10 min under a gentle stream of argon, and for 1 hour under vacuum. 9 μL of C_12_E_5_ was added to 1 μL of buffer A + 90 μL of buffer B (25 mM HEPES/KOH, pH 7.7; 100 mM KCl; 10% v/v glycerol; 1% w/v β-OG), and vortexed for ~10 s at room temperature. The lipid film was directly resuspended with this mixture by shaking gently for 5 min at room temperature. To prepare 24 nm sponge phases containing proteins, 4 μL of C_12_E_5_ was added to 56 μL of buffer A with 0.25 mM TCEP + 30 μL of buffer B with 0.25 mM TCEP, and vortexed for ~10 s at room temperature. 10 μL of proteins at the appropriate concentration was added to this mixture and shook gently for 5 min at room temperature. 3 μL of these sponge phases were added to the glass-bottom part of a MatTek petri dish (35 mm dishes with uncoated glass cover slip of 14 mm diameter, thickness number 1.5) and covered with a 14 mm cover slip sealed to the dish bottom with a Parafilm M® (to prevent evaporation).

### Fluorescence recovery after (pattern) photobleaching (FRAP and FRAPP) assays

FRAP experiments were performed on the confocal microscope TCS SP2 from Leica equipped with the LCS software, using either (i) an HCX PL APO 63X OIL objective (numerical aperture: 1.40; zoom: 3X; beam expander: 3; pinhole: 200 μm; 1 picture every 800 ms), when working with supported lipid bilayers and giant liposomes, or (ii) an HC PL APO 20X IMM objective (numerical aperture: 0.70; zoom: 10X; beam expander: 3; pinhole: 200 μm; 1 picture every 400 ms), when working with sponge phases. Fluorescence bleaching and recovery were conducted as follows.

For Rho: λ_exc_ = 543 nm; λ_em_ = 550–670 nm with 3 scans at 100% laser power for bleaching, and monitoring recovery at 10% of the maximum laser power.

For NBD: λ_exc_ = 458 nm; λ_em_ = 465–590 nm with 1 scan at 100% laser power for bleaching, and monitoring recovery at 5% of the maximum laser power.

For CF and FITC: λ_exc_ = 488 nm; λ_em_ = 495–650 nm with 1 scan at 100% laser power for bleaching, and monitoring recovery at 5% of the maximum laser power.

Recovery curves (average over at least 3 independent experiments, *i*.*e*. performed on a different region of the sample using the same bleaching conditions) were fitted with either the softwares Mathematica and Scilab (in the case of FRAP experiments, described by modified Bessel functions; code provided upon request) or the software Kaleidagraph (in the case of FRAPP experiments, described by exponential functions), using the method for non-linear least squares problems (Levenberg-Marquardt algorithm).

In order to take into account the contribution of fluorescence recovery that occurs during the photobleaching phase and can thus affect data analysis [53], the time t = 0 of the recovery phase in all FRAP and FRAPP experiments was set as the time of the last bleaching frame. We show in the Supporting Information (S1 Text) that this approximation is valid using the FRAP experiment of Fig 2A as an example.

When working with photosensitive probes, such as NBD or fluorescein-based molecules (*e*.*g*. CF and FITC), one has to correct for the intrinsic photobleaching that occurs during the recovery phase and can also affect data analysis [54,55]. This can be easily done in FRAPP experiments as shown in Fig 2C. In FRAP experiments, we measured the intrinsic photobleaching in a region far away from the bleaching zone (in the case of supported lipid bilayers or sponge phases) or on another giant liposome. The corrected fluorescence recovery signal was then calculated by dividing the raw fluorescence recovery signal by the intrinsic photobleaching signal.

### Optimization of FRAP and FRAPP data acquisition and fitting *in silico*

In this section, we want to optimize FRAP and FRAPP data acquisition and estimate what accuracy can be expected from the measurements. When performing FRAP(P) experiments, for any given sample and any given bleaching area, one should experimentally acquire a certain number of independent fluorescence recovery curves in order to extract, from these curves, an average characteristic diffusion time $\bar{\tau }$ with a low standard deviation. The way to acquire data, notably the acquisition time (*i*.*e*. the total duration of the recovery curve, *t*_*rec*_) and the acquisition frequency (*i*.*e*. the number of images recorded per second, *N / t*_*rec*_, where *N* is the total number of data points), will also affect the precision on $\bar{\tau }$. Our approach here is to simulate experiments in order to optimize data acquisition.

To perform such *in silico* simulations, we generated various theoretical fluorescence recovery curves. We chose to simulate experiments consisting of *N* = 60 data points, which provides sufficient confocal images to obtain a recovery curve without generating too much intrinsic bleaching. All curves were normalized between 0 and 1 (*i*.*e*. with the initial intensity *I*_*0*_ = 0 and the plateau intensity *I*_*∞*_ = 1) but displayed different characteristic diffusion times τ. For each τ value, we generated the corresponding ideal fluorescence recovery curve (without any noise) using Eq 1 in the case of FRAP or Eq 4 in the case of FRAPP. S1 Fig provides an example of such an ideal fluorescence recovery curve (red data points). To simulate actual experiments, a Gaussian noise was added to this ideal curve as a pragmatic approximation for the integration of the various noises that lead to experimental fluorescence values (laser, objective, photomultiplier, ambient light, *etc*.). This Gaussian distribution was dependent on the fluorescence intensity *I(i)*, where *i* is between 1 and 60, such that it was centered on 0 with the standard deviation k √*I(i)* (the standard deviation is proportional to the square root of *I(i)* to account for the fact that the noise is signal-dependent). The resulting signal-to-noise ratio, √*I(i)* / k, is therefore maximum at the plateau, where it is equal to 1 / *k*. Hence, for each simulated fluorescence recovery curve, the simulated intensity follows: *I*_*sim*_ *(i)* = *I(i)* + *G(i)*, where *G(i)* is a random number with the Gaussian probability distribution described above. We chose to vary the noise factor k from 0.02 to 0.5 based on typical variability of fluorescence intensity values observed in real FRAP(P) experiments. Several examples of *in silico* simulated fluorescence recovery curves are displayed in S1 and S2 Figs. The simulated fluorescence recovery curves were then approximated using Eqs 1 or 4, leading to fitted values of *I*_*0*_, *I*_*∞*_ and τ (S2 Fig). In order to assess the goodness of each fit, the code (written with Mathematica) also calculated:

$$E=\;\sqrt{\frac{1}{N}\sum_{i=1}^{N}{\left(I_{sim}\left(i\right)-I_{fit}\left(i\right)\right)}^{2}}$$ (10)

Where *I*_*fit*_ *(i)* is the fitted fluorescence value and N is the total number of simulated data points (E must therefore be close to 0).

The results of the different simulations we have performed for FRAP and FRAPP experiments with one or two diffusive species are presented in S3–S5 Figs.

## Supporting Information

S1 Fig Typical *in silico* simulated fluorescence recovery curve of a FRAP experiment (*i*.*e*. with the disk-shaped bleaching geometry). The simulated fluorescence intensity values (black diamonds) were generated by adding Gaussian noise *G(i)* to the theoretical values *I(i)* (red dots) deduced from Eq 1. *G(i)* is a random number with the Gaussian probability distribution centered on 0 with the standard deviation k √*I(i)* (zone delimited by the two dashed curves). In this example, τ = 3 frame periods and k = 0.05.
(PDF)

S2 Fig Examples of 3 fitted *in silico* simulated fluorescence recovery curves for FRAP experiments (*i*.*e*. with the disk-shaped bleaching geometry) with 3 different characteristic diffusion times: τ = 0.5 (red), 5 (blue) or 50 (green) frame periods. The noise factor k used to generate the simulated fluorescence intensity values was 0.05 in all 3 cases. The fits were obtained using Eq 1.
(PDF)

S3 Fig*In silico* study of FRAP and FRAPP experiments with a single diffusive species.Simulated experimental curves were generated from a normalized theoretical fluorescence recovery curve. These curves were composed of N = 60 intensity data points *I*_*sim*_ *(i)* = *I(i)* + *G(i)*, where the noise *G(i)* is a random number with the Gaussian probability distribution centered on 0 with the standard deviation k √*I(i)* (see methods and the examples in S1 and S2 Figs). The acquisition time was fixed at 60 frame periods. The noise factor k was varied from 0.02 to 0.5 and the characteristic diffusion time τ from 0.2 to 100 frame periods. For each condition, we generated 3000 simulated fluorescence recovery curves and we randomly selected some of these curves in order to form 1000 groups of 3 curves. For each group, the average characteristic diffusion time $\bar{\tau }$ was calculated. The average and the standard deviation on these $\bar{\tau }$, < $\bar{\tau }$ > and σ($\bar{\tau }$), were then calculated across the 1000 groups of 3 curves. The standard deviation σ($\bar{\tau }$) thus reflects the variability on $\bar{\tau }$ if the experimentalist were to reproduce 1000 times the same protocol, each time with 3 independent fluorescence recovery curves. The solvable cases (in green) were arbitrarily defined as the cases where < $\bar{\tau }$ > differs by less than 20% from the theoretical τ value and σ($\bar{\tau }$) / τ is lower than 30%. The numerical values of < $\bar{\tau }$ > and σ($\bar{\tau }$) are displayed in S1 Table. The graphs in (A) for FRAP and (B) for FRAPP show the range of τ values that can be accurately measured for any given set of parameters (k, frame rate), and thus allows the experimentalist to optimize the acquisition parameters as a function of the signal-to-noise ratio and the characteristic diffusion time τ. For example, in the case of a typical noise factor k equal to 0.1 (*i*.*e*. a signal-to-noise ratio of 10 at the plateau), FRAP experiments should be recorded at a frame rate of at least 2/τ, and the acquisition time should be between 6τ and 30τ.(PDF)

S4 Fig*In silico* study of FRAP experiments with two diffusive species.Simulated experimental curves were generated from a normalized theoretical fluorescence recovery curve as described in the legend of S3 Fig. The acquisition time was fixed at 60 frame periods. The noise factor k was varied from 0.001 to 0.01, the characteristic diffusion time τ_1_ from 0.2 to 50 frame periods, and the characteristic diffusion time τ_2_ from 0.5 to 100 frame periods (always keeping τ_2_ > τ_1_). The fraction of each species was also varied from 10 to 90% (in increments of 10%) and characterized by the fraction of the first species: R = *I*_*1∞*_ / (*I*_*1∞*_ + *I*_*2∞*_). For each condition, we generated 300 simulated fluorescence recovery curves and we randomly selected some of these curves in order to form 100 groups of 3 curves. For each group, we calculated the average characteristic diffusion times ${\bar{\tau }}_{1}$ and ${\bar{\tau }}_{2,}$ and the average fraction $\bar{\text{R}}$. The averages < ${\bar{\tau }}_{1}$ >, < ${\bar{\tau }}_{2}$ >, < $\bar{\text{R}}$ > and the standard deviations σ(${\bar{\tau }}_{1}$), σ(${\bar{\tau }}_{2}$), σ($\bar{\text{R}}$) on these ${\bar{\tau }}_{1}$, ${\bar{\tau }}_{2}$ and $\bar{\text{R}}$ were then calculated across the 100 groups of 3 curves. These standard deviations thus reflect the variability on ${\bar{\tau }}_{1}$, ${\bar{\tau }}_{2}$ and R if the experimentalist were to reproduce 100 times the same protocol, each time with 3 independent fluorescence recovery curves. The solvable cases (in green) were arbitrarily defined as the cases where < ${\bar{\tau }}_{1}$ >, < ${\bar{\tau }}_{2}$ > and < $\bar{\text{R}}$ > all differ by less than 20% from the theoretical values, and all standard deviations are lower than 20%. The numerical values of < ${\bar{\tau }}_{1}$ >, < ${\bar{\tau }}_{2}$ >, < $\bar{\text{R}}$ >, σ(${\bar{\tau }}_{1}$), σ(${\bar{\tau }}_{2}$) and σ($\bar{\text{R}}$) are displayed in S2 Table. We show here only the results obtained for R = 0.5 and a noise factor k equal to 0.001 (A), 0.002 (B), 0.005 (C) or 0.01 (D). Compared to the case of a single diffusive species, the signal-to-noise ratio has to be very high in order to obtain accurate values of both τ_1_ and τ_2_. In the case of a noise factor k equal to 0.001 (*i*.*e*. a signal-to-noise ratio of 1000 at the plateau), and assuming that τ_1_ and τ_2_ are already in the measurable range permitted by the acquisition parameters, the experimentalist should be able to distinguish two species by FRAP and to accurately measure their characteristic diffusion time if τ_2_ is at least 5 times larger than τ_1_ (panel A).(PDF)

S5 Fig*In silico* study of FRAPP experiments with two diffusive species.Simulated experimental curves were generated from a normalized theoretical fluorescence recovery curve as described in the legend of S3 Fig. The acquisition time was fixed at 60 frame periods. The noise factor k was varied from 0.001 to 0.05, the characteristic diffusion time τ_1_ from 0.2 to 50 frame periods, and the characteristic diffusion time τ_2_ from 0.5 to 100 frame periods (always keeping τ_2_ > τ_1_). The fraction of each species was also varied from 10 to 90% (in increments of 10%) and characterized by the fraction of the first species: R = *I*_*1∞*_ / (*I*_*1∞*_ + *I*_*2∞*_). For each condition, we generated 300 simulated fluorescence recovery curves and we randomly selected some of these curves in order to form 100 groups of 3 curves. For each group, we calculated the average characteristic diffusion times ${\bar{\tau }}_{1}$ and ${\bar{\tau }}_{2,}$ and the average fraction $\bar{\text{R}}$. The averages < ${\bar{\tau }}_{1}$ >, < ${\bar{\tau }}_{2}$ >, < $\bar{\text{R}}$ > and the standard deviations σ(${\bar{\tau }}_{1}$), (${\bar{\tau }}_{2}$), σ($\bar{\text{R}}$) on these ${\bar{\tau }}_{1}$, ${\bar{\tau }}_{2}$ and $\bar{\text{R}}$ were then calculated across the 100 groups of 3 curves. These standard deviations thus reflect the variability on ${\bar{\tau }}_{1}$, ${\bar{\tau }}_{2}$ and R if the experimentalist were to reproduce 100 times the same protocol, each time with 3 independent fluorescence recovery curves. The solvable cases (in green) were arbitrarily defined as the cases where < ${\bar{\tau }}_{1}$ >, < ${\bar{\tau }}_{2}$ > and < $\bar{\text{R}}$ > all differ by less than 20% from the theoretical values, and all standard deviations are lower than 20%. The numerical values of < ${\bar{\tau }}_{1}$ >, < ${\bar{\tau }}_{2}$ >, < $\bar{\text{R}}$ >, σ(${\bar{\tau }}_{1}$), σ(${\bar{\tau }}_{2}$) and σ($\bar{\text{R}}$) are displayed in S3 Table. We show here only the results obtained for R = 0.5 and a noise factor k equal to 0.001 (A), 0.002 (B), 0.005 (C), 0.01 (D), 0.02 (E) and 0.05 (F). Compared to the case of a single diffusive species, the signal-to-noise ratio has to be very high in order to obtain accurate values of both τ_1_ and τ_2_. In the case of a noise factor k equal to 0.001 (*i*.*e*. a signal-to-noise ratio of 1000 at the plateau), and assuming that τ_1_ and τ_2_ are already in the measurable range permitted by the acquisition parameters, the experimentalist should be able to distinguish two species by FRAPP and to accurately measure their characteristic diffusion time if τ_2_ is at least 2 times larger than τ_1_ (panel A).(PDF)

S1 TableNumerical values of the *in silico* studies of FRAP and FRAPP experiments with a single diffusing species (see the legend of S3 Fig for details).Each cell displays, for a given pair of (τ, k) values, (i) the normalized difference between < $\bar{\tau }$ > and τ, (< $\bar{\tau }$ >—τ) / τ (left), and (ii) the normalized standard deviation, σ($\bar{\tau }$) / τ (right). The solvable cases (in green) were arbitrarily defined as the cases where < $\bar{\tau }$ > differs by less than 20% from τ and σ($\bar{\tau }$) / τ is lower than 30%.(PDF)

S2 TableNumerical values of the *in silico* studies of FRAP experiments with two diffusing species (see the legend of S4 Fig for details).Each cell displays, for a given pair of (τ_1_, τ_2_) values, (i) the normalized differences between the averages and the theoretical values (left): (< ${\bar{\tau }}_{1}$ >—τ_1_) / τ_1_ (first line), (< ${\bar{\tau }}_{2}$ >—τ_2_) / τ_2_ (second line), │< $\bar{\text{R}}$ >—R│ / R + │(1 - < $\bar{\text{R}}$ >)—(1—R)│ / (1 –R) (third line), and (ii) the associated normalized standard deviations (right): σ(${\bar{\tau }}_{1}$) / τ_1_ (first line), σ(${\bar{\tau }}_{2}$) / τ_2_ (second line) and σ($\bar{\text{R}}$) / R (third line). The solvable cases (in green) were arbitrarily defined as the cases where < ${\bar{\tau }}_{1}$ >, < ${\bar{\tau }}_{2}$ > and < $\bar{\text{R}}$ > all differ by less than 20% from the theoretical values, and all normalized standard deviations are lower than 20%.(PDF)

S3 TableNumerical values of the *in silico* studies of FRAPP experiments with two diffusing species (see the legend of S5 Fig for details).Each cell displays, for a given pair of (τ_1_, τ_2_) values, (i) the normalized differences between the averages and the theoretical values (left): (< ${\bar{\tau }}_{1}$ >—τ_1_) / τ_1_ (first line), (< ${\bar{\tau }}_{2}$ >—τ_2_) / τ_2_ (second line), │< $\bar{\text{R}}$ >—R│ / R + │(1 - < $\bar{\text{R}}$ >)—(1—R)│ / (1 –R) (third line), and (ii) the associated normalized standard deviations (right): σ(${\bar{\tau }}_{1}$) / τ_1_ (first line), σ(${\bar{\tau }}_{2}$) / τ_2_ (second line) and σ($\bar{\text{R}}$) / R (third line). The solvable cases (in green) were arbitrarily defined as the cases where < ${\bar{\tau }}_{1}$ >, < ${\bar{\tau }}_{2}$ > and < $\bar{\text{R}}$ > all differ by less than 20% from the theoretical values, and all normalized standard deviations are lower than 20%.(PDF)

S1 Text Experimental determination of the time t0 of the recovery phase. (PDF)
