# Supplementary material for: FRAP to Characterize Molecular Diffusion and Interaction in Various Membrane Environments
Source: PLoS One. 2016 Jul 7;11(7):e0158457. doi: 10.1371/journal.pone.0158457 (PMC4936743; doi:10.1371/journal.pone.0158457)
Supplement: S1 Fig — The simulated fluorescence intensity values (black diamonds) were generated by adding Gaussian noise G(i) to the theoretical values I(i) (red dots) deduced from Eq 1. G(i) is a random number with the Gaussian probability distribution centered on 0 with the standard deviation k √I(i) (zone delimited by the two dashed curves). In this example, τ = 3 frame periods and k = 0.05. (PDF) [file pone.0158457.s001.pdf]

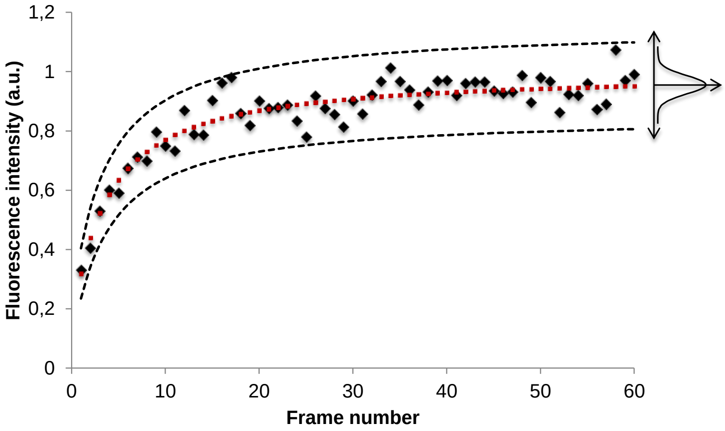

S1 Figure. Typical *in silico* simulated fluorescence recovery curve of a FRAP experiment (*i.e.* with the disk-shaped bleaching geometry). The simulated fluorescence intensity values (black diamonds) were generated by adding Gaussian noise *G(i)* to the theoretical values *I(i)* (red dots) deduced from Equation (1). *G(i)* is a random number with the Gaussian probability distribution centered on 0 with the standard deviation k $\sqrt{I(i)}$ (zone delimited by the two dashed curves). In this example, τ = 3 frame periods and k = 0.05.
