# Supplementary material for: FRAP to Characterize Molecular Diffusion and Interaction in Various Membrane Environments
Source: PLoS One. 2016 Jul 7;11(7):e0158457. doi: 10.1371/journal.pone.0158457 (PMC4936743; doi:10.1371/journal.pone.0158457)
Supplement: S2 Fig — The noise factor k used to generate the simulated fluorescence intensity values was 0.05 in all 3 cases. The fits were obtained using Eq 1. (PDF) [file pone.0158457.s002.pdf]

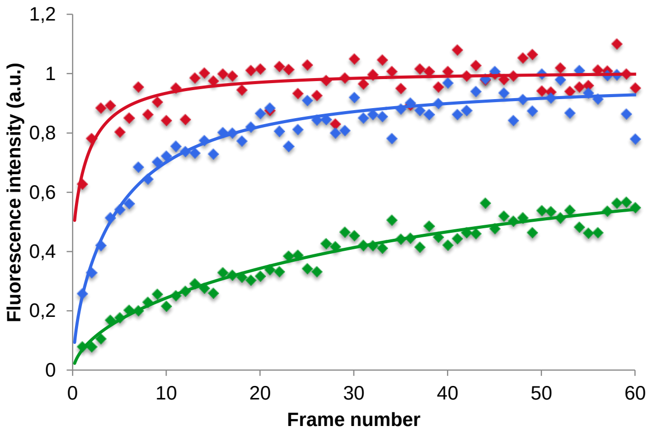

S2 Figure. Examples of 3 fitted *in silico* simulated fluorescence recovery curves for FRAP experiments (*i.e.* with the disk-shaped bleaching geometry) with 3 different characteristic diffusion times: τ = 0.5 (red), 5 (blue) or 50 (green) frame periods. The noise factor k used to generate the simulated fluorescence intensity values was 0.05 in all 3 cases. The fits were obtained using Equation (1).
