# Supplementary material for: FRAP to Characterize Molecular Diffusion and Interaction in Various Membrane Environments
Source: PLoS One. 2016 Jul 7;11(7):e0158457. doi: 10.1371/journal.pone.0158457 (PMC4936743; doi:10.1371/journal.pone.0158457)
Supplement: S3 Fig — Simulated experimental curves were generated from a normalized theoretical fluorescence recovery curve. These curves were composed of N = 60 intensity data points Isim (i) = I(i) + G(i), where the noise G(i) is a random number with the Gaussian probability distribution centered on 0 with the standard deviation k √I(i) (see methods and the examples in S1 and S2 Figs). The acquisition time was fixed at 60 frame periods. The noise factor k was varied from 0.02 to 0.5 and the characteristic diffusion time τ from 0.2 to 100 frame periods. For each condition, we generated 3000 simulated fluorescence recovery curves and we randomly selected some of these curves in order to form 1000 groups of 3 curves. For each group, the average characteristic diffusion time τ¯ was calculated. The average and the standard deviation on these τ¯, < τ¯ > and σ(τ¯), were then calculated across the 1000 groups of 3 curves. The standard deviation σ(τ¯) thus reflects the variability on τ¯ if the experimentalist were to reproduce 1000 times the same protocol, each time with 3 independent fluorescence recovery curves. The solvable cases (in green) were arbitrarily defined as the cases where < τ¯ > differs by less than 20% from the theoretical τ value and σ(τ¯) / τ is lower than 30%. The numerical values of < τ¯ > and σ(τ¯) are displayed in S1 Table. The graphs in (A) for FRAP and (B) for FRAPP show the range of τ values that can be accurately measured for any given set of parameters (k, frame rate), and thus allows the experimentalist to optimize the acquisition parameters as a function of the signal-to-noise ratio and the characteristic diffusion time τ. For example, in the case of a typical noise factor k equal to 0.1 (i.e. a signal-to-noise ratio of 10 at the plateau), FRAP experiments should be recorded at a frame rate of at least 2/τ, and the acquisition time should be between 6τ and 30τ. (PDF) [file pone.0158457.s003.pdf]

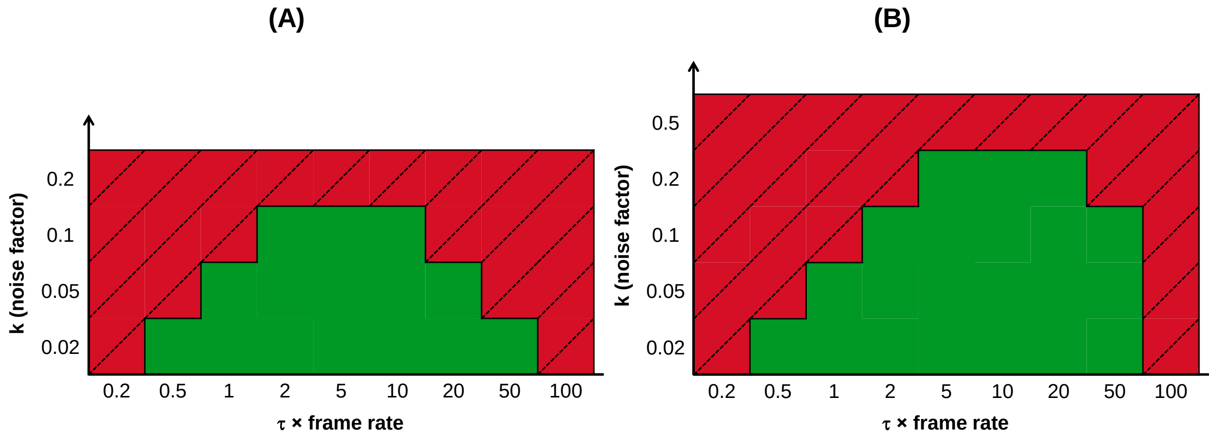

S3 Figure. *In silico* study of FRAP and FRAPP experiments with a single diffusive species. Simulated experimental curves were generated from a normalized theoretical fluorescence recovery curve. These curves were composed of N = 60 intensity data points $I_{sim}(i) = I(i) + G(i)$, where the noise $G(i)$ is a random number with the Gaussian probability distribution centered on 0 with the standard deviation k $\sqrt{I(i)}$ (see methods and the examples in S1 and S2 Figs). The acquisition time was fixed at 60 frame periods. The noise factor k was varied from 0.02 to 0.5 and the characteristic diffusion time τ from 0.2 to 100 frame periods. For each condition, we generated 3000 simulated fluorescence recovery curves and we randomly selected some of these curves in order to form 1000 groups of 3 curves. For each group, the average characteristic diffusion time $\bar{\tau}$ was calculated. The average and the standard deviation on these $\bar{\tau}$, $<\bar{\tau}>$ and $\sigma(\bar{\tau})$, were then calculated across the 1000 groups of 3 curves. The standard deviation $\sigma(\bar{\tau})$ thus reflects the variability on $\bar{\tau}$ if the experimentalist were to reproduce 1000 times the same protocol, each time with 3 independent fluorescence recovery curves. The solvable cases (in green) were arbitrarily defined as the cases where $<\bar{\tau}>$ differs by less than 20% from the theoretical τ value and $\sigma(\bar{\tau}) / \tau$ is lower than 30%. The numerical values of $<\bar{\tau}>$ and $\sigma(\bar{\tau})$ are displayed in S1 Table. The graphs in (A) for FRAP and (B) for FRAPP show the range of τ values that can be accurately measured for any given set of parameters (k, frame rate), and thus allows the experimentalist to optimize the acquisition parameters as a function of the signal-to-noise ratio and the characteristic diffusion time τ. For example, in the case of a typical noise factor k equal to 0.1 (*i.e.* a signal-to-noise ratio of 10 at the plateau), FRAP experiments should be recorded at a frame rate of at least $2/\tau$, and the acquisition time should be between 6τ and 30τ.
