# Supplementary material for: FRAP to Characterize Molecular Diffusion and Interaction in Various Membrane Environments
Source: PLoS One. 2016 Jul 7;11(7):e0158457. doi: 10.1371/journal.pone.0158457 (PMC4936743; doi:10.1371/journal.pone.0158457)
Supplement: S4 Fig — Simulated experimental curves were generated from a normalized theoretical fluorescence recovery curve as described in the legend of S3 Fig. The acquisition time was fixed at 60 frame periods. The noise factor k was varied from 0.001 to 0.01, the characteristic diffusion time τ1 from 0.2 to 50 frame periods, and the characteristic diffusion time τ2 from 0.5 to 100 frame periods (always keeping τ2 > τ1). The fraction of each species was also varied from 10 to 90% (in increments of 10%) and characterized by the fraction of the first species: R = I1∞ / (I1∞ + I2∞). For each condition, we generated 300 simulated fluorescence recovery curves and we randomly selected some of these curves in order to form 100 groups of 3 curves. For each group, we calculated the average characteristic diffusion times τ¯1 and τ¯2, and the average fraction R¯. The averages < τ¯1 >, < τ¯2 >, < R¯ > and the standard deviations σ(τ¯1), σ(τ¯2), σ(R¯) on these τ¯1, τ¯2 and R¯ were then calculated across the 100 groups of 3 curves. These standard deviations thus reflect the variability on τ¯1, τ¯2 and R if the experimentalist were to reproduce 100 times the same protocol, each time with 3 independent fluorescence recovery curves. The solvable cases (in green) were arbitrarily defined as the cases where < τ¯1 >, < τ¯2 > and < R¯ > all differ by less than 20% from the theoretical values, and all standard deviations are lower than 20%. The numerical values of < τ¯1 >, < τ¯2 >, < R¯ >, σ(τ¯1), σ(τ¯2) and σ(R¯) are displayed in S2 Table. We show here only the results obtained for R = 0.5 and a noise factor k equal to 0.001 (A), 0.002 (B), 0.005 (C) or 0.01 (D). Compared to the case of a single diffusive species, the signal-to-noise ratio has to be very high in order to obtain accurate values of both τ1 and τ2. In the case of a noise factor k equal to 0.001 (i.e. a signal-to-noise ratio of 1000 at the plateau), and assuming that τ1 and τ2 are already in the measurable range permitted by the acquisition [file pone.0158457.s004.pdf]

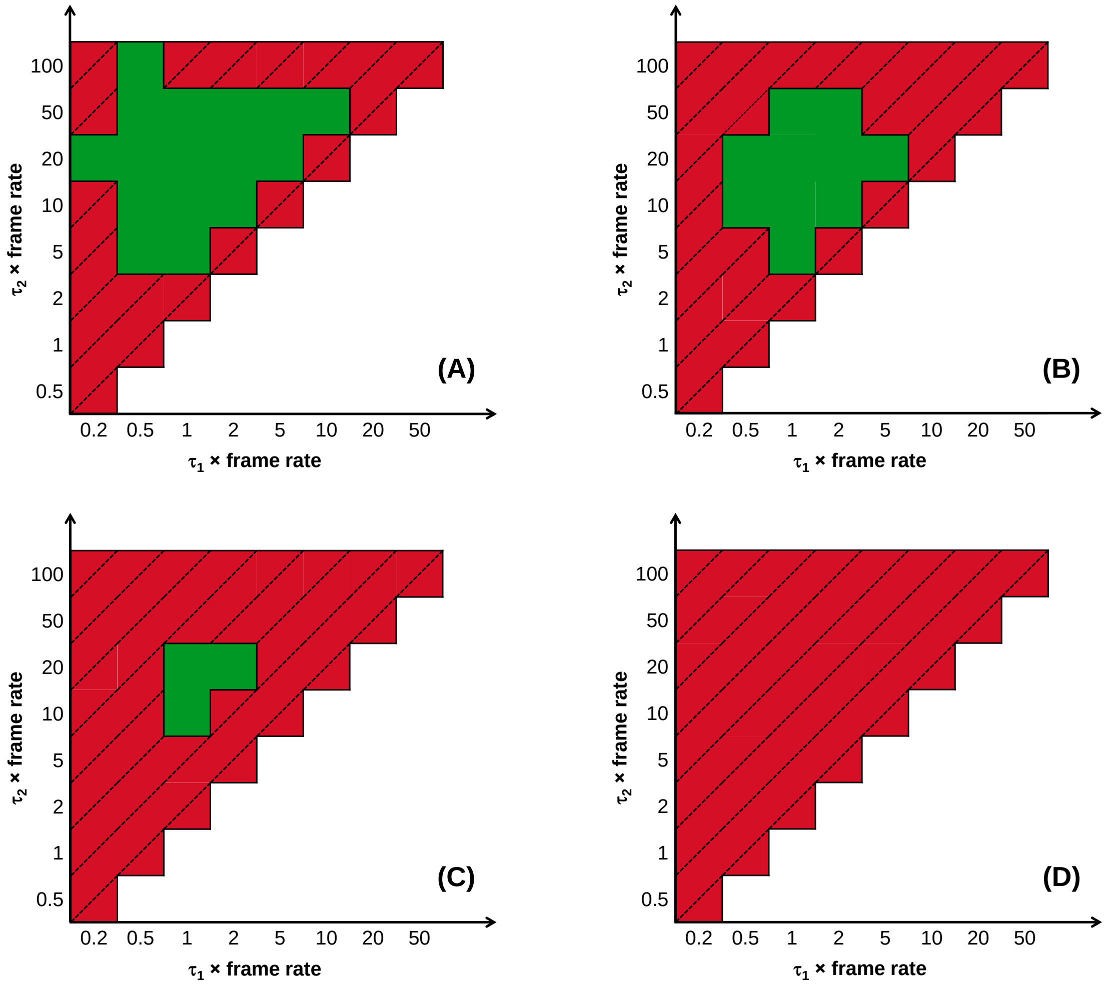

S4 Figure. *In silico* study of FRAP experiments with two diffusive species. Simulated experimental curves were generated from a normalized theoretical fluorescence recovery curve as described in the legend of S3 Fig. The acquisition time was fixed at 60 frame periods. The noise factor k was varied from 0.001 to 0.01, the characteristic diffusion time τ_1 from 0.2 to 50 frame periods, and the characteristic diffusion time τ_2 from 0.5 to 100 frame periods (always keeping $\tau_2 > \tau_1$). The fraction of each species was also varied from 10 to 90% (in increments of 10%) and characterized by the fraction of the first species: $R = I_{1\infty} / (I_{1\infty} + I_{2\infty})$. For each condition, we generated 300 simulated fluorescence recovery curves and we randomly selected some of these curves in order to form 100 groups of 3 curves. For each group, we calculated the average characteristic diffusion times $\bar{\tau}_1$ and $\bar{\tau}_2$, and the average fraction $\bar{R}$. The averages $< \bar{\tau}_1 >$, $< \bar{\tau}_2 >$, $< \bar{R} >$ and the standard deviations $\sigma(\bar{\tau}_1)$, $\sigma(\bar{\tau}_2)$, $\sigma(\bar{R})$ on these $\bar{\tau}_1$, $\bar{\tau}_2$ and $\bar{R}$ were then calculated across the 100 groups of 3 curves. These standard deviations thus reflect the variability on $\bar{\tau}_1$, $\bar{\tau}_2$ and R if the experimentalist were to reproduce 100 times the same protocol, each time with 3 independent fluorescence recovery curves. The solvable cases (in green) were arbitrarily defined as the cases where $< \bar{\tau}_1 >$, $< \bar{\tau}_2 >$ and $< \bar{R} >$ all differ by less

than 20% from the theoretical values, and all standard deviations are lower than 20%. The numerical values of $<\bar{\tau}_1>$, $<\bar{\tau}_2>$, $<\bar{R}>$, $\sigma(\bar{\tau}_1)$, $\sigma(\bar{\tau}_2)$ and $\sigma(\bar{R})$ are displayed in S2 Table. We show here only the results obtained for R = 0.5 and a noise factor k equal to 0.001 (A), 0.002 (B), 0.005 (C) or 0.01 (D). Compared to the case of a single diffusive species, the signal-to-noise ratio has to be very high in order to obtain accurate values of both τ_1 and τ_2. In the case of a noise factor k equal to 0.001 (*i.e.* a signal-to-noise ratio of 1000 at the plateau), and assuming that τ_1 and τ_2 are already in the measurable range permitted by the acquisition parameters, the experimentalist should be able to distinguish two species by FRAP and to accurately measure their characteristic diffusion time if τ_2 is at least 5 times larger than τ_1 (panel A).
