# Supplementary material for: FRAP to Characterize Molecular Diffusion and Interaction in Various Membrane Environments
Source: PLoS One. 2016 Jul 7;11(7):e0158457. doi: 10.1371/journal.pone.0158457 (PMC4936743; doi:10.1371/journal.pone.0158457)
Supplement: S1 Table — Each cell displays, for a given pair of (τ, k) values, (i) the normalized difference between < τ¯ > and τ, (< τ¯ >—τ) / τ (left), and (ii) the normalized standard deviation, σ(τ¯) / τ (right). The solvable cases (in green) were arbitrarily defined as the cases where < τ¯ > differs by less than 20% from τ and σ(τ¯) / τ is lower than 30%. (PDF) [file pone.0158457.s006.pdf]

S1 Table. Numerical values of the *in silico* studies of FRAP and FRAPP experiments with a single diffusing species (see the legend of S3 Fig for details). Each cell displays, for a given pair of (τ, k) values, (i) the normalized difference between $<\bar{\tau}>$ and τ, $(<\bar{\tau}> - \tau) / \tau$ (left), and (ii) the normalized standard deviation, $\sigma(\bar{\tau}) / \tau$ (right). The solvable cases (in green) were arbitrarily defined as the cases where $<\bar{\tau}>$ differs by less than 20% from τ and $\sigma(\bar{\tau}) / \tau$ is lower than 30%.

FRAP

τ × frame rate

Noise factor k	0,2		0,5		1		2		5		10		20		50		100	
0,02	0,50	0,96	0,02	0,24	0,01	0,10	0,00	0,05	0,00	0,03	0,00	0,03	0,00	0,04	0,07	0,18	4,30	7,30
0,05	1190,00	10590,00	0,21	0,66	0,05	0,25	0,02	0,13	0,01	0,09	0,02	0,09	0,04	0,16	9,00	21,00	23,00	25,00
0,1			453,00	3769,00	0,33	2,03	0,10	0,33	0,05	0,20	0,07	0,26	4,10	27,00	56,00	70,00		
0,2			11219,00	30928,00	832,00	4135,00	172,00	1103,00	21,00	148,00	27,00	122,00	95,00	190,00	161,00	159,00		

FRAPP

τ × frame rate

Noise factor k	0,2		0,5		1		2		5		10		20		50		100	
0,02	8,66	109,04	0,02	0,34	0,01	0,07	0,00	0,03	0,00	0,02	0,00	0,01	0,00	0,01	0,00	0,03	-1,00	0,00
0,05	5,46	55,98	0,58	3,03	0,04	0,17	0,01	0,08	0,00	0,04	0,00	0,03	0,01	0,04	0,02	0,09	-0,99	0,10
0,1	6,30	35,53	1,95	13,27	0,19	0,62	0,02	0,18	0,01	0,08	-0,04	0,13	0,01	0,08	0,12	0,28	-0,81	2,22
0,2	8,46	50,84	4,58	39,71	1,12	8,45	0,16	0,55	0,02	0,20	-0,12	0,25	0,03	0,24	1,58	19,55	-0,37	7,48
0,5	11,40	59,78	14,52	241,81	3,46	36,84	1,51	14,01	0,83	12,37	0,07	1,29	0,41	3,01	2,29	31,81	-0,60	2,00
