# Supplementary material for: FRAP to Characterize Molecular Diffusion and Interaction in Various Membrane Environments
Source: PLoS One. 2016 Jul 7;11(7):e0158457. doi: 10.1371/journal.pone.0158457 (PMC4936743; doi:10.1371/journal.pone.0158457)
Supplement: S2 Table — Each cell displays, for a given pair of (τ1, τ2) values, (i) the normalized differences between the averages and the theoretical values (left): (< τ¯1 >—τ1) / τ1 (first line), (< τ¯2 >—τ2) / τ2 (second line), │< R¯ >—R│ / R + │(1 - < R¯ >)—(1—R)│ / (1 –R) (third line), and (ii) the associated normalized standard deviations (right): σ(τ¯1) / τ1 (first line), σ(τ¯2) / τ2 (second line) and σ(R¯) / R (third line). The solvable cases (in green) were arbitrarily defined as the cases where < τ¯1 >, < τ¯2 > and < R¯ > all differ by less than 20% from the theoretical values, and all normalized standard deviations are lower than 20%. (PDF) [file pone.0158457.s007.pdf]

S2 Table. Numerical values of the *in silico* studies of FRAP experiments with two diffusing species (see the legend of S4 Fig for details). Each cell displays, for a given pair of (τ_1, τ_2) values, (i) the normalized differences between the averages and the theoretical values (left): ($< \bar{\tau}_1 > - \tau_1$) / τ_1 (first line), ($< \bar{\tau}_2 > - \tau_2$) / τ_2 (second line), $| < \bar{R} > - R |$ / R + $|(1 - < \bar{R} >) - (1 - R)|$ / (1 – R) (third line), and (ii) the associated normalized standard deviations (right): $\sigma(\bar{\tau}_1)$ / τ_1 (first line), $\sigma(\bar{\tau}_2)$ / τ_2 (second line) and $\sigma(\bar{R})$ / R (third line). The solvable cases (in green) were arbitrarily defined as the cases where $< \bar{\tau}_1 >$, $< \bar{\tau}_2 >$ and $< \bar{R} >$ all differ by less than 20% from the theoretical values, and all normalized standard deviations are lower than 20%.

R=0.1

τ_1 × frame rate

τ_2 × frame rate

k = 0,001	0,2		0,5		1		2		5		10		20		50	
0,5	1,12<br>3156,03<br>10,55	0,56<br>5991,37<br>3,05														
1	2,90<br>5,43<br>8,64	0,97<br>20,06<br>3,36	0,63<br>5,35<br>9,27	0,34<br>20,38<br>2,95												
2	2,61<br>2,24<br>3,35	2,27<br>13,13<br>1,92	0,56<br>0,94<br>4,07	0,84<br>3,05<br>1,86	-0,05<br>0,72<br>3,93	0,49<br>1,37<br>2,32										
5	1,99<br>0,03<br>1,06	2,47<br>0,07<br>0,76	0,15<br>0,01<br>0,76	0,52<br>0,01<br>0,62	0,01<br>0,01<br>0,57	0,41<br>0,04<br>0,60	0,09<br>0,15<br>2,11	0,43<br>0,21<br>1,88								
10	0,60<br>0,00<br>0,63	0,96<br>0,01<br>0,42	0,05<br>0,00<br>0,28	0,31<br>0,01<br>0,36	0,05<br>0,00<br>0,06	0,18<br>0,01<br>0,06	0,00<br>0,00<br>0,11	0,15<br>0,01<br>0,09	-0,02<br>0,13<br>2,11	0,28<br>0,14<br>1,59						
20	0,27<br>0,00<br>0,49	0,60<br>0,01<br>0,36	-0,02<br>0,00<br>0,11	0,19<br>0,01<br>0,12	0,02<br>0,00<br>0,01	0,08<br>0,01<br>0,01	0,01<br>0,00<br>0,04	0,07<br>0,01<br>0,02	0,02<br>0,01<br>0,19	0,14<br>0,02<br>0,15	0,00<br>0,14<br>2,11	0,29<br>0,15<br>1,56				
50	0,22<br>0,00<br>0,26	0,42<br>0,01<br>0,20	0,01<br>0,00<br>0,07	0,14<br>0,01<br>0,06	0,00<br>0,00<br>0,01	0,07<br>0,02<br>0,01	0,00<br>0,00<br>0,01	0,05<br>0,02<br>0,01	0,00<br>0,00<br>0,05	0,07<br>0,04<br>0,03	0,01<br>0,04<br>0,19	0,13<br>0,10<br>0,16	0,01<br>0,16<br>1,04	0,25<br>0,16<br>0,73		
100	213,08<br>-0,26<br>1,20	41,02<br>0,03<br>2,03	-0,74<br>-0,24<br>1,75	0,10<br>0,02<br>0,41	0,00<br>0,01<br>0,02	0,05<br>0,05<br>0,01	0,00<br>0,02<br>0,01	0,04<br>0,07<br>0,01	0,00<br>0,09<br>0,02	0,05<br>0,18<br>0,02	0,01<br>0,16<br>0,07	0,09<br>0,22<br>0,06	0,61<br>-0,28<br>0,55	0,27<br>0,03<br>0,32	0,23<br>-0,34<br>29,69	0,15<br>0,07<br>7,95

τ_2 × frame rate

k=0,002	0,2		0,5		1		2		5		10		20		50	
0,5	1,07<br>3045,59<br>9,23	0,87<br>8451,79<br>3,90														
1	3,30<br>3368,85<br>9,64	0,96<br>4767,18<br>2,13	0,64<br>3621,61<br>9,81	0,60<br>4372,65<br>2,05												
2	5,36<br>1218,98<br>6,99	2,66<br>1611,70<br>2,71	1,73<br>942,90<br>6,62	0,77<br>1758,29<br>2,44	0,57<br>1575,36<br>8,10	0,36<br>1898,37<br>2,28										
5	5,83<br>49,77<br>2,27	4,92<br>122,88<br>1,76	0,74<br>13,46<br>1,58	1,08<br>54,83<br>1,05	0,32<br>6,87<br>1,81	0,76<br>29,30<br>1,41	0,14<br>66,95<br>3,41	0,49<br>152,80<br>2,35								
10	4,97<br>1,31<br>1,26	6,67<br>8,29<br>1,18	0,20<br>0,00<br>0,67	0,58<br>0,01<br>0,68	0,06<br>0,01<br>0,29	0,40<br>0,02<br>0,34	0,07<br>0,02<br>0,47	0,30<br>0,04<br>0,67	0,18<br>35,72<br>3,70	0,36<br>58,38<br>2,40						
20	1,99<br>0,01<br>0,91	5,57<br>0,01<br>0,61	0,01<br>0,00<br>0,52	0,37<br>0,01<br>0,56	0,02<br>0,00<br>0,21	0,20<br>0,01<br>0,86	0,03<br>0,00<br>0,06	0,15<br>0,02<br>0,06	0,47<br>0,08<br>1,03	0,53<br>0,36<br>0,95	0,15<br>5,26<br>2,67	0,39<br>11,47<br>1,96				
50	0,56<br>0,01<br>0,77	0,87<br>0,02<br>0,52	-0,02<br>0,01<br>0,56	0,31<br>0,03<br>1,01	0,00<br>0,00<br>0,31	0,15<br>0,03<br>1,24	-0,01<br>0,01<br>0,72	0,14<br>0,04<br>1,92	-0,04<br>0,00<br>0,68	0,17<br>0,08<br>1,62	0,03<br>0,35<br>0,38	0,23<br>0,49<br>0,29	-0,05<br>0,97<br>1,39	0,33<br>1,88<br>1,19		
100	2,06<br>0,07<br>0,81	15,38<br>0,10<br>1,25	0,01<br>0,04<br>0,72	0,26<br>0,10<br>1,43	-0,18<br>-0,03<br>3,05	0,26<br>0,13<br>3,47	-0,05<br>0,07<br>1,12	0,16<br>0,28<br>2,71	0,01<br>0,41<br>0,07	0,10<br>0,73<br>0,05	-0,01<br>0,46<br>0,15	0,13<br>0,43<br>0,26	-0,13<br>0,32<br>0,67	0,28<br>0,42<br>0,86	-0,40<br>0,28<br>3,62	0,18<br>0,51<br>2,89

τ_2 × frame rate

k=0,005	0,2		0,5		1		2		5		10		20		50	
0,5	1,55<br>1936,44<br>15,69	0,89<br>3367,52<br>6,21														
1	2,61<br>241,62<br>11,31	1,34<br>479,63<br>5,99	0,53<br>234,20<br>11,56	0,60<br>480,70<br>5,67												
2	3,56<br>103,80<br>6,05	2,12<br>253,76<br>5,01	0,75<br>108,47<br>6,24	0,93<br>228,33<br>4,60	-0,03<br>115,11<br>6,27	0,45<br>232,56<br>4,83										
5	7,88<br>11,60<br>3,77	5,17<br>24,63<br>2,64	2,20<br>12,05<br>4,12	2,12<br>27,82<br>1,72	0,95<br>7,47<br>4,38	0,93<br>22,57<br>1,83	0,13<br>11,48<br>4,36	0,54<br>27,63<br>3,26								
10	11,31<br>1,59<br>2,87	8,27<br>4,55<br>2,36	2,21<br>1,02<br>2,24	2,58<br>2,95<br>1,26	0,71<br>0,95<br>1,68	1,22<br>3,45<br>1,25	0,42<br>3,11<br>2,02	0,70<br>7,89<br>1,55	0,06<br>2,46<br>4,40	0,37<br>4,86<br>3,02						
20	12,95<br>0,35<br>1,91	14,17<br>0,80<br>2,90	0,86<br>0,07<br>1,12	1,86<br>0,26<br>0,84	0,27<br>0,03<br>0,55	0,73<br>0,08<br>0,55	0,20<br>0,10<br>0,44	0,56<br>0,28<br>0,50	0,31<br>0,86<br>1,88	0,55<br>1,38<br>1,56	0,06<br>0,82<br>3,39	0,33<br>0,96<br>3,18				
50	20,01<br>0,20<br>2,60	25,97<br>0,46<br>4,37	0,34<br>0,07<br>0,70	0,79<br>0,20<br>0,56	0,10<br>0,06<br>0,22	0,36<br>0,15<br>0,31	0,07<br>0,15<br>0,06	0,26<br>0,49<br>0,05	0,06<br>0,69<br>0,20	0,31<br>1,38<br>0,14	0,08<br>0,82<br>0,84	0,36<br>0,81<br>1,29	0,08<br>2,28<br>2,83	0,38<br>3,32<br>3,37		
100	181,52<br>-0,24<br>18,33	73,58<br>0,18<br>12,80	0,30<br>-0,08<br>1,26	3,60<br>0,27<br>0,68	-0,02<br>0,46<br>0,38	0,33<br>0,83<br>0,50	-0,01<br>0,72<br>0,10	0,20<br>1,21<br>0,09	-0,04<br>0,97<br>0,16	0,16<br>1,18<br>0,10	-0,08<br>0,38<br>0,30	0,31<br>0,48<br>0,40	0,12<br>0,84<br>2,07	0,46<br>1,03<br>2,10	-0,40<br>0,82<br>5,47	0,22<br>0,97<br>3,87

τ_2 × frame rate

k=0,01	0,2		0,5		1		2		5		10		20		50	
0,5	1,83<br>1565,12<br>19,49	1,46<br>2770,40<br>10,19														
1	2,46<br>508,06<br>13,96	1,63<br>825,94<br>9,28	0,45<br>421,22<br>14,85	0,61<br>791,48<br>9,27												
2	3,61<br>206,02<br>16,47	1,90<br>359,33<br>12,57	0,56<br>146,51<br>12,75	0,70<br>270,39<br>10,96	-0,12<br>150,25<br>13,08	0,43<br>267,97<br>11,35										
5	8,98<br>31,83<br>6,81	5,78<br>56,84<br>5,72	2,84<br>30,69<br>7,61	1,99<br>54,59<br>6,64	1,10<br>35,77<br>5,81	0,97<br>57,85<br>4,11	0,16<br>33,03<br>6,91	0,51<br>62,44<br>6,47								
10	18,02<br>5,30<br>6,70	9,37<br>10,67<br>5,91	4,57<br>6,33<br>4,46	3,34<br>13,59<br>3,43	1,80<br>7,55<br>3,37	1,60<br>13,53<br>1,98	0,66<br>9,34<br>4,10	0,90<br>17,32<br>3,55	-0,02<br>6,96<br>7,73	0,36<br>13,62<br>7,00						
20	30,79<br>2,68<br>5,87	15,82<br>4,80<br>5,29	5,81<br>2,90<br>2,43	5,41<br>5,79<br>2,58	1,46<br>1,52<br>1,98	2,07<br>3,67<br>1,86	0,71<br>1,91<br>1,47	1,02<br>4,60<br>1,71	0,46<br>3,24<br>3,76	0,61<br>5,07<br>4,21	0,01<br>2,43<br>8,44	0,29<br>3,92<br>7,11				
50	55,09<br>1,64<br>9,68	36,55<br>2,70<br>11,45	4,65<br>1,01<br>3,06	6,85<br>2,49<br>5,01	0,58<br>1,69<br>1,01	1,49<br>4,65<br>2,19	0,23<br>2,11<br>0,31	0,54<br>4,41<br>0,41	0,14<br>2,97<br>0,59	0,51<br>3,97<br>1,36	0,19<br>2,09<br>3,97	0,43<br>2,55<br>5,60	-0,11<br>0,66<br>11,45	0,27<br>1,06<br>9,79		
100	180,60<br>-0,14<br>34,06	74,55<br>0,56<br>18,39	12,89<br>0,43<br>5,41	20,43<br>1,17<br>9,03	-0,01<br>1,29<br>1,17	0,92<br>2,09<br>1,61	0,01<br>3,23<br>0,35	0,37<br>3,43<br>0,40	-0,04<br>2,88<br>0,51	0,36<br>2,70<br>1,65	0,08<br>0,91<br>2,70	0,52<br>1,03<br>5,05	0,42<br>-0,15<br>19,07	0,46<br>0,38<br>11,90	-0,05<br>-0,40<br>44,56	0,27<br>0,16<br>14,12

R=0.2

τ_1 × frame rate (columns); τ_2 × frame rate (rows)

k = 0,001	0,2	0,5	1	2	5	10	20	50
0,5	1,11 0,50<br>1976,00 3657,80<br>5,38 1,46							
1	2,14 1,07<br>8,43 21,89<br>3,65 1,54	0,37 0,38<br>14,25 48,55<br>3,75 1,73						
2	2,39 1,85<br>0,84 2,52<br>1,76 0,90	0,42 0,67<br>0,69 2,25<br>1,83 0,93	-0,06 0,48<br>0,81 1,17<br>2,02 1,36					
5	0,69 0,92<br>0,01 0,01<br>0,43 0,33	0,06 0,32<br>0,01 0,02<br>0,24 0,26	0,00 0,19<br>0,00 0,02<br>0,10 0,10	-0,03 0,23<br>0,06 0,14<br>0,57 0,47				
10	0,21 0,49<br>0,00 0,01<br>0,39 0,28	0,02 0,15<br>0,00 0,01<br>0,06 0,09	-0,01 0,09<br>0,00 0,01<br>0,02 0,01	0,01 0,08<br>0,00 0,02<br>0,06 0,05	0,01 0,19<br>0,12 0,19<br>0,88 0,75			
20	0,16 0,39<br>0,00 0,01<br>0,26 0,21	0,02 0,11<br>0,00 0,01<br>0,04 0,04	0,00 0,05<br>0,00 0,01<br>0,01 0,01	0,00 0,04<br>0,00 0,01<br>0,02 0,01	0,01 0,07<br>0,01 0,03<br>0,11 0,08	0,00 0,20<br>0,16 0,19<br>0,93 0,72		
50	0,02 0,27<br>0,00 0,01<br>0,24 0,17	0,00 0,08<br>0,00 0,01<br>0,03 0,03	0,00 0,04<br>0,00 0,02<br>0,01 0,01	0,00 0,02<br>0,00 0,02<br>0,00 0,00	0,00 0,03<br>0,00 0,03<br>0,02 0,02	-0,01 0,06<br>0,01 0,08<br>0,09 0,07	-0,07 0,18<br>0,12 0,19<br>0,46 0,30	
100	-0,68 0,02<br>-0,29 0,01<br>1,30 0,09	-0,69 0,07<br>-0,34 0,02<br>1,26 0,22	0,00 0,03<br>0,02 0,08<br>0,02 0,02	0,00 0,02<br>0,02 0,08<br>0,02 0,02	0,00 0,03<br>0,11 0,20<br>0,03 0,03	-0,01 0,04<br>0,19 0,30<br>0,05 0,03	-0,09 0,09<br>-0,11 0,11<br>0,22 0,16	0,13 0,13<br>-0,38 0,07<br>13,12 5,30

k=0,002	0,2	0,5	1	2	5	10	20	50
0,5	1,05 1,02<br>8656,34 10462,24<br>5,31 1,66							
1	2,62 1,28<br>2651,47 4827,24<br>4,74 1,40	0,50 0,52<br>2378,47 3269,63<br>4,56 1,39						
2	3,83 2,12<br>690,76 1218,09<br>2,44 1,13	0,77 0,84<br>449,85 959,59<br>2,48 1,17	0,44 0,32<br>1207,88 1686,00<br>3,60 1,15					
5	2,18 2,47<br>4,32 24,77<br>0,70 0,45	0,24 0,67<br>0,03 0,07<br>0,69 0,40	0,03 0,39<br>0,03 0,08<br>0,51 0,45	0,06 0,36<br>7,73 42,23<br>1,29 0,98				
10	0,70 1,00<br>0,01 0,01<br>0,58 0,39	0,02 0,32<br>0,00 0,02<br>0,45 0,87	0,02 0,17<br>0,00 0,02<br>0,05 0,06	0,04 0,17<br>0,02 0,04<br>0,16 0,15	-0,08 0,32<br>15,78 36,99<br>1,43 0,92			
20	0,38 0,76<br>0,01 0,01<br>0,56 0,38	0,00 0,19<br>0,00 0,01<br>0,12 0,13	0,01 0,12<br>0,00 0,02<br>0,02 0,01	0,00 0,08<br>0,00 0,02<br>0,04 0,03	0,03 0,16<br>0,04 0,14<br>0,24 0,22	-0,03 0,30<br>4,02 8,74<br>1,12 0,85		
50	0,24 0,50<br>0,01 0,03<br>0,43 0,34	0,00 0,19<br>0,00 0,04<br>0,21 0,70	0,01 0,08<br>0,01 0,04<br>0,02 0,01	0,01 0,06<br>0,02 0,05<br>0,01 0,01	0,01 0,08<br>0,04 0,09<br>0,08 0,37	-0,01 0,13<br>0,26 0,51<br>0,17 0,14	-0,10 0,26<br>0,71 1,07<br>0,63 0,52	
100	-0,07 0,53<br>0,08 0,40<br>0,97 1,02	0,02 0,18<br>0,12 0,27<br>0,15 0,34	-0,02 0,10<br>0,08 0,23<br>0,38 1,45	0,00 0,05<br>0,27 0,82<br>0,05 0,07	0,00 0,05<br>1,05 1,86<br>0,10 0,12	-0,02 0,07<br>1,30 1,76<br>0,18 0,10	-0,14 0,17<br>0,22 0,39<br>0,34 0,25	-0,40 0,16<br>0,09 0,43<br>2,71 1,79

k=0,005	0,2	0,5	1	2	5	10	20	50
0,5	1,23 0,90<br>1169,55 1979,87<br>7,40 3,16							
1	2,66 1,38<br>337,48 557,73<br>6,02 2,98	0,58 0,54<br>343,86 610,32<br>6,79 3,12						
2	3,14 2,07<br>100,15 224,01<br>3,46 2,45	0,70 0,82<br>124,84 234,64<br>2,90 1,48	-0,10 0,43<br>99,45 210,21<br>3,58 2,85					
5	4,59 4,25<br>11,43 31,15<br>1,64 0,81	0,97 1,26<br>1,51 7,19<br>1,69 0,68	0,36 0,79<br>5,99 21,54<br>1,69 0,82	0,03 0,54<br>11,22 27,06<br>2,11 1,23				
10	3,17 4,69<br>0,42 2,10<br>0,99 0,87	0,36 0,86<br>0,02 0,05<br>0,76 0,55	0,10 0,54<br>0,08 0,48<br>0,48 0,41	0,13 0,38<br>0,57 2,85<br>0,50 0,46	-0,09 0,32<br>3,91 8,09<br>1,82 1,43			
20	2,34 4,49<br>0,17 0,91<br>0,56 0,36	0,02 0,45<br>0,01 0,03<br>0,54 0,48	0,02 0,27<br>0,01 0,04<br>0,10 0,15	0,05 0,24<br>0,03 0,07<br>0,11 0,10	0,03 0,29<br>0,80 1,88<br>0,48 0,35	-0,07 0,31<br>1,20 1,93<br>1,70 1,36		
50	1,10 1,70<br>0,11 0,49<br>0,49 0,36	0,16 0,39<br>0,08 0,12<br>0,32 0,29	0,01 0,19<br>0,04 0,13<br>0,05 0,05	-0,01 0,13<br>0,06 0,17<br>0,02 0,02	0,03 0,30<br>1,37 3,00<br>0,11 0,09	-0,07 0,22<br>1,28 1,60<br>0,27 0,18	-0,07 0,31<br>2,56 3,43<br>1,27 1,47	
100	0,15 1,35<br>-0,14 0,30<br>0,86 0,43	-0,33 0,38<br>0,12 0,79<br>1,17 0,61	0,02 0,17<br>1,19 1,83<br>0,15 0,13	0,00 0,10<br>2,77 3,37<br>0,19 0,17	-0,04 0,10<br>2,95 3,21<br>0,23 0,18	-0,11 0,14<br>1,01 1,22<br>0,29 0,15	-0,10 0,33<br>0,95 1,68<br>1,37 1,84	-0,43 0,17<br>0,63 0,93<br>3,66 2,20

k=0,01	0,2	0,5	1	2	5	10	20	50
0,5	1,65 1,22<br>1769,15 2722,34<br>9,85 4,95							
1	2,75 1,70<br>372,76 692,86<br>7,53 4,74	0,61 0,59<br>424,19 716,87<br>8,42 5,19						
2	2,68 1,90<br>159,93 274,72<br>8,22 7,34	0,52 0,78<br>193,11 351,98<br>7,25 7,10	-0,21 0,35<br>162,16 271,06<br>7,85 6,47					
5	7,66 4,90<br>27,45 51,03<br>3,96 3,89	1,78 1,63<br>15,40 39,94<br>2,82 1,67	0,54 0,78<br>27,17 47,30<br>2,60 1,91	0,01 0,44<br>29,62 54,27<br>3,43 3,12				
10	11,84 7,13<br>7,59 15,67<br>2,25 2,28	1,51 1,92<br>1,93 7,04<br>1,41 1,44	0,57 1,03<br>2,59 8,22<br>1,17 0,64	0,19 0,56<br>6,93 14,22<br>1,31 1,24	-0,11 0,37<br>8,64 13,60<br>3,27 3,65			
20	12,62 12,00<br>2,26 5,05<br>1,36 1,83	1,16 1,59<br>0,41 1,78<br>0,76 0,54	0,25 0,70<br>0,42 1,97<br>0,50 0,43	0,17 0,48<br>0,81 3,15<br>0,32 0,30	0,20 0,42<br>5,20 7,20<br>1,05 1,29	-0,10 0,30<br>2,54 3,73<br>3,52 3,80		
50	9,59 16,93<br>1,61 2,98<br>1,14 2,19	0,46 0,85<br>1,33 3,86<br>0,68 0,55	0,12 0,39<br>2,02 4,38<br>0,24 0,30	0,05 0,24<br>3,76 6,30<br>0,12 0,13	-0,05 0,25<br>5,27 7,55<br>0,25 0,14	-0,12 0,31<br>3,37 4,04<br>0,57 0,95	-0,28 0,26<br>1,13 1,52<br>4,29 4,71	
100	46,54 51,99<br>0,88 1,95<br>4,42 5,96	-0,11 0,61<br>1,27 2,38<br>1,30 0,76	-0,07 0,30<br>3,96 4,14<br>0,42 0,34	-0,02 0,19<br>7,15 6,81<br>0,31 0,23	-0,05 0,18<br>7,90 7,09<br>0,39 0,21	-0,16 0,23<br>2,90 2,86<br>0,47 0,58	-0,19 0,26<br>0,29 0,74<br>5,91 5,82	-0,14 0,22<br>-0,43 0,15<br>22,04 9,01

R=0.3

τ_1 × frame rate

τ_2 × frame rate

k = 0,001	0,2	0,5	1	2	5	10	20	50
0,5	0,95 0,47<br>1525,47 3554,16<br>3,52 0,97							
1	1,49 1,07<br>33,46 131,79<br>2,05 0,96	0,17 0,42<br>13,74 32,03<br>2,25 1,17						
2	1,72 1,55<br>0,35 0,93<br>0,94 0,51	0,25 0,61<br>0,31 0,67<br>1,14 0,50	-0,12 0,36<br>3,17 8,62<br>1,34 0,75					
5	0,29 0,65<br>0,01 0,01<br>0,35 0,26	0,02 0,27<br>0,00 0,02<br>0,14 0,17	0,02 0,13<br>0,01 0,02<br>0,08 0,07	0,01 0,15<br>0,05 0,09<br>0,37 0,27				
10	0,08 0,38<br>0,00 0,01<br>0,26 0,22	0,00 0,11<br>0,00 0,01<br>0,04 0,04	0,01 0,06<br>0,00 0,01<br>0,02 0,01	0,01 0,05<br>0,01 0,02<br>0,05 0,04	0,00 0,15<br>0,12 0,17<br>0,57 0,48			
20	0,07 0,30<br>0,00 0,01<br>0,21 0,18	0,00 0,07<br>0,00 0,01<br>0,03 0,02	0,00 0,04<br>0,00 0,01<br>0,01 0,00	0,00 0,03<br>0,00 0,01<br>0,01 0,01	-0,01 0,06<br>0,00 0,04<br>0,09 0,06	-0,02 0,15<br>0,14 0,20<br>0,53 0,43		
50	-0,06 0,21<br>0,00 0,02<br>0,21 0,17	0,01 0,05<br>0,00 0,02<br>0,02 0,02	0,00 0,02<br>0,00 0,02<br>0,01 0,01	0,00 0,02<br>0,00 0,03<br>0,00 0,00	0,00 0,02<br>0,01 0,05<br>0,01 0,01	0,00 0,05<br>0,04 0,10<br>0,07 0,05	-0,08 0,13<br>0,11 0,18<br>0,34 0,26	
100	-0,81 0,02<br>-0,25 0,03<br>1,77 0,08	-0,27 0,11<br>-0,26 0,08<br>0,34 0,14	0,00 0,02<br>0,02 0,08<br>0,02 0,02	0,00 0,02<br>0,03 0,10<br>0,03 0,02	0,00 0,02<br>0,24 0,44<br>0,06 0,06	0,00 0,03<br>0,25 0,32<br>0,07 0,05	-0,07 0,07<br>-0,12 0,15<br>0,20 0,15	0,07 0,10<br>-0,40 0,06<br>7,70 3,18

τ_2 × frame rate

k=0,002	0,2	0,5	1	2	5	10	20	50
0,5	1,15 0,57<br>10715,46 14528,02<br>3,89 1,06							
1	2,19 1,43<br>2494,51 4120,77<br>2,88 1,10	0,45 0,41<br>2209,88 3874,57<br>3,05 0,80						
2	2,77 1,99<br>324,79 656,10<br>1,48 0,74	0,62 0,73<br>249,79 669,17<br>1,57 0,69	0,10 0,34<br>1004,26 1617,42<br>1,68 0,77					
5	1,00 1,39<br>0,03 0,05<br>0,49 0,30	-0,02 0,42<br>0,01 0,04<br>0,41 0,29	0,01 0,27<br>0,02 0,06<br>0,74 0,43	0,03 0,28<br>7,01 31,49<br>0,74 0,57				
10	0,38 0,76<br>0,01 0,02<br>0,43 0,34	0,00 0,23<br>0,00 0,02<br>0,14 0,16	0,04 0,12<br>0,01 0,02<br>0,04 0,03	0,00 0,11<br>0,01 0,04<br>0,09 0,08	-0,03 0,25<br>2,79 8,07<br>0,85 0,56			
20	0,10 0,55<br>0,00 0,01<br>0,43 0,29	-0,02 0,17<br>0,00 0,02<br>0,08 0,08	0,00 0,07<br>0,00 0,02<br>0,01 0,01	0,01 0,06<br>0,01 0,03<br>0,03 0,02	0,01 0,08<br>0,03 0,06<br>0,13 0,11	-0,08 0,24<br>2,13 5,40<br>0,73 0,59		
50	0,15 0,44<br>0,01 0,04<br>0,32 0,26	0,01 0,11<br>0,01 0,04<br>0,05 0,04	0,00 0,05<br>0,01 0,04<br>0,01 0,01	0,00 0,04<br>0,02 0,06<br>0,01 0,00	0,00 0,04<br>0,03 0,12<br>0,03 0,02	0,00 0,08<br>0,67 1,38<br>0,12 0,09	-0,14 0,20<br>0,83 1,55<br>0,42 0,35	
100	0,12 0,39<br>0,15 0,28<br>0,34 0,39	0,00 0,12<br>0,10 0,30<br>0,09 0,07	0,01 0,05<br>0,24 0,59<br>0,07 0,07	0,00 0,03<br>0,40 1,09<br>0,08 0,08	-0,01 0,05<br>1,36 2,24<br>0,24 1,02	-0,02 0,05<br>1,87 1,85<br>0,22 0,14	-0,12 0,14<br>0,47 1,06<br>0,38 0,26	-0,37 0,15<br>0,00 0,40<br>2,44 1,33

τ_2 × frame rate

k=0,005	0,2	0,5	1	2	5	10	20	50
0,5	1,41 1,00<br>1449,88 2691,63<br>6,01 2,91							
1	2,40 1,33<br>269,39 555,39<br>4,95 2,39	0,45 0,49<br>524,38 713,84<br>4,67 2,47						
2	2,70 1,89<br>89,15 196,61<br>2,46 1,86	0,60 0,83<br>161,42 291,05<br>2,15 1,29	-0,19 0,38<br>114,76 227,41<br>2,24 1,54					
5	3,37 3,26<br>4,64 20,85<br>1,00 0,54	0,51 1,02<br>1,05 8,61<br>0,94 0,43	0,25 0,60<br>2,88 11,47<br>1,02 0,49	-0,11 0,40<br>16,56 33,46<br>1,02 0,71				
10	1,99 2,53<br>0,09 0,37<br>0,58 0,42	0,21 0,56<br>0,03 0,06<br>0,44 0,36	0,03 0,31<br>0,03 0,11<br>0,18 0,20	0,01 0,26<br>0,21 1,37<br>0,27 0,22	-0,11 0,33<br>5,77 10,17<br>1,14 0,96			
20	0,78 1,22<br>0,01 0,03<br>0,50 0,37	0,02 0,37<br>0,01 0,05<br>0,32 0,29	-0,03 0,18<br>0,00 0,05<br>0,06 0,07	0,02 0,15<br>0,03 0,08<br>0,07 0,06	0,03 0,22<br>0,51 1,46<br>0,31 0,26	-0,15 0,31<br>1,28 1,72<br>0,99 0,86		
50	0,60 0,97<br>0,11 0,49<br>0,45 0,32	0,05 0,30<br>0,07 0,16<br>0,22 0,21	0,00 0,14<br>0,05 0,15<br>0,04 0,04	0,03 0,09<br>0,32 0,97<br>0,04 0,06	-0,01 0,11<br>1,60 2,76<br>0,15 0,11	-0,07 0,19<br>2,06 2,64<br>0,32 0,20	-0,18 0,28<br>3,59 4,81<br>0,72 0,71	
100	-0,34 0,67<br>-0,15 0,46<br>1,30 0,48	-0,28 0,29<br>0,07 0,57<br>0,61 0,44	0,00 0,10<br>1,42 1,78<br>0,19 0,17	-0,01 0,08<br>2,29 2,73<br>0,21 0,18	-0,03 0,06<br>4,01 3,84<br>0,31 0,23	-0,08 0,11<br>5,63 4,58<br>0,51 0,38	-0,18 0,28<br>1,44 2,38<br>1,05 1,27	-0,44 0,15<br>0,61 0,83<br>3,14 1,71

τ_2 × frame rate

k=0,01	0,2	0,5	1	2	5	10	20	50
0,5	1,67 1,46<br>1247,49 2315,46<br>7,47 4,32							
1	2,60 1,72<br>449,51 702,63<br>6,37 3,72	0,60 0,66<br>309,42 596,41<br>6,60 4,13						
2	2,40 1,71<br>110,86 236,01<br>5,65 4,09	0,43 0,76<br>234,18 407,67<br>4,78 4,83	-0,29 0,38<br>149,60 302,43<br>6,13 5,97					
5	5,79 3,88<br>19,23 44,00<br>1,96 1,51	1,06 1,37<br>19,87 45,63<br>1,43 0,63	0,18 0,71<br>19,63 46,86<br>1,38 0,66	-0,14 0,40<br>35,15 53,62<br>1,93 1,86				
10	6,12 5,45<br>4,44 11,35<br>1,12 1,42	0,81 1,23<br>0,20 0,39<br>0,83 0,47	0,28 0,68<br>3,11 9,98<br>0,69 0,39	0,11 0,47<br>5,27 12,77<br>0,59 0,40	-0,14 0,29<br>12,09 17,39<br>2,51 2,43			
20	4,81 6,35<br>1,21 3,59<br>0,70 1,11	0,37 0,74<br>0,07 0,15<br>0,56 0,43	0,04 0,32<br>0,06 0,11<br>0,22 0,26	0,08 0,28<br>0,65 3,01<br>0,15 0,11	0,03 0,29<br>4,11 5,52<br>0,50 0,79	-0,22 0,30<br>2,40 4,00<br>2,30 2,80		
50	2,37 5,22<br>1,40 2,96<br>0,60 0,90	0,16 0,60<br>1,15 2,80<br>0,52 0,42	0,04 0,25<br>1,86 3,46<br>0,17 0,16	0,02 0,19<br>4,39 6,64<br>0,17 0,18	-0,05 0,20<br>6,86 6,88<br>0,34 0,18	-0,18 0,25<br>4,90 5,93<br>0,56 0,45	-0,25 0,21<br>1,25 1,66<br>2,84 2,62	
100	1,71 10,84<br>0,34 1,37<br>1,16 0,64	-0,16 0,40<br>1,10 1,73<br>0,81 0,55	-0,03 0,21<br>3,15 2,99<br>0,28 0,19	-0,04 0,14<br>5,50 4,54<br>0,33 0,23	-0,08 0,12<br>7,46 5,44<br>0,44 0,25	-0,20 0,18<br>4,65 4,11<br>0,58 0,21	-0,24 0,23<br>0,23 0,87<br>3,70 3,92	-0,21 0,20<br>-0,43 0,19<br>15,77 5,83

R=0.4

τ_1 × frame rate

τ_2 × frame rate

k = 0,001	0,2		0,5		1		2		5		10		20		50	
0,5	0,72	0,44														
	1705,29	3809,26														
	2,54	0,73														
1	1,26	0,99	0,11	0,44												
	79,93	389,09	35,61	92,11												
	1,53	0,71	1,72	1,00												
2	1,29	1,39	0,09	0,50	-0,22	0,37										
	0,22	0,30	0,24	0,30	1,30	3,14										
	0,73	0,36	0,83	0,38	0,79	0,59										
5	0,16	0,52	-0,03	0,19	-0,02	0,10	-0,01	0,13								
	0,01	0,01	0,00	0,02	0,00	0,03	0,03	0,08								
	0,32	0,24	0,07	0,09	0,06	0,04	0,30	0,22								
10	0,02	0,32	0,01	0,09	0,01	0,04	0,00	0,04	0,00	0,12						
	0,00	0,01	0,00	0,01	0,00	0,01	0,00	0,02	0,12	0,21						
	0,25	0,18	0,02	0,02	0,01	0,01	0,04	0,03	0,43	0,31						
20	0,01	0,25	0,01	0,07	0,00	0,03	0,00	0,02	-0,01	0,04	-0,05	0,11				
	0,00	0,01	0,00	0,01	0,00	0,01	0,00	0,02	0,00	0,04	0,09	0,18				
	0,18	0,14	0,02	0,02	0,00	0,00	0,01	0,01	0,06	0,05	0,39	0,29				
50	-0,29	0,16	0,00	0,04	0,00	0,02	0,00	0,01	0,00	0,02	0,00	0,04	-0,07	0,11		
	-0,03	0,02	0,01	0,02	0,00	0,03	0,01	0,03	0,01	0,05	0,06	0,14	0,11	0,19		
	0,36	0,16	0,02	0,01	0,01	0,00	0,00	0,00	0,01	0,01	0,06	0,05	0,33	0,26		
100	-0,77	0,04	-0,01	0,05	0,00	0,02	0,00	0,01	0,00	0,02	-0,01	0,02	-0,06	0,06	-0,03	0,07
	-0,24	0,03	0,01	0,11	0,04	0,13	0,09	0,25	0,39	0,59	0,31	0,44	-0,16	0,12	-0,40	0,05
	1,37	0,12	0,05	0,04	0,03	0,03	0,04	0,04	0,08	0,08	0,08	0,07	0,21	0,17	4,73	2,26

τ_2 × frame rate

k=0,002	0,2		0,5		1		2		5		10		20		50	
0,5	0,98	0,92														
	6538,69	7870,71														
	2,93	0,81														
1	1,84	1,42	0,39	0,32												
	3512,71	5191,19	2067,51	3474,25												
	1,87	0,94	2,26	0,69												
2	2,37	1,81	0,13	0,58	0,01	0,35										
	496,85	2257,94	68,82	230,77	649,85	1236,48										
	1,01	0,55	0,89	0,46	1,15	0,69										
5	0,67	0,90	0,03	0,37	0,00	0,22	-0,06	0,23								
	0,02	0,04	0,02	0,05	0,03	0,07	4,11	26,65								
	0,32	0,27	0,27	0,20	0,18	0,15	0,59	0,42								
10	0,15	0,56	0,01	0,20	0,02	0,09	0,02	0,09	-0,04	0,20						
	0,00	0,02	0,00	0,02	0,01	0,03	0,02	0,05	4,90	14,12						
	0,42	0,25	0,07	0,08	0,03	0,03	0,10	0,07	0,59	0,44						
20	0,17	0,44	0,00	0,11	0,01	0,06	0,00	0,04	0,01	0,08	-0,08	0,20				
	0,00	0,02	0,00	0,02	0,00	0,02	0,00	0,03	0,04	0,10	2,04	3,33				
	0,30	0,22	0,05	0,04	0,01	0,01	0,03	0,02	0,14	0,10	0,60	0,40				
50	0,11	0,35	0,00	0,10	0,00	0,04	0,00	0,03	0,00	0,03	0,00	0,07	-0,17	0,19		
	0,02	0,05	0,01	0,05	0,01	0,06	0,01	0,06	0,07	0,15	0,60	1,16	1,08	2,13		
	0,23	0,20	0,05	0,04	0,02	0,01	0,01	0,01	0,02	0,02	0,12	0,09	0,53	0,37		
100	0,50	4,51	-0,01	0,09	-0,01	0,08	0,01	0,03	-0,01	0,07	-0,01	0,04	-0,12	0,12	-0,39	0,15
	0,24	0,70	0,27	0,81	0,43	1,00	0,85	1,47	1,76	2,28	2,27	1,94	0,60	1,10	-0,14	0,23
	0,56	0,96	0,10	0,09	0,40	1,71	0,13	0,13	0,38	0,92	0,29	0,17	0,45	0,29	2,48	1,00

τ_2 × frame rate

k=0,005	0,2		0,5		1		2		5		10		20		50	
0,5	1,20	0,77														
	1262,45	1940,50														
	4,32	2,17														
1	2,36	1,11	0,35	0,53												
	404,17	750,50	398,27	671,65												
	3,79	2,15	3,94	2,30												
2	2,38	1,68	0,36	0,71	-0,23	0,40										
	112,30	229,17	101,30	214,76	166,11	329,31										
	1,55	0,95	1,57	1,11	2,21	1,73										
5	2,10	2,42	0,27	0,78	0,07	0,48	-0,12	0,34								
	4,45	19,21	0,15	0,45	1,59	7,28	14,54	35,11								
	0,66	0,36	0,69	0,35	0,64	0,35	0,74	0,49								
10	1,26	1,60	0,09	0,46	-0,03	0,26	0,04	0,20	-0,15	0,31						
	0,04	0,05	0,02	0,06	0,01	0,07	0,12	0,29	6,52	11,56						
	0,45	0,32	0,31	0,31	0,12	0,13	0,22	0,18	0,90	0,82						
20	0,66	0,83	0,05	0,28	0,00	0,14	0,01	0,13	0,00	0,19	-0,16	0,26				
	0,03	0,04	0,01	0,06	0,01	0,06	0,03	0,09	0,88	2,44	1,69	2,30				
	0,31	0,27	0,16	0,18	0,03	0,02	0,07	0,05	0,29	0,20	0,93	0,91				
50	0,33	0,85	-0,03	0,22	0,00	0,11	-0,01	0,07	-0,01	0,08	-0,07	0,13	-0,21	0,23		
	0,07	0,19	0,07	0,19	0,18	0,52	0,33	1,01	2,40	3,26	1,85	2,37	4,19	7,12		
	0,50	0,33	0,16	0,14	0,05	0,05	0,05	0,06	0,19	0,15	0,35	0,21	0,89	0,79		
100	-0,43	0,50	-0,07	0,24	-0,01	0,09	0,00	0,05	-0,02	0,05	-0,07	0,10	-0,22	0,22	-0,47	0,18
	-0,15	0,39	0,56	0,97	1,83	2,06	2,60	2,46	3,00	2,60	3,76	2,93	2,93	3,14	0,37	0,75
	1,10	0,43	0,28	0,21	0,21	0,18	0,25	0,18	0,30	0,21	0,47	0,20	1,13	1,03	2,65	1,33

τ_2 × frame rate

k=0,01	0,2		0,5		1		2		5		10		20		50	
0,5	1,34	1,29														
	1830,48	3179,45														
	5,71	3,69														
1	2,74	1,58	0,42	0,63												
	632,45	913,89	439,10	751,74												
	5,17	3,56	4,65	3,29												
2	2,17	1,74	0,32	0,60	-0,25	0,33										
	190,02	350,32	149,03	295,82	188,13	308,45										
	5,39	5,01	4,83	5,18	6,09	5,68										
5	4,33	3,63	0,68	1,03	0,13	0,57	-0,09	0,39								
	19,52	45,61	13,82	44,66	24,14	54,61	44,12	75,59								
	1,35	1,44	1,03	0,33	0,88	0,40	1,59	1,59								
10	3,44	3,71	0,29	0,87	0,11	0,51	0,04	0,34	-0,25	0,28						
	2,95	9,58	0,87	4,65	1,43	5,52	3,99	11,58	13,95	20,59						
	0,64	0,73	0,68	0,44	0,41	0,27	0,36	0,27	1,88	2,23						
20	2,93	4,90	0,12	0,55	0,08	0,32	0,01	0,22	-0,06	0,29	-0,21	0,27				
	0,88	4,06	0,05	0,13	0,57	2,86	1,06	4,50	5,45	7,28	5,72	7,05				
	0,44	0,31	0,40	0,31	0,13	0,15	0,12	0,11	0,19	0,25	2,42	2,39				
50	1,30	2,00	0,11	0,42	0,04	0,20	-0,01	0,13	-0,04	0,13	-0,20	0,21	-0,30	0,24		
	1,27	2,44	1,18	2,24	1,78	3,21	3,03	5,14	5,68	6,70	3,87	4,41	0,94	1,67		
	0,54	0,41	0,39	0,30	0,16	0,17	0,16	0,19	0,34	0,22	0,64	0,51	3,17	3,12		
100	0,21	1,26	-0,16	0,36	-0,04	0,15	-0,03	0,11	-0,08	0,11	-0,17	0,18	-0,35	0,23	-0,33	0,21
	0,75	1,63	1,38	1,80	3,99	3,40	4,86	3,41	5,97	4,41	3,99	3,42	0,63	1,55	-0,27	0,35
	1,02	0,58	0,55	0,40	0,34	0,24	0,36	0,24	0,46	0,25	0,62	0,29	2,61	2,45	10,82	5,43

R=0.5

τ_1 × frame rate

τ_2 × frame rate (rows)

k = 0,001	0,2	0,5	1	2	5	10	20	50
0,5	0,73 0,49<br>1521,29 3292,46<br>2,26 0,88							
1	0,94 0,87<br>25,53 85,04<br>1,09 0,58	0,02 0,40<br>30,72 111,16<br>1,26 0,91						
2	0,92 1,00<br>0,20 0,37<br>0,51 0,28	0,01 0,43<br>0,26 0,43<br>0,61 0,30	-0,25 0,36<br>1,24 2,10<br>0,61 0,50					
5	0,08 0,49<br>0,01 0,02<br>0,28 0,20	-0,02 0,16<br>0,00 0,03<br>0,05 0,04	0,01 0,09<br>0,01 0,04<br>0,07 0,06	-0,02 0,11<br>0,04 0,10<br>0,28 0,20				
10	0,04 0,28<br>0,00 0,01<br>0,19 0,15	0,00 0,08<br>0,00 0,01<br>0,02 0,02	0,00 0,04<br>0,00 0,02<br>0,01 0,01	0,00 0,04<br>0,00 0,03<br>0,05 0,04	-0,02 0,11<br>0,13 0,27<br>0,42 0,34			
20	0,01 0,18<br>0,00 0,01<br>0,12 0,09	0,01 0,05<br>0,00 0,01<br>0,02 0,01	0,00 0,02<br>0,00 0,01<br>0,00 0,00	0,00 0,02<br>0,00 0,02<br>0,01 0,01	0,00 0,03<br>0,01 0,04<br>0,06 0,04	-0,04 0,10<br>0,13 0,21<br>0,41 0,31		
50	-0,35 0,13<br>-0,06 0,02<br>0,40 0,16	0,00 0,04<br>0,00 0,03<br>0,02 0,02	0,00 0,02<br>0,00 0,04<br>0,01 0,01	0,00 0,01<br>0,02 0,05<br>0,01 0,00	0,00 0,01<br>0,02 0,07<br>0,01 0,01	0,00 0,03<br>0,07 0,19<br>0,06 0,04	-0,10 0,11<br>0,01 0,17<br>0,46 0,34	
100	-0,72 0,04<br>-0,30 0,03<br>1,10 0,08	0,00 0,04<br>0,05 0,15<br>0,05 0,04	0,00 0,02<br>0,08 0,22<br>0,05 0,05	0,00 0,01<br>0,17 0,38<br>0,06 0,06	0,00 0,01<br>0,44 0,77<br>0,10 0,10	-0,01 0,02<br>0,36 0,58<br>0,10 0,09	-0,06 0,05<br>-0,26 0,10<br>0,23 0,18	-0,15 0,07<br>-0,38 0,07<br>3,58 1,80

τ_2 × frame rate (rows)

k=0,002	0,2	0,5	1	2	5	10	20	50
0,5	0,90 0,54<br>3587,82 6113,70<br>2,44 0,68							
1	1,81 1,10<br>5535,72 7788,99<br>1,57 0,54	0,31 0,47<br>1049,54 1760,62<br>1,99 0,79						
2	1,39 1,42<br>60,30 227,15<br>0,69 0,37	0,19 0,54<br>29,04 204,60<br>0,79 0,42	-0,02 0,33<br>975,97 1403,27<br>0,85 0,56					
5	0,42 0,89<br>0,02 0,05<br>0,35 0,24	0,03 0,31<br>0,02 0,05<br>0,19 0,15	-0,01 0,16<br>0,02 0,07<br>0,12 0,09	-0,06 0,18<br>0,14 0,25<br>0,41 0,31				
10	0,25 0,59<br>0,01 0,02<br>0,33 0,22	0,00 0,16<br>0,00 0,03<br>0,06 0,06	-0,01 0,08<br>0,00 0,04<br>0,03 0,02	0,00 0,07<br>0,02 0,05<br>0,08 0,07	-0,04 0,18<br>3,93 10,35<br>0,55 0,38			
20	0,10 0,42<br>0,01 0,02<br>0,28 0,18	0,00 0,11<br>0,00 0,03<br>0,04 0,03	0,00 0,05<br>0,00 0,03<br>0,01 0,01	0,00 0,04<br>0,01 0,04<br>0,02 0,02	-0,01 0,07<br>0,04 0,11<br>0,14 0,10	-0,08 0,16<br>1,77 2,43<br>0,49 0,34		
50	0,04 0,32<br>0,02 0,06<br>0,25 0,17	-0,06 0,16<br>-0,01 0,07<br>0,97 2,70	0,00 0,03<br>0,02 0,08<br>0,02 0,01	0,00 0,03<br>0,03 0,09<br>0,01 0,01	0,00 0,03<br>0,16 0,63<br>0,03 0,04	-0,02 0,05<br>1,02 1,76<br>0,18 0,12	-0,18 0,19<br>1,47 2,49<br>0,68 0,43	
100	-0,03 0,36<br>0,55 1,02<br>0,90 1,75	-0,05 0,22<br>0,89 1,36<br>1,42 3,81	0,00 0,03<br>0,62 1,27<br>0,12 0,14	0,00 0,03<br>1,19 1,91<br>0,17 0,17	0,00 0,02<br>2,13 2,28<br>0,25 0,22	-0,02 0,03<br>1,92 1,96<br>0,30 0,20	-0,13 0,13<br>0,92 1,72<br>0,57 0,35	-0,42 0,14<br>-0,13 0,30<br>2,51 0,84

τ_2 × frame rate (rows)

k=0,005	0,2	0,5	1	2	5	10	20	50
0,5	0,84 0,97<br>1360,28 2167,32<br>3,46 2,19							
1	1,84 1,19<br>512,28 867,88<br>2,97 2,12	0,28 0,51<br>478,43 791,67<br>3,12 1,82						
2	2,03 1,85<br>139,38 267,27<br>1,53 1,44	0,07 0,61<br>134,06 275,58<br>1,20 0,61	-0,32 0,37<br>140,11 292,50<br>2,27 1,82					
5	1,49 1,76<br>1,83 12,63<br>0,49 0,28	0,22 0,64<br>0,15 0,26<br>0,54 0,28	0,02 0,41<br>3,71 17,88<br>0,44 0,30	-0,12 0,30<br>21,00 41,49<br>0,53 0,36				
10	0,77 1,33<br>0,04 0,08<br>0,41 0,26	0,02 0,38<br>0,02 0,09<br>0,26 0,24	-0,01 0,18<br>0,02 0,09<br>0,08 0,07	0,00 0,16<br>0,12 0,25<br>0,19 0,16	-0,17 0,28<br>7,62 12,00<br>0,70 0,51			
20	0,48 0,85<br>0,04 0,05<br>0,39 0,32	0,02 0,27<br>0,01 0,07<br>0,14 0,15	0,01 0,11<br>0,02 0,08<br>0,02 0,02	0,00 0,10<br>0,04 0,15<br>0,06 0,05	-0,02 0,13<br>0,79 2,34<br>0,24 0,17	-0,24 0,25<br>1,05 1,61<br>0,85 0,80		
50	0,14 0,74<br>0,13 0,47<br>0,49 0,34	0,01 0,19<br>0,36 1,02<br>0,13 0,12	0,01 0,09<br>0,36 1,26<br>0,07 0,09	0,00 0,06<br>0,79 1,80<br>0,09 0,13	0,00 0,06<br>2,99 3,73<br>0,24 0,20	-0,11 0,13<br>2,11 2,35<br>0,49 0,28	-0,22 0,20<br>4,72 6,41<br>1,37 0,96	
100	-0,36 0,54<br>-0,09 0,45<br>0,90 0,45	-0,05 0,17<br>1,39 1,51<br>0,25 0,21	-0,02 0,07<br>2,28 2,30<br>0,28 0,21	-0,02 0,05<br>2,89 2,86<br>0,32 0,24	-0,03 0,04<br>3,38 2,60<br>0,37 0,23	-0,09 0,09<br>4,02 2,83<br>0,58 0,33	-0,25 0,20<br>3,00 3,37<br>1,16 0,59	-0,48 0,15<br>0,33 0,64<br>2,98 1,42

τ_2 × frame rate (rows)

k=0,01	0,2	0,5	1	2	5	10	20	50
0,5	1,64 1,32<br>1033,72 1885,19<br>5,83 3,79							
1	2,31 1,62<br>580,99 775,96<br>4,69 3,36	0,46 0,67<br>602,03 923,59<br>4,77 3,41						
2	1,87 1,54<br>159,31 254,97<br>5,03 4,30	0,04 0,54<br>129,71 288,47<br>4,45 4,82	-0,34 0,36<br>198,74 374,24<br>6,40 5,96					
5	3,32 3,47<br>22,19 52,35<br>0,87 0,77	0,21 0,82<br>4,71 21,47<br>0,80 0,33	-0,02 0,48<br>18,01 45,11<br>0,73 0,45	-0,16 0,35<br>41,96 65,72<br>1,46 1,94				
10	2,81 3,38<br>2,13 8,89<br>0,53 0,35	0,10 0,74<br>0,14 0,43<br>0,58 0,36	-0,02 0,38<br>0,71 5,95<br>0,30 0,24	-0,03 0,30<br>6,36 15,80<br>0,31 0,22	-0,25 0,29<br>14,35 19,72<br>1,73 2,17			
20	1,41 2,06<br>0,38 1,85<br>0,44 0,30	0,18 0,54<br>0,11 0,27<br>0,34 0,30	0,04 0,28<br>0,87 3,59<br>0,09 0,09	-0,01 0,19<br>1,33 4,05<br>0,11 0,09	-0,09 0,23<br>6,84 9,75<br>0,40 0,27	-0,29 0,24<br>4,66 6,82<br>2,18 2,27		
50	0,66 1,25<br>1,08 1,94<br>0,57 0,39	0,01 0,38<br>2,46 3,47<br>0,36 0,27	-0,03 0,17<br>2,92 4,26<br>0,21 0,21	-0,01 0,11<br>4,93 5,58<br>0,29 0,26	-0,06 0,13<br>8,02 7,06<br>0,51 0,27	-0,16 0,21<br>5,05 4,94<br>0,68 0,34	-0,33 0,19<br>1,23 2,44<br>3,39 2,94	
100	0,01 0,83<br>0,50 1,07<br>0,82 0,47	-0,06 0,32<br>2,48 2,52<br>0,41 0,32	-0,06 0,15<br>4,30 3,22<br>0,40 0,24	-0,05 0,10<br>4,31 3,76<br>0,36 0,27	-0,07 0,10<br>5,26 3,96<br>0,52 0,28	-0,18 0,17<br>3,43 2,69<br>0,70 0,29	-0,36 0,22<br>0,55 1,52<br>2,95 2,75	-0,36 0,19<br>-0,30 0,30<br>10,56 5,18

R=0.6

τ_1 × frame rate (columns); *τ_2 × frame rate* (rows)

k = 0,001	0,2		0,5		1		2		5		10		20		50	
0,5	0,58	0,54														
	1685,22	3945,57														
	1,97	0,86														
1	0,75	0,71	-0,09	0,34												
	74,68	319,16	146,74	818,44												
	0,91	0,41	0,93	0,69												
2	0,59	0,80	-0,05	0,39	-0,18	0,29										
	0,15	0,19	0,21	0,30	2,36	5,72										
	0,43	0,21	0,46	0,27	0,61	0,39										
5	0,03	0,42	-0,02	0,13	0,01	0,07	-0,02	0,09								
	0,00	0,03	0,00	0,03	0,02	0,05	0,05	0,11								
	0,24	0,19	0,03	0,03	0,07	0,06	0,27	0,20								
10	0,02	0,29	0,00	0,06	0,01	0,04	0,00	0,03	-0,05	0,09						
	0,00	0,02	0,00	0,02	0,01	0,02	0,00	0,03	0,09	0,22						
	0,18	0,14	0,01	0,01	0,02	0,01	0,05	0,03	0,44	0,33						
20	0,01	0,16	0,00	0,04	0,00	0,02	0,00	0,02	0,00	0,03	-0,04	0,09				
	0,00	0,01	0,00	0,02	0,00	0,02	0,01	0,02	0,01	0,06	0,15	0,22				
	0,11	0,09	0,01	0,01	0,00	0,00	0,01	0,01	0,07	0,05	0,44	0,33				
50	-0,35	0,10	0,00	0,03	0,00	0,02	0,00	0,01	0,00	0,01	0,00	0,02	-0,12	0,12		
	-0,09	0,02	0,01	0,04	0,00	0,04	0,01	0,06	0,02	0,10	0,16	0,27	-0,06	0,16		
	0,37	0,11	0,02	0,01	0,01	0,01	0,01	0,01	0,01	0,01	0,06	0,05	0,61	0,48		
100	-0,64	0,06	0,00	0,03	0,00	0,02	0,00	0,01	0,00	0,01	-0,01	0,01	-0,07	0,06	-0,14	0,05
	-0,37	0,03	0,15	0,28	0,22	0,48	0,30	0,57	0,74	0,94	0,10	0,31	-0,36	0,10	-0,44	0,05
	0,88	0,10	0,07	0,06	0,08	0,08	0,10	0,09	0,16	0,14	0,07	0,06	0,33	0,28	4,51	1,85

k=0,002	0,2		0,5		1		2		5		10		20		50	
0,5	0,85	0,71														
	13891,70	37241,24														
	2,26	0,79														
1	1,62	1,04	0,25	0,29												
	4801,07	4966,46	1444,78	3505,16												
	1,41	0,57	1,60	0,65												
2	1,25	1,32	0,07	0,47	-0,02	0,29										
	70,78	235,86	92,77	369,64	1436,05	2038,43										
	0,61	0,29	0,64	0,36	0,75	0,49										
5	0,48	0,72	-0,04	0,27	-0,02	0,16	-0,07	0,19								
	0,04	0,05	0,00	0,07	0,03	0,09	2,32	19,65								
	0,28	0,20	0,15	0,12	0,14	0,10	0,45	0,28								
10	0,11	0,43	0,01	0,13	0,00	0,06	0,00	0,06	-0,10	0,17						
	0,01	0,03	0,00	0,04	0,01	0,04	0,01	0,07	5,06	13,49						
	0,27	0,20	0,03	0,03	0,03	0,02	0,09	0,07	0,61	0,44						
20	0,03	0,36	0,00	0,09	0,00	0,04	0,00	0,03	0,00	0,05	-0,13	0,17				
	0,00	0,03	0,00	0,03	0,00	0,04	0,01	0,06	0,05	0,12	3,66	5,62				
	0,24	0,19	0,03	0,03	0,01	0,01	0,03	0,02	0,12	0,09	0,67	0,51				
50	0,01	0,29	0,00	0,08	0,00	0,03	0,00	0,02	0,00	0,02	0,00	0,04	-0,18	0,17		
	0,03	0,09	0,02	0,09	0,03	0,10	0,08	0,23	0,62	1,66	1,96	2,25	2,42	3,87		
	0,20	0,16	0,04	0,04	0,02	0,02	0,02	0,03	0,07	0,12	0,22	0,15	0,83	0,55		
100	-0,01	0,27	0,00	0,06	0,00	0,03	0,00	0,02	0,00	0,02	-0,02	0,03	-0,12	0,12	-0,46	0,15
	0,99	1,35	0,98	1,47	1,30	1,71	2,05	2,26	2,57	1,93	1,92	1,57	2,07	2,91	-0,12	0,34
	0,31	0,22	0,18	0,16	0,19	0,18	0,27	0,24	0,34	0,21	0,34	0,19	0,79	0,42	2,84	0,96

k=0,005	0,2		0,5		1		2		5		10		20		50	
0,5	1,12	1,04														
	1401,90	2167,49														
	4,09	2,66														
1	1,68	1,14	0,24	0,47												
	505,63	875,56	548,09	883,42												
	2,82	2,21	3,16	2,25												
2	1,47	1,39	0,02	0,56	-0,31	0,31										
	97,62	218,54	100,04	266,11	162,06	333,02										
	1,51	1,62	1,24	1,45	2,55	2,67										
5	1,34	1,65	0,15	0,45	-0,03	0,30	-0,22	0,32								
	0,98	7,47	0,22	0,45	2,51	9,95	23,56	49,85								
	0,44	0,25	0,42	0,21	0,32	0,24	0,53	0,48								
10	0,67	1,03	0,01	0,31	0,02	0,15	-0,01	0,16	-0,21	0,23						
	0,06	0,08	0,03	0,09	0,06	0,12	0,16	0,48	8,73	13,69						
	0,34	0,29	0,17	0,16	0,08	0,07	0,24	0,15	0,78	0,73						
20	0,29	0,72	0,01	0,24	0,00	0,11	-0,01	0,08	-0,02	0,11	-0,23	0,24				
	0,04	0,08	0,03	0,09	0,04	0,12	0,04	0,14	1,63	3,75	1,71	2,62				
	0,36	0,27	0,11	0,11	0,02	0,02	0,07	0,06	0,28	0,21	0,98	0,96				
50	0,11	0,60	-0,02	0,18	0,00	0,07	0,00	0,05	-0,03	0,05	-0,10	0,11	-0,26	0,23		
	0,22	0,72	0,55	1,25	1,14	2,15	2,21	3,14	2,90	3,28	2,29	2,39	5,40	8,98		
	0,45	0,29	0,16	0,14	0,13	0,16	0,20	0,20	0,30	0,20	0,58	0,30	1,68	1,13		
100	-0,29	0,47	-0,05	0,16	0,00	0,07	-0,01	0,03	-0,03	0,05	-0,08	0,08	-0,30	0,22	-0,52	0,16
	0,01	0,56	1,81	1,88	3,32	2,68	2,94	2,53	3,12	2,78	3,85	3,19	3,02	3,86	0,40	0,72
	0,71	0,41	0,31	0,24	0,40	0,28	0,34	0,25	0,40	0,29	0,70	0,40	1,49	0,70	3,34	1,32

k=0,01	0,2		0,5		1		2		5		10		20		50	
0,5	1,69	1,51														
	1325,84	2576,14														
	5,86	4,41														
1	2,28	1,40	0,35	0,67												
	634,50	1476,26	654,50	905,40												
	4,53	3,45	4,35	3,59												
2	1,54	1,33	-0,02	0,44	-0,38	0,29										
	178,63	289,84	192,63	307,73	189,21	292,32										
	5,05	4,76	5,83	5,86	6,59	6,45										
5	2,45	2,24	0,44	0,84	-0,03	0,41	-0,25	0,34								
	19,11	53,28	21,24	45,92	19,18	42,37	55,17	81,20								
	0,61	0,30	0,66	0,28	0,55	0,25	1,09	1,53								
10	1,88	2,02	0,09	0,47	-0,06	0,34	-0,03	0,27	-0,27	0,26						
	1,40	6,59	0,18	0,57	1,22	6,84	10,41	17,82	15,97	27,37						
	0,36	0,31	0,41	0,27	0,23	0,17	0,26	0,20	2,36	2,75						
20	0,98	1,51	-0,05	0,39	-0,01	0,19	-0,02	0,15	-0,11	0,22	-0,29	0,24				
	0,37	1,50	0,31	2,29	0,48	2,17	2,07	5,38	9,17	11,27	5,94	7,03				
	0,44	0,34	0,30	0,26	0,06	0,08	0,15	0,10	0,48	0,39	2,73	2,68				
50	0,45	0,95	-0,03	0,33	-0,02	0,14	-0,02	0,09	-0,08	0,12	-0,27	0,23	-0,39	0,20		
	1,38	1,96	3,44	3,76	5,37	5,68	6,17	6,39	6,87	6,07	3,54	4,26	0,85	2,40		
	0,52	0,32	0,35	0,25	0,35	0,30	0,40	0,32	0,63	0,29	1,04	0,58	4,01	3,14		
100	-0,08	0,76	-0,12	0,26	-0,05	0,12	-0,05	0,08	-0,10	0,11	-0,21	0,16	-0,34	0,18	-0,46	0,17
	0,63	1,26	3,26	2,97	4,94	4,00	5,52	4,13	5,60	3,97	2,77	2,52	0,26	1,14	-0,19	0,41
	0,74	0,43	0,45	0,26	0,46	0,33	0,50	0,34	0,69	0,35	1,00	0,65	3,21	2,30	10,57	5,05

R=0.7

τ_1 × frame rate

k = 0,001 (τ_2 × frame rate)	0,2	0,5	1	2	5	10	20	50
0,5	0,48 0,45<br>1846,01 3715,97<br>1,76 0,91							
1	0,65 0,68<br>219,09 839,24<br>0,82 0,46	-0,06 0,31<br>318,16 1027,13<br>1,06 0,76						
2	0,60 0,81<br>0,24 0,30<br>0,43 0,19	-0,09 0,35<br>0,35 0,57<br>0,36 0,25	-0,23 0,28<br>2,45 6,31<br>0,62 0,41					
5	0,14 0,37<br>0,02 0,04<br>0,20 0,13	-0,01 0,12<br>0,01 0,05<br>0,03 0,03	0,00 0,07<br>0,01 0,06<br>0,09 0,07	-0,02 0,09<br>0,09 0,19<br>0,36 0,25				
10	0,01 0,24<br>0,00 0,02<br>0,15 0,12	0,00 0,05<br>0,00 0,02<br>0,01 0,01	0,00 0,03<br>0,00 0,03<br>0,02 0,02	0,00 0,03<br>0,01 0,05<br>0,06 0,04	-0,04 0,08<br>0,21 0,36<br>0,52 0,37			
20	-0,01 0,19<br>0,00 0,02<br>0,13 0,09	0,00 0,04<br>0,00 0,02<br>0,01 0,01	0,00 0,02<br>0,00 0,03<br>0,01 0,00	0,00 0,01<br>0,00 0,03<br>0,02 0,01	-0,01 0,02<br>0,00 0,08<br>0,08 0,06	-0,08 0,09<br>0,12 0,26<br>0,71 0,50		
50	-0,26 0,17<br>-0,10 0,05<br>0,29 0,15	0,00 0,03<br>0,01 0,05<br>0,02 0,01	0,00 0,01<br>0,01 0,06<br>0,01 0,01	0,00 0,01<br>0,03 0,08<br>0,01 0,01	0,00 0,01<br>0,08 0,26<br>0,02 0,03	-0,01 0,02<br>0,19 0,30<br>0,08 0,07	-0,13 0,12<br>-0,17 0,14<br>0,90 0,69	
100	-0,52 0,09<br>-0,45 0,02<br>0,68 0,10	0,00 0,03<br>0,34 0,61<br>0,12 0,12	0,00 0,01<br>0,46 0,73<br>0,12 0,13	0,00 0,01<br>0,67 1,08<br>0,16 0,16	0,00 0,01<br>0,85 1,02<br>0,19 0,17	-0,01 0,01<br>-0,02 0,26<br>0,06 0,05	-0,08 0,06<br>-0,47 0,08<br>0,57 0,41	-0,23 0,06<br>-0,44 0,05<br>5,23 1,75

k=0,002 (τ_2 × frame rate)	0,2	0,5	1	2	5	10	20	50
0,5	0,59 0,80<br>8171,46 11549,43<br>2,26 1,28							
1	1,18 0,69<br>4408,33 5253,39<br>1,28 0,66	0,20 0,41<br>2043,45 4241,61<br>1,74 0,89						
2	0,86 0,99<br>93,72 299,53<br>0,49 0,24	-0,06 0,38<br>130,77 351,41<br>0,44 0,27	-0,07 0,30<br>939,99 1390,53<br>0,79 0,51					
5	0,28 0,67<br>0,04 0,08<br>0,28 0,20	0,00 0,24<br>0,03 0,10<br>0,13 0,12	-0,02 0,13<br>0,05 0,13<br>0,17 0,11	-0,11 0,18<br>0,27 0,57<br>0,58 0,46				
10	0,14 0,43<br>0,02 0,04<br>0,25 0,18	-0,01 0,13<br>0,00 0,05<br>0,03 0,04	0,00 0,05<br>0,01 0,06<br>0,04 0,03	-0,01 0,05<br>0,02 0,10<br>0,10 0,08	-0,08 0,14<br>7,76 19,98<br>0,68 0,50			
20	0,03 0,25<br>0,01 0,04<br>0,17 0,14	0,01 0,08<br>0,01 0,04<br>0,02 0,02	0,00 0,04<br>0,00 0,05<br>0,01 0,01	0,00 0,03<br>0,01 0,07<br>0,03 0,02	-0,01 0,04<br>0,27 1,44<br>0,14 0,12	-0,13 0,16<br>4,54 6,56<br>0,88 0,68		
50	-0,06 0,25<br>0,01 0,11<br>0,20 0,15	0,00 0,06<br>0,07 0,15<br>0,05 0,04	0,00 0,03<br>0,14 0,61<br>0,04 0,06	0,00 0,02<br>0,21 0,66<br>0,05 0,07	0,00 0,02<br>1,19 2,24<br>0,15 0,17	-0,02 0,04<br>2,36 2,92<br>0,36 0,23	-0,21 0,19<br>2,90 4,21<br>1,27 0,85	
100	0,03 0,24<br>1,88 2,30<br>0,39 0,31	-0,01 0,05<br>2,14 2,76<br>0,31 0,29	-0,01 0,03<br>2,17 2,50<br>0,31 0,26	0,00 0,02<br>2,50 2,57<br>0,33 0,26	-0,01 0,02<br>2,50 2,33<br>0,35 0,25	-0,02 0,03<br>1,96 1,85<br>0,40 0,25	-0,18 0,14<br>2,40 2,98<br>1,29 0,60	-0,43 0,13<br>-0,13 0,36<br>3,69 1,17

k=0,005 (τ_2 × frame rate)	0,2	0,5	1	2	5	10	20	50
0,5	0,92 0,98<br>1954,94 3236,63<br>3,97 2,79							
1	1,52 1,17<br>618,59 1027,02<br>3,17 2,58	0,16 0,46<br>556,45 948,24<br>3,14 2,43						
2	1,12 1,15<br>111,08 213,61<br>1,82 2,63	-0,09 0,49<br>83,53 164,96<br>1,28 2,04	-0,38 0,28<br>132,65 234,59<br>2,99 3,04					
5	1,09 1,29<br>0,55 2,75<br>0,36 0,23	0,00 0,41<br>0,55 3,49<br>0,41 0,19	-0,09 0,28<br>3,88 17,40<br>0,28 0,20	-0,19 0,28<br>50,83 85,97<br>0,64 0,50				
10	0,39 0,98<br>0,06 0,13<br>0,34 0,27	-0,01 0,31<br>0,06 0,13<br>0,18 0,17	-0,01 0,13<br>0,05 0,15<br>0,09 0,06	-0,04 0,11<br>0,50 3,33<br>0,23 0,18	-0,30 0,24<br>6,08 11,30<br>1,13 0,93			
20	0,25 0,60<br>0,06 0,10<br>0,33 0,23	0,00 0,21<br>0,05 0,13<br>0,08 0,09	-0,01 0,10<br>0,04 0,15<br>0,03 0,02	0,00 0,07<br>0,13 0,34<br>0,07 0,06	-0,04 0,11<br>3,78 6,30<br>0,44 0,32	-0,31 0,24<br>1,15 2,24<br>1,91 1,61		
50	0,08 0,56<br>0,46 1,08<br>0,42 0,29	0,01 0,13<br>1,28 2,33<br>0,19 0,22	-0,01 0,07<br>2,11 3,11<br>0,22 0,23	0,00 0,04<br>3,40 3,86<br>0,32 0,30	-0,03 0,05<br>4,31 4,09<br>0,47 0,28	-0,11 0,11<br>2,27 2,33<br>0,79 0,43	-0,28 0,21<br>4,74 6,52<br>2,28 1,45	
100	-0,22 0,44<br>0,07 0,72<br>0,56 0,33	-0,03 0,12<br>2,71 2,24<br>0,40 0,28	-0,02 0,06<br>3,91 3,41<br>0,50 0,36	-0,02 0,04<br>3,69 3,20<br>0,46 0,32	-0,02 0,03<br>3,11 2,74<br>0,50 0,33	-0,11 0,11<br>3,74 2,98<br>1,02 0,49	-0,29 0,23<br>3,16 4,92<br>2,45 1,64	-0,49 0,18<br>0,28 0,75<br>4,23 1,69

k=0,01 (τ_2 × frame rate)	0,2	0,5	1	2	5	10	20	50
0,5	1,45 1,50<br>1044,51 2071,65<br>6,72 5,08							
1	2,04 1,60<br>663,78 898,56<br>5,35 4,53	0,25 0,60<br>552,69 877,79<br>4,50 3,80						
2	1,00 1,15<br>163,60 339,33<br>4,82 4,62	-0,12 0,55<br>116,65 276,51<br>5,33 5,57	-0,45 0,28<br>154,34 376,03<br>8,96 8,21					
5	1,66 1,70<br>20,02 48,50<br>0,78 1,58	0,05 0,60<br>15,94 43,57<br>0,56 0,24	-0,16 0,40<br>37,74 78,29<br>0,41 0,53	-0,29 0,31<br>71,36 104,71<br>1,85 2,47				
10	1,41 1,72<br>2,55 8,51<br>0,35 0,29	0,10 0,55<br>2,77 9,50<br>0,36 0,25	-0,02 0,28<br>4,08 10,99<br>0,19 0,13	-0,08 0,22<br>16,37 24,67<br>0,37 0,28	-0,34 0,26<br>20,11 28,01<br>2,81 3,05			
20	0,88 1,30<br>1,62 3,69<br>0,44 0,29	0,05 0,39<br>1,71 4,59<br>0,24 0,21	0,00 0,20<br>1,70 4,56<br>0,10 0,12	-0,04 0,14<br>4,41 8,78<br>0,25 0,21	-0,14 0,19<br>11,20 12,55<br>0,79 0,49	-0,35 0,24<br>4,35 6,17<br>3,56 3,07		
50	0,28 0,89<br>2,31 3,38<br>0,52 0,39	-0,10 0,30<br>4,36 5,27<br>0,48 0,35	-0,06 0,14<br>6,75 6,63<br>0,47 0,38	-0,04 0,09<br>7,76 7,94<br>0,52 0,40	-0,09 0,10<br>7,22 5,42<br>0,80 0,33	-0,25 0,19<br>3,81 4,27<br>1,47 0,85	-0,38 0,19<br>0,61 1,46<br>5,03 3,62	
100	-0,14 0,67<br>1,17 1,69<br>0,62 0,42	-0,12 0,23<br>4,16 3,33<br>0,43 0,31	-0,04 0,11<br>6,27 4,89<br>0,62 0,43	-0,06 0,08<br>5,75 4,19<br>0,58 0,39	-0,08 0,10<br>4,80 3,54<br>0,73 0,34	-0,25 0,19<br>1,99 2,33<br>1,42 1,14	-0,41 0,20<br>0,23 1,36<br>4,21 3,16	-0,48 0,15<br>-0,20 0,41<br>10,85 5,39

R=0.8

τ_1 × frame rate

Rows: τ_2 × frame rate

k = 0,001	0,2	0,5	1	2	5	10	20	50
0,5	0,36 0,48<br>2056,94 5148,88<br>1,80 1,22							
1	0,27 0,53<br>136,44 451,27<br>0,69 0,55	-0,10 0,33<br>563,76 1795,18<br>1,34 0,93						
2	0,26 0,63<br>0,35 1,06<br>0,39 0,15	-0,12 0,31<br>1,12 4,80<br>0,35 0,21	-0,27 0,25<br>3,67 9,65<br>1,22 1,04					
5	-0,07 0,34<br>0,00 0,06<br>0,23 0,17	-0,01 0,11<br>0,01 0,08<br>0,05 0,04	-0,01 0,06<br>0,02 0,10<br>0,14 0,09	-0,04 0,11<br>0,16 0,39<br>0,58 0,57				
10	-0,05 0,23<br>0,00 0,03<br>0,15 0,13	0,01 0,05<br>0,01 0,04<br>0,01 0,01	0,00 0,03<br>0,00 0,05<br>0,03 0,02	-0,01 0,02<br>0,00 0,08<br>0,08 0,06	-0,07 0,09<br>0,22 0,34<br>0,89 0,66			
20	0,00 0,15<br>0,00 0,03<br>0,09 0,07	0,00 0,04<br>0,01 0,04<br>0,01 0,01	0,00 0,02<br>0,00 0,04<br>0,01 0,01	0,00 0,01<br>0,01 0,05<br>0,02 0,02	0,00 0,02<br>0,06 0,15<br>0,10 0,08	-0,09 0,11<br>0,04 0,24<br>1,09 0,97		
50	-0,14 0,15<br>-0,10 0,07<br>0,17 0,11	0,00 0,03<br>0,04 0,09<br>0,03 0,02	0,00 0,01<br>0,05 0,14<br>0,03 0,03	0,00 0,01<br>0,06 0,19<br>0,03 0,03	0,00 0,01<br>0,28 0,69<br>0,06 0,09	-0,01 0,02<br>0,10 0,26<br>0,14 0,13	-0,18 0,13<br>-0,30 0,10<br>1,81 0,98	
100	-0,31 0,14<br>-0,43 0,08<br>0,44 0,14	0,00 0,03<br>0,68 1,00<br>0,19 0,19	0,00 0,01<br>1,14 1,26<br>0,24 0,20	0,00 0,01<br>1,65 1,82<br>0,33 0,29	0,00 0,01<br>1,23 1,18<br>0,28 0,21	-0,01 0,01<br>-0,29 0,18<br>0,06 0,06	-0,12 0,09<br>-0,57 0,07<br>1,18 0,74	-0,29 0,05<br>-0,44 0,07<br>6,67 2,18

Rows: τ_2 × frame rate

k=0,002	0,2	0,5	1	2	5	10	20	50
0,5	0,54 0,41<br>17559,43 24466,47<br>1,85 0,82							
1	0,69 1,01<br>4672,35 5829,63<br>1,58 1,55	0,18 0,37<br>1257,40 3037,41<br>2,57 1,57						
2	0,40 1,18<br>135,82 699,38<br>1,47 1,77	-0,06 0,35<br>263,65 728,40<br>0,47 0,37	-0,15 0,30<br>1363,95 1762,23<br>0,99 0,66					
5	0,04 0,43<br>0,03 0,10<br>0,29 0,20	-0,02 0,21<br>0,08 0,16<br>0,22 0,31	-0,03 0,13<br>0,11 0,23<br>0,23 0,19	-0,12 0,18<br>22,31 77,30<br>0,89 0,64				
10	0,01 0,38<br>0,02 0,07<br>0,24 0,17	-0,01 0,11<br>0,02 0,08<br>0,03 0,02	-0,01 0,05<br>0,02 0,09<br>0,06 0,04	-0,01 0,04<br>0,06 0,16<br>0,13 0,09	-0,16 0,19<br>26,74 45,04<br>1,31 1,06			
20	0,00 0,27<br>0,01 0,06<br>0,17 0,13	0,01 0,07<br>0,02 0,07<br>0,02 0,02	0,00 0,03<br>0,02 0,08<br>0,02 0,01	0,00 0,03<br>0,04 0,13<br>0,04 0,03	-0,02 0,04<br>0,95 3,56<br>0,27 0,23	-0,17 0,20<br>13,03 17,11<br>1,63 1,20		
50	0,03 0,22<br>0,58 1,67<br>0,22 0,20	-0,01 0,06<br>0,56 1,87<br>0,11 0,16	0,00 0,02<br>0,85 2,11<br>0,12 0,18	0,00 0,02<br>1,28 3,17<br>0,15 0,25	-0,01 0,02<br>3,06 3,85<br>0,32 0,30	-0,03 0,04<br>2,70 2,83<br>0,55 0,37	-0,26 0,21<br>3,27 6,16<br>2,32 1,39	
100	-0,05 0,20<br>2,87 3,43<br>0,48 0,40	-0,01 0,05<br>3,16 3,20<br>0,44 0,34	-0,01 0,02<br>3,12 3,39<br>0,43 0,38	0,00 0,01<br>3,63 3,28<br>0,49 0,37	-0,01 0,02<br>3,48 3,20<br>0,51 0,37	-0,03 0,03<br>2,58 2,12<br>0,67 0,34	-0,17 0,14<br>2,63 3,22<br>1,91 1,15	-0,40 0,14<br>-0,16 0,76<br>5,34 1,53

Rows: τ_2 × frame rate

k=0,005	0,2	0,5	1	2	5	10	20	50
0,5	0,93 1,03<br>994,08 1602,08<br>4,91 4,11							
1	1,10 0,99<br>584,37 1028,46<br>3,56 3,61	0,13 0,41<br>629,30 1219,55<br>3,85 3,33						
2	0,96 1,10<br>167,67 338,44<br>1,27 1,70	-0,07 0,45<br>204,26 348,65<br>1,15 1,84	-0,42 0,27<br>120,60 235,78<br>5,93 5,97					
5	0,80 1,09<br>4,71 17,53<br>0,37 0,23	-0,05 0,41<br>4,13 19,10<br>0,31 0,18	-0,14 0,24<br>19,95 45,54<br>0,26 0,22	-0,25 0,28<br>45,47 86,05<br>1,17 1,14				
10	0,35 0,79<br>0,11 0,19<br>0,32 0,24	0,01 0,26<br>0,13 0,26<br>0,13 0,10	0,00 0,14<br>0,95 5,34<br>0,15 0,11	-0,04 0,12<br>5,35 14,14<br>0,40 0,30	-0,34 0,26<br>9,91 16,50<br>2,48 1,98			
20	0,21 0,61<br>0,25 1,03<br>0,33 0,24	0,01 0,19<br>0,42 1,68<br>0,09 0,12	0,00 0,08<br>0,47 2,22<br>0,06 0,08	-0,02 0,08<br>1,30 3,92<br>0,17 0,17	-0,12 0,14<br>5,48 8,97<br>0,98 0,72	-0,33 0,20<br>1,29 2,51<br>3,36 2,28		
50	-0,10 0,49<br>0,90 1,77<br>0,49 0,32	-0,02 0,14<br>3,75 4,06<br>0,44 0,35	-0,01 0,06<br>4,61 5,29<br>0,44 0,41	-0,02 0,04<br>4,85 6,11<br>0,46 0,46	-0,05 0,06<br>5,15 4,09<br>0,76 0,40	-0,16 0,15<br>1,33 1,79<br>1,33 0,87	-0,29 0,21<br>4,87 7,51<br>3,88 2,36	
100	-0,32 0,38<br>0,42 1,20<br>0,52 0,34	-0,05 0,12<br>3,63 3,49<br>0,54 0,41	-0,03 0,05<br>4,04 3,13<br>0,55 0,35	-0,02 0,04<br>4,66 4,15<br>0,64 0,46	-0,04 0,05<br>3,78 2,98<br>0,70 0,43	-0,14 0,13<br>4,16 3,49<br>1,53 0,74	-0,27 0,18<br>2,74 3,95<br>3,78 2,51	-0,49 0,16<br>0,24 0,99<br>5,86 2,33

Rows: τ_2 × frame rate

k=0,01	0,2	0,5	1	2	5	10	20	50
0,5	1,78 1,62<br>1139,08 2342,40<br>9,26 7,04							
1	1,90 1,54<br>615,71 958,34<br>6,89 5,95	0,16 0,56<br>622,06 992,20<br>5,23 4,96						
2	1,32 1,48<br>230,63 399,98<br>4,30 5,29	0,25 0,57<br>185,84 287,72<br>4,49 4,08	-0,50 0,25<br>133,84 264,37<br>12,19 10,99					
5	1,23 1,43<br>29,04 57,40<br>0,97 2,48	-0,06 0,55<br>36,64 75,87<br>0,59 1,62	-0,29 0,36<br>54,10 98,41<br>0,82 1,97	-0,35 0,28<br>80,72 119,06<br>4,03 4,56				
10	0,97 1,41<br>5,53 12,61<br>0,46 0,27	-0,11 0,39<br>4,88 16,30<br>0,32 0,22	-0,08 0,26<br>15,07 27,99<br>0,24 0,21	-0,20 0,25<br>29,70 40,29<br>0,68 0,52	-0,43 0,27<br>20,15 26,67<br>4,94 4,14			
20	0,49 1,03<br>2,00 5,51<br>0,52 0,36	-0,02 0,33<br>3,61 7,42<br>0,26 0,23	-0,02 0,17<br>9,88 14,57<br>0,35 0,38	-0,08 0,14<br>12,41 17,07<br>0,59 0,39	-0,26 0,20<br>12,91 13,53<br>1,83 1,44	-0,44 0,22<br>4,82 7,01<br>5,81 4,28		
50	0,10 0,74<br>3,28 3,88<br>0,65 0,38	-0,11 0,23<br>7,89 6,36<br>0,58 0,42	-0,08 0,12<br>10,55 10,35<br>0,75 0,61	-0,05 0,09<br>11,97 9,28<br>0,93 0,54	-0,15 0,16<br>8,24 6,54<br>1,36 0,74	-0,31 0,22<br>3,54 4,05<br>2,69 1,92	-0,39 0,17<br>0,69 1,85<br>7,98 5,13	
100	-0,14 0,65<br>2,19 2,75<br>0,62 0,46	-0,12 0,23<br>6,10 5,09<br>0,62 0,51	-0,08 0,11<br>6,57 4,99<br>0,71 0,46	-0,07 0,08<br>7,49 6,08<br>0,85 0,54	-0,17 0,15<br>3,18 3,62<br>1,12 0,64	-0,29 0,20<br>1,44 2,11<br>3,02 2,49	-0,43 0,16<br>-0,17 0,96<br>6,62 4,60	-0,54 0,12<br>-0,12 0,42<br>13,09 6,43

R=0.9

τ_1 × frame rate

τ_2 × frame rate

k = 0,001	0,2	0,5	1	2	5	10	20	50
0,5	0,28 0,39<br>1395,24 3403,93<br>2,75 2,03							
1	0,15 0,43<br>718,77 2026,73<br>1,00 1,08	-0,13 0,25<br>886,37 2121,44<br>1,97 1,82						
2	0,13 0,45<br>2,04 8,63<br>0,42 0,19	-0,23 0,26<br>10,50 40,20<br>0,70 0,56	-0,37 0,26<br>3,37 13,43<br>4,18 2,76					
5	0,00 0,33<br>0,04 0,12<br>0,17 0,15	-0,03 0,10<br>0,01 0,14<br>0,10 0,09	-0,03 0,07<br>0,06 0,20<br>0,33 0,30	-0,09 0,12<br>0,66 1,24<br>1,64 1,44				
10	0,00 0,18<br>0,02 0,06<br>0,09 0,08	-0,01 0,05<br>0,01 0,08<br>0,03 0,02	-0,01 0,02<br>0,01 0,10<br>0,07 0,05	-0,01 0,03<br>0,03 0,16<br>0,17 0,16	-0,15 0,14<br>-0,01 0,28<br>2,76 2,06			
20	0,01 0,14<br>0,02 0,06<br>0,08 0,06	-0,01 0,03<br>0,01 0,08<br>0,01 0,01	0,00 0,02<br>0,02 0,09<br>0,02 0,02	0,00 0,01<br>0,05 0,12<br>0,04 0,03	0,00 0,02<br>0,30 0,47<br>0,24 0,25	-0,21 0,18<br>-0,24 0,12<br>3,86 2,33		
50	-0,03 0,15<br>0,08 0,44<br>0,14 0,12	0,00 0,03<br>0,46 1,15<br>0,11 0,15	0,00 0,01<br>0,78 1,61<br>0,14 0,20	0,00 0,01<br>1,15 2,01<br>0,18 0,24	0,00 0,01<br>1,10 1,50<br>0,24 0,20	-0,03 0,03<br>-0,21 0,20<br>0,50 0,58	-0,25 0,17<br>-0,44 0,08<br>5,10 2,25	
100	-0,15 0,12<br>-0,37 0,21<br>0,28 0,13	0,00 0,02<br>1,70 1,76<br>0,38 0,32	0,00 0,01<br>2,11 2,04<br>0,42 0,32	0,00 0,01<br>3,29 2,82<br>0,59 0,44	-0,01 0,01<br>0,62 0,89<br>0,22 0,17	-0,04 0,04<br>-0,60 0,10<br>0,46 0,72	-0,21 0,15<br>-0,71 0,04<br>4,53 2,08	-0,33 0,05<br>-0,46 0,07<br>13,19 3,98

τ_2 × frame rate

k=0,002	0,2	0,5	1	2	5	10	20	50
0,5	0,35 0,45<br>22210,97 27449,74<br>2,36 1,96							
1	0,36 1,38<br>7606,52 8382,10<br>1,95 2,01	0,13 0,22<br>597,10 1595,87<br>3,57 2,40						
2	0,37 0,93<br>636,25 1522,86<br>0,89 0,95	0,08 0,28<br>361,34 1446,83<br>1,43 0,97	-0,09 0,22<br>1348,75 1739,48<br>1,79 1,21					
5	0,16 0,50<br>0,26 0,64<br>0,75 1,04	-0,07 0,20<br>1,77 14,55<br>1,29 1,72	-0,06 0,13<br>12,49 50,58<br>0,61 0,58	-0,18 0,19<br>124,11 243,53<br>2,44 1,73				
10	0,01 0,39<br>0,05 0,15<br>0,21 0,16	-0,02 0,11<br>0,07 0,19<br>0,81 1,46	0,00 0,05<br>0,09 0,23<br>0,12 0,10	-0,01 0,04<br>1,73 11,27<br>0,33 0,30	-0,16 0,19<br>90,98 122,17<br>2,89 2,16			
20	-0,07 0,24<br>0,03 0,16<br>0,17 0,14	0,00 0,07<br>0,08 0,20<br>1,20 2,04	0,00 0,03<br>0,37 2,92<br>0,05 0,10	-0,01 0,03<br>0,42 2,62<br>0,11 0,12	-0,04 0,06<br>9,64 13,89<br>1,02 0,92	-0,18 0,18<br>20,91 30,41<br>3,40 2,23		
50	-0,01 0,22<br>4,48 6,12<br>0,52 0,49	0,00 0,05<br>5,05 6,47<br>1,03 1,28	-0,01 0,02<br>5,33 7,24<br>0,48 0,55	0,00 0,01<br>6,80 8,81<br>0,61 0,67	-0,02 0,02<br>7,22 7,56<br>0,82 0,60	-0,10 0,12<br>7,00 6,84<br>2,25 1,56	-0,23 0,19<br>3,59 6,23<br>6,89 3,58	
100	-0,06 0,20<br>7,49 6,59<br>0,93 0,68	-0,02 0,05<br>7,62 6,24<br>1,78 1,50	-0,01 0,02<br>9,36 6,44<br>1,10 0,69	-0,01 0,02<br>7,34 6,19<br>0,92 0,68	-0,02 0,02<br>5,68 4,88<br>0,90 0,58	-0,08 0,11<br>3,45 3,36<br>1,97 1,57	-0,22 0,16<br>2,02 3,82<br>6,28 3,43	-0,44 0,13<br>0,30 1,41<br>10,37 2,93

τ_2 × frame rate

k=0,005	0,2	0,5	1	2	5	10	20	50
0,5	0,84 1,17<br>924,18 1887,64<br>8,02 7,72							
1	0,72 1,07<br>917,64 1399,24<br>5,90 6,90	0,08 0,42<br>621,97 1107,71<br>5,74 5,36						
2	0,84 1,00<br>291,20 506,15<br>3,23 4,39	-0,02 0,39<br>341,68 503,04<br>2,89 3,51	-0,51 0,22<br>93,94 221,48<br>14,49 12,18					
5	0,13 0,72<br>7,02 21,06<br>0,43 0,23	-0,13 0,33<br>24,84 55,25<br>0,25 0,17	-0,30 0,27<br>49,33 86,76<br>1,44 2,07	-0,39 0,27<br>35,14 74,84<br>5,40 4,61				
10	0,20 0,68<br>3,05 8,39<br>0,40 0,28	-0,08 0,26<br>3,49 11,96<br>0,17 0,20	-0,12 0,18<br>9,91 22,07<br>0,53 0,43	-0,15 0,18<br>26,28 37,50<br>1,62 1,40	-0,44 0,24<br>4,24 10,82<br>8,52 5,47			
20	0,00 0,48<br>1,69 4,05<br>0,38 0,30	-0,04 0,16<br>4,70 10,15<br>0,26 0,41	-0,03 0,08<br>7,32 12,83<br>0,40 0,40	-0,05 0,07<br>9,81 15,64<br>0,74 0,55	-0,18 0,18<br>7,59 10,75<br>2,67 2,12	-0,40 0,21<br>0,92 2,44<br>8,68 5,35		
50	-0,08 0,47<br>4,05 4,94<br>0,74 0,56	-0,08 0,13<br>7,06 7,13<br>0,69 0,57	-0,03 0,06<br>10,39 8,34<br>0,97 0,68	-0,02 0,04<br>13,87 11,00<br>1,29 0,81	-0,09 0,13<br>6,90 6,87<br>1,86 1,20	-0,29 0,21<br>0,55 1,60<br>5,29 3,58	-0,26 0,20<br>3,71 5,76<br>8,44 4,60	
100	-0,18 0,41<br>1,95 2,69<br>0,61 0,44	-0,08 0,11<br>6,23 4,89<br>0,87 0,57	-0,04 0,06<br>7,08 5,41<br>0,92 0,56	-0,05 0,07<br>6,76 5,19<br>1,00 0,66	-0,11 0,11<br>3,00 3,32<br>1,75 1,24	-0,18 0,17<br>7,41 10,13<br>3,54 1,97	-0,21 0,17<br>2,04 3,10<br>8,89 5,14	-0,52 0,16<br>0,70 1,72<br>11,64 4,47

τ_2 × frame rate

k=0,01	0,2	0,5	1	2	5	10	20	50
0,5	1,08 1,36<br>723,18 1300,68<br>12,29 10,97							
1	1,63 1,65<br>565,04 966,80<br>13,58 13,58	0,28 0,57<br>758,98 1319,41<br>10,03 9,12						
2	1,49 1,59<br>220,93 418,44<br>9,73 12,40	0,22 0,63<br>301,22 464,42<br>8,69 9,60	-0,52 0,23<br>135,22 276,29<br>27,83 20,23					
5	0,37 1,04<br>43,32 85,39<br>3,53 8,91	-0,12 0,44<br>82,94 124,65<br>2,64 5,85	-0,39 0,28<br>83,74 124,24<br>6,23 9,63	-0,52 0,27<br>64,85 113,49<br>13,15 11,33				
10	0,21 0,84<br>24,94 36,02<br>1,07 3,01	-0,22 0,34<br>29,35 45,97<br>0,68 2,14	-0,25 0,24<br>41,95 61,88<br>1,64 3,00	-0,31 0,23<br>40,71 53,31<br>3,28 4,66	-0,46 0,23<br>16,32 28,59<br>14,26 10,35			
20	0,14 0,82<br>10,65 15,71<br>0,79 0,66	-0,13 0,30<br>21,62 25,06<br>0,84 0,70	-0,14 0,18<br>23,70 23,53<br>1,02 0,74	-0,15 0,18<br>26,97 25,96<br>1,65 1,43	-0,37 0,24<br>12,18 14,79<br>6,76 5,39	-0,40 0,20<br>2,82 5,41<br>14,92 9,90		
50	-0,17 0,65<br>7,23 7,97<br>0,89 0,59	-0,19 0,23<br>13,68 10,89<br>1,08 1,05	-0,14 0,16<br>18,52 15,43<br>1,46 0,95	-0,15 0,17<br>16,46 12,37<br>2,00 1,46	-0,34 0,22<br>7,47 8,03<br>4,85 3,55	-0,42 0,21<br>1,54 3,16<br>11,18 8,81	-0,44 0,17<br>0,31 1,57<br>16,72 9,91	
100	-0,14 0,55<br>3,68 4,99<br>1,04 2,50	-0,19 0,26<br>9,07 8,11<br>1,05 0,80	-0,16 0,18<br>10,09 7,43<br>1,65 3,25	-0,19 0,16<br>9,52 7,39<br>2,36 2,90	-0,31 0,22<br>3,44 4,10<br>5,74 5,14	-0,38 0,20<br>0,80 1,83<br>10,44 8,01	-0,43 0,16<br>-0,29 0,72<br>14,48 9,84	-0,55 0,13<br>-0,17 0,55<br>25,72 13,65
