# Supplementary material for: FRAP to Characterize Molecular Diffusion and Interaction in Various Membrane Environments
Source: PLoS One. 2016 Jul 7;11(7):e0158457. doi: 10.1371/journal.pone.0158457 (PMC4936743; doi:10.1371/journal.pone.0158457)
Supplement: S3 Table — Each cell displays, for a given pair of (τ1, τ2) values, (i) the normalized differences between the averages and the theoretical values (left): (< τ¯1 >—τ1) / τ1 (first line), (< τ¯2 >—τ2) / τ2 (second line), │< R¯ >—R│ / R + │(1 - < R¯ >)—(1—R)│ / (1 –R) (third line), and (ii) the associated normalized standard deviations (right): σ(τ¯1) / τ1 (first line), σ(τ¯2) / τ2 (second line) and σ(R¯) / R (third line). The solvable cases (in green) were arbitrarily defined as the cases where < τ¯1 >, < τ¯2 > and < R¯ > all differ by less than 20% from the theoretical values, and all normalized standard deviations are lower than 20%. (PDF) [file pone.0158457.s008.pdf]

S3 Table. Numerical values of the *in silico* studies of FRAPP experiments with two diffusing species (see the legend of S5 Fig for details). Each cell displays, for a given pair of (τ_1, τ_2) values, (i) the normalized differences between the averages and the theoretical values (left): $(<\bar{\tau}_1> - \tau_1) / \tau_1$ (first line), $(<\bar{\tau}_2> - \tau_2) / \tau_2$ (second line), $|<\bar{R}> - R| / R + |(1 - <\bar{R}>) - (1 - R)| / (1 - R)$ (third line), and (ii) the associated normalized standard deviations (right): $\sigma(\bar{\tau}_1) / \tau_1$ (first line), $\sigma(\bar{\tau}_2) / \tau_2$ (second line) and $\sigma(\bar{R}) / R$ (third line). The solvable cases (in green) were arbitrarily defined as the cases where $<\bar{\tau}_1>$, $<\bar{\tau}_2>$ and $<\bar{R}>$ all differ by less than 20% from the theoretical values, and all normalized standard deviations are lower than 20%.

R=0.1

τ_1 × frame rate

Rows: τ_2 × frame rate

k = 0,001	0,2	0,5	1	2	5	10	20	50
0,5	1,46 0,26<br>2815,38 3278,00<br>11,22 3,15							
1	3,90 0,34<br>2494,67 2593,08<br>10,22 2,06	0,81 0,21<br>2050,66 2415,58<br>9,15 1,81						
2	7,70 1,50<br>440,22 642,41<br>8,45 3,04	0,42 0,47<br>67,66 303,14<br>3,13 2,00	0,13 0,30<br>143,65 416,84<br>2,46 2,02					
5	18,39 4,48<br>76,06 152,50<br>7,26 3,27	0,02 0,20<br>0,00 0,00<br>0,68 0,71	0,01 0,07<br>0,00 0,00<br>0,03 0,02	0,00 0,09<br>0,00 0,01<br>0,12 0,09				
10	44,11 7,00<br>76,82 101,64<br>7,72 2,91	0,38 1,50<br>0,00 0,00<br>0,38 0,57	0,00 0,04<br>0,00 0,00<br>0,02 0,02	0,00 0,04<br>0,00 0,00<br>0,02 0,03	0,02 0,08<br>0,00 0,01<br>0,26 0,25			
20	64,68 18,91<br>0,22 1,10<br>2,03 1,27	10,82 9,42<br>0,00 0,00<br>1,18 0,97	0,00 0,03<br>0,00 0,00<br>0,02 0,01	0,00 0,02<br>0,00 0,00<br>0,01 0,00	0,54 0,60<br>-0,02 0,02<br>1,43 1,32	0,04 0,14<br>0,03 0,05<br>1,04 1,11		
50	116,25 51,28<br>0,08 0,20<br>1,43 0,89	-0,01 0,07<br>0,00 0,00<br>0,14 0,12	0,00 0,02<br>0,00 0,00<br>0,01 0,01	0,00 0,01<br>0,00 0,00<br>0,24 0,80	0,00 0,01<br>0,00 0,01<br>0,01 0,01	0,01 0,04<br>0,01 0,02<br>0,06 0,04	0,05 0,23<br>0,25 0,25<br>1,30 0,92	
100	188,18 67,60<br>0,09 0,18<br>1,25 0,89	-3,65 14,55<br>-0,01 0,03<br>0,18 0,29	0,00 0,01<br>0,00 0,01<br>0,01 0,01	0,00 0,01<br>0,00 0,01<br>0,01 0,00	0,00 0,01<br>0,00 0,01<br>0,00 0,00	0,00 0,03<br>0,01 0,04<br>0,01 0,01	0,04 0,14<br>0,22 0,28<br>0,21 0,16	-0,75 1,18<br>0,03 0,15<br>1,02 0,82

k=0,002	0,2	0,5	1	2	5	10	20	50
0,5	1,59 0,28<br>2856,18 3198,58<br>10,20 3,00							
1	3,86 0,42<br>2040,31 2101,99<br>10,48 2,33	0,85 0,24<br>1880,25 1710,06<br>10,09 2,12						
2	7,82 1,66<br>325,45 573,13<br>10,02 3,69	1,38 0,87<br>350,32 663,52<br>5,32 2,35	0,47 0,32<br>707,57 884,69<br>6,45 2,23					
5	18,84 4,68<br>75,03 142,90<br>7,91 3,29	0,33 0,88<br>5,10 37,41<br>1,19 1,22	0,03 0,14<br>0,00 0,01<br>0,09 0,17	0,00 0,17<br>0,01 0,02<br>0,34 0,45				
10	42,67 7,42<br>44,58 64,94<br>7,97 4,14	0,80 2,04<br>0,77 7,58<br>0,92 0,90	0,12 0,58<br>0,00 0,00<br>0,12 0,36	0,04 0,19<br>0,00 0,00<br>0,11 0,46	0,06 0,17<br>0,05 0,12<br>1,03 1,20			
20	66,66 20,02<br>1,54 4,00<br>2,77 2,46	5,18 6,31<br>0,00 0,00<br>0,85 0,66	0,00 0,05<br>0,00 0,00<br>0,03 0,03	0,01 0,03<br>0,00 0,00<br>0,01 0,01	0,39 0,53<br>-0,01 0,02<br>0,78 0,93	0,07 0,19<br>0,31 0,83<br>1,65 1,58		
50	136,52 46,19<br>0,27 0,58<br>1,75 1,35	0,35 3,21<br>0,00 0,00<br>0,41 0,56	0,00 0,06<br>0,00 0,01<br>0,03 0,06	0,00 0,02<br>0,00 0,01<br>0,10 0,50	0,00 0,03<br>0,00 0,01<br>0,01 0,01	0,02 0,08<br>0,02 0,05<br>0,12 0,09	0,07 0,31<br>0,61 0,83<br>1,80 1,21	
100	197,49 71,64<br>0,31 0,52<br>1,83 1,58	-1,42 18,43<br>0,00 0,03<br>0,34 0,45	0,00 0,03<br>0,00 0,01<br>0,03 0,02	0,00 0,02<br>0,00 0,02<br>0,01 0,01	0,00 0,02<br>0,01 0,03<br>0,01 0,00	0,01 0,05<br>0,06 0,10<br>0,03 0,02	0,04 0,20<br>0,56 0,59<br>0,30 0,45	-0,48 1,20<br>0,06 0,22<br>1,66 1,41

k=0,005	0,2	0,5	1	2	5	10	20	50
0,5	1,55 0,45<br>1585,54 2277,74<br>9,26 3,58							
1	3,75 0,63<br>763,69 1111,10<br>12,80 6,60	0,85 0,25<br>1245,97 1319,34<br>11,31 4,83						
2	7,61 1,67<br>267,73 479,79<br>13,24 6,62	2,12 0,76<br>450,07 525,41<br>9,02 3,86	0,65 0,36<br>561,40 680,20<br>9,44 3,74					
5	19,60 4,27<br>118,80 147,43<br>9,46 5,04	3,62 2,38<br>37,63 84,34<br>4,55 2,98	0,37 0,61<br>8,60 35,32<br>1,28 1,34	0,17 0,39<br>23,81 70,29<br>2,34 1,88				
10	42,57 8,66<br>39,52 49,16<br>9,11 4,66	6,58 4,71<br>16,57 33,33<br>3,40 2,43	0,29 0,74<br>0,00 0,01<br>0,39 0,84	0,06 0,26<br>0,00 0,01<br>0,11 0,22	0,11 0,28<br>9,36 20,10<br>2,55 1,86			
20	75,32 17,06<br>6,44 10,13<br>3,47 2,82	9,74 9,32<br>0,85 2,91<br>1,89 1,68	0,11 0,65<br>0,00 0,01<br>0,10 0,12	0,03 0,10<br>0,00 0,01<br>0,03 0,02	0,27 0,48<br>0,01 0,04<br>0,59 0,82	0,17 0,37<br>4,29 5,33<br>3,03 2,09		
50	137,81 81,73<br>0,49 0,68<br>3,43 2,91	7,31 17,64<br>0,10 0,29<br>1,00 0,77	-1,46 8,37<br>0,00 0,02<br>0,25 0,66	-0,04 0,14<br>0,00 0,03<br>0,25 0,66	0,02 0,08<br>0,01 0,04<br>0,05 0,03	0,11 0,27<br>0,47 0,91<br>0,41 0,41	0,06 0,37<br>1,54 1,96<br>1,88 1,60	
100	192,35 71,64<br>0,95 1,47<br>3,22 2,97	5,23 17,24<br>0,08 0,44<br>0,88 0,96	-0,25 2,57<br>0,01 0,04<br>0,10 0,31	0,01 0,05<br>0,01 0,04<br>0,03 0,02	-0,05 0,51<br>0,01 0,09<br>0,08 0,67	-0,02 0,44<br>0,52 1,17<br>0,16 0,43	-0,01 0,28<br>1,23 1,14<br>0,41 0,42	-0,27 0,57<br>0,40 0,60<br>3,17 3,21

k=0,01	0,2	0,5	1	2	5	10	20	50
0,5	1,65 0,77<br>1897,67 2203,86<br>14,59 12,56							
1	2,02 14,50<br>716,95 901,15<br>14,42 12,12	0,82 0,36<br>1082,02 955,71<br>13,72 9,77						
2	-26,58 346,17<br>410,69 489,18<br>15,81 9,60	2,35 0,66<br>335,59 387,69<br>12,54 8,20	0,76 0,28<br>434,81 510,50<br>12,69 7,89					
5	20,66 5,01<br>105,16 151,98<br>13,26 9,71	5,79 2,24<br>65,69 107,66<br>7,29 4,91	-2,97 43,50<br>52,06 107,71<br>4,88 4,22	0,50 0,43<br>64,91 108,10<br>5,64 3,38				
10	42,64 8,93<br>41,31 54,77<br>9,93 5,96	11,89 4,27<br>33,57 40,90<br>5,14 3,58	1,48 1,85<br>8,53 23,41<br>1,92 2,04	0,36 0,52<br>1,62 7,52<br>0,89 2,08	0,23 0,37<br>23,09 30,66<br>3,93 2,82			
20	74,08 18,41<br>9,06 10,69<br>3,89 3,32	18,51 9,67<br>4,25 5,55<br>2,23 1,95	1,41 2,80<br>0,79 2,71<br>0,85 1,85	0,11 0,23<br>0,01 0,02<br>0,08 0,08	0,26 0,48<br>1,00 2,81<br>0,86 0,85	0,34 0,36<br>12,85 12,09<br>3,51 2,49		
50	146,04 47,61<br>1,52 1,78<br>5,75 5,74	27,24 17,50<br>1,03 1,47<br>2,73 2,59	0,12 0,76<br>0,04 0,38<br>0,25 0,47	-0,03 0,19<br>0,00 0,04<br>0,45 1,91	0,06 0,20<br>0,06 0,17<br>0,16 0,42	0,25 0,42<br>2,50 3,03<br>0,40 0,33	0,31 0,42<br>3,27 3,18<br>2,94 2,97	
100	168,58 99,81<br>1,76 2,10<br>4,15 3,94	20,40 36,19<br>1,04 1,61<br>2,12 2,39	-0,38 4,14<br>0,01 0,06<br>0,20 0,25	0,06 1,78<br>0,00 0,10<br>0,24 0,80	0,04 0,12<br>0,26 0,69<br>0,06 0,07	0,05 0,29<br>1,80 2,29<br>0,60 1,19	-0,17 0,78<br>1,99 2,17<br>1,38 1,93	-0,24 0,34<br>1,84 2,00<br>4,78 5,08

R=0.1

τ_2 × frame rate

k=0,02	0,2	0,5	1	2	5	10	20	50
0,5	2,25 1,73<br>1795,49 2353,40<br>31,71 42,13							
1	0,73 21,15<br>761,23 925,20<br>16,42 12,62	-2,11 13,13<br>727,75 874,96<br>19,34 16,55						
2	-294,10 1750,93<br>289,09 384,17<br>18,58 13,44	-16,19 183,46<br>325,60 399,19<br>17,27 13,25	-32,01 229,30<br>461,22 479,27<br>16,82 12,41					
5	-6,58 274,32<br>107,61 136,72<br>18,39 14,02	6,95 1,97<br>110,11 128,29<br>13,02 9,71	2,41 1,11<br>68,48 105,66<br>9,85 8,52	0,72 0,43<br>88,92 119,92<br>8,78 7,64				
10	42,67 9,07<br>41,68 43,45<br>12,99 10,84	14,30 4,55<br>29,98 36,32<br>8,59 6,81	4,19 2,41<br>26,11 31,88<br>5,03 5,75	0,12 8,44<br>11,02 21,33<br>2,58 2,14	0,41 0,34<br>37,25 43,44<br>6,95 6,26			
20	76,68 17,45<br>8,21 9,32<br>7,00 6,89	23,96 8,66<br>8,29 9,84<br>7,25 7,63	4,85 4,28<br>2,77 5,29<br>4,05 6,11	1,08 1,24<br>2,68 5,00<br>0,90 1,64	0,55 0,60<br>5,74 6,96<br>1,94 2,52	0,31 0,32<br>11,39 10,93<br>4,44 4,54		
50	143,49 48,21<br>3,77 3,73<br>9,16 10,30	36,29 19,52<br>2,50 3,12<br>6,14 6,31	4,10 8,00<br>1,58 3,44<br>1,95 2,83	0,15 0,48<br>0,12 0,54<br>0,36 0,85	0,11 0,39<br>1,28 2,54<br>0,24 0,49	0,40 0,55<br>5,88 5,18<br>0,85 1,30	0,23 0,40<br>4,62 3,91<br>5,20 6,39	
100	147,33 104,25<br>2,51 2,61<br>7,13 5,93	32,80 22,39<br>3,19 3,37<br>4,76 4,91	0,81 5,59<br>0,32 1,15<br>1,37 3,75	0,18 1,98<br>0,23 0,69<br>0,57 1,38	0,02 0,22<br>2,09 3,28<br>0,24 0,35	0,05 0,36<br>3,79 2,99<br>0,50 0,64	0,07 0,47<br>3,35 3,51<br>4,15 5,30	-0,46 0,71<br>4,32 5,29<br>6,08 4,72

τ_2 × frame rate

k=0,05	0,2	0,5	1	2	5	10	20	50
0,5	-168871,3 661284,8<br>-36335,5 147843,1<br>738,2 2441,9							
1	-73061,9 471201,8<br>-10486,4 72608,2<br>1357,5 8946,4	-5363,09 53365,69<br>-1092,72 18488,61<br>251,75 2090,03						
2	-168,7 1032,0<br>378,0 341,4<br>29,3 27,9	-20,83 233,99<br>330,73 354,14<br>32,41 29,95	-14,72 114,24<br>414,42 383,87<br>31,13 28,88					
5	22,2 5,3<br>103,5 109,7<br>26,8 23,3	7,59 2,37<br>97,12 117,47<br>24,28 21,38	3,01 1,00<br>97,89 115,67<br>25,83 23,14	1,05 0,54<br>98,53 108,10<br>21,61 23,35				
10	42,4 9,1<br>42,0 42,8<br>16,5 18,0	15,30 4,09<br>42,28 41,28<br>17,37 18,62	5,68 2,03<br>30,78 31,65<br>10,31 12,50	2,13 1,03<br>36,41 40,67<br>10,31 14,81	0,58 0,33<br>43,20 41,53<br>13,38 13,65			
20	75,7 15,2<br>12,6 12,0<br>10,4 11,0	24,72 7,97<br>10,82 12,98<br>10,59 12,50	10,42 4,18<br>11,10 13,30<br>6,94 8,68	3,53 1,93<br>9,73 11,83<br>4,55 7,22	1,16 0,64<br>15,74 14,23<br>3,78 4,71	0,38 0,29<br>15,52 13,33<br>5,95 7,08		
50	113,1 48,4<br>7,3 7,9<br>14,9 13,3	37,55 16,96<br>7,70 7,18<br>15,63 13,87	11,54 7,09<br>5,78 7,68<br>11,15 12,93	2,66 2,49<br>5,46 8,30<br>4,11 7,12	0,74 0,82<br>8,84 8,97<br>3,96 7,79	0,53 0,59<br>11,14 8,35<br>6,94 8,67	0,08 0,43<br>9,67 7,99<br>12,98 16,85	
100	166,0 271,6<br>3,3 3,8<br>16,3 14,8	45,08 87,42<br>4,71 5,08<br>13,39 17,70	11,21 12,14<br>5,49 7,13<br>9,39 13,97	1,89 4,53<br>7,88 9,01<br>4,58 9,51	0,21 0,87<br>11,34 10,29<br>1,56 4,15	0,07 0,74<br>10,66 8,61<br>4,72 8,25	0,09 0,59<br>8,59 9,98<br>10,86 12,86	-0,51 0,25<br>10,69 10,95<br>11,72 11,01

R=0.2

τ_1 × frame rate

(τ_2 × frame rate down the rows)

k = 0,001	0,2		0,5		1		2		5		10		20		50	
0,5	1,46	0,20														
	5939,61	5839,80														
	5,17	0,78														
1	3,08	0,92	0,61	0,29												
	78,11	320,69	1353,60	1749,55												
	2,33	1,25	3,87	1,07												
2	7,74	1,47	0,07	0,28	0,02	0,18										
	571,27	777,18	0,01	0,01	0,04	0,14										
	4,16	1,65	0,58	0,50	0,47	0,47										
5	18,24	4,36	0,02	0,11	0,00	0,04	0,01	0,05								
	198,45	266,46	0,00	0,00	0,00	0,00	0,00	0,01								
	2,80	1,49	1,51	1,05	0,03	0,12	1,98	1,00								
10	39,09	9,20	2,35	3,38	0,00	0,02	0,00	0,01	-0,01	0,10						
	65,60	81,02	0,00	0,00	0,00	0,00	0,00	0,00	0,00	0,01						
	2,66	1,40	1,20	1,52	0,01	0,01	0,01	0,01	0,16	0,17						
20	54,46	24,03	0,00	0,05	0,00	0,01	0,00	0,01	0,00	0,01	0,00	0,08				
	1,18	4,21	0,00	0,00	0,00	0,00	0,00	0,00	0,00	0,00	0,01	0,03				
	1,12	0,80	0,08	0,07	0,01	0,01	0,00	0,00	0,02	0,01	0,28	0,22				
50	84,28	48,16	0,01	0,04	0,00	0,01	0,00	0,01	0,00	0,01	0,00	0,02	0,00	0,15		
	0,05	0,09	0,00	0,00	0,00	0,00	0,00	0,00	0,00	0,01	0,01	0,03	0,17	0,25		
	0,86	0,56	0,07	0,06	0,01	0,01	0,00	0,00	0,00	0,00	0,03	0,03	0,51	0,38		
100	130,14	77,14	0,00	0,04	-2,47	6,91	0,00	0,01	0,00	0,03	0,00	0,01	0,00	0,07	-0,55	1,23
	0,11	0,15	0,00	0,01	-0,08	0,10	0,00	0,01	0,00	0,03	0,01	0,05	0,18	0,26	-0,04	0,13
	1,04	0,34	0,07	0,07	1,14	2,59	0,01	0,00	0,01	0,01	0,01	0,00	0,09	0,08	0,67	0,47

k=0,002	0,2		0,5		1		2		5		10		20		50	
0,5	1,43	0,30														
	4355,01	4037,51														
	5,30	1,40														
1	3,03	0,92	0,69	0,27												
	250,88	625,80	1418,45	1718,28												
	3,83	2,39	4,67	1,51												
2	8,21	1,41	0,37	0,56	0,13	0,27										
	475,12	577,67	32,47	148,96	104,49	368,51										
	5,42	2,00	1,38	0,86	1,62	1,13										
5	19,03	4,77	0,03	0,20	0,00	0,08	0,01	0,08								
	138,74	188,09	0,00	0,00	0,00	0,01	0,00	0,01								
	4,46	2,27	1,23	0,97	0,07	0,21	1,43	1,00								
10	41,04	8,50	1,00	2,44	0,00	0,04	0,00	0,03	-0,01	0,15						
	41,74	65,65	0,00	0,00	0,00	0,00	0,00	0,00	0,01	0,03						
	3,26	1,96	0,69	1,02	0,02	0,02	0,01	0,01	0,45	0,38						
20	28,67	317,95	-0,01	0,11	0,01	0,03	0,00	0,02	0,00	0,03	0,04	0,14				
	1,48	4,24	0,00	0,00	0,00	0,00	0,00	0,00	0,00	0,01	0,16	0,43				
	1,73	1,25	0,25	0,25	0,02	0,01	0,01	0,00	0,03	0,02	0,68	0,57				
50	112,78	48,02	0,00	0,08	0,00	0,04	0,00	0,01	0,00	0,02	0,00	0,05	-0,02	0,21		
	0,17	0,28	0,00	0,00	0,00	0,01	0,00	0,01	0,00	0,02	0,02	0,06	0,43	0,66		
	1,25	0,84	0,14	0,12	0,02	0,08	0,00	0,00	0,01	0,01	0,07	0,04	0,59	0,45		
100	146,48	78,34	0,01	0,08	-3,27	8,46	0,00	0,01	0,00	0,01	0,00	0,03	0,01	0,11	-0,56	1,43
	0,20	0,33	0,00	0,01	-0,12	0,12	0,00	0,02	0,00	0,04	0,05	0,12	0,61	0,66	0,07	0,27
	1,55	1,39	0,13	0,12	1,61	3,30	0,01	0,01	0,01	0,01	0,02	0,02	0,18	0,18	0,96	0,76

k=0,005	0,2		0,5		1		2		5		10		20		50	
0,5	1,55	0,53														
	2911,27	2765,43														
	6,31	3,73														
1	3,51	0,79	0,68	0,30												
	759,38	1023,44	1262,17	1296,48												
	6,46	4,43	4,69	2,07												
2	2,82	51,42	1,41	0,87	0,43	0,30										
	391,23	458,76	241,23	456,12	332,58	479,36										
	6,12	3,57	3,15	1,54	3,69	1,59										
5	18,47	4,95	0,90	1,19	0,05	0,25	0,05	0,23								
	114,22	150,49	6,94	34,74	0,01	0,02	0,07	0,23								
	5,99	3,85	1,30	0,97	0,15	0,20	0,98	0,73								
10	39,93	9,33	1,02	2,34	0,01	0,11	0,00	0,08	0,02	0,19						
	39,92	54,63	2,01	9,89	0,00	0,01	0,00	0,01	1,70	8,32						
	4,06	2,76	0,89	0,76	0,06	0,05	0,05	0,03	0,78	0,67						
20	73,03	18,94	0,16	1,15	-0,01	0,06	0,00	0,05	0,01	0,07	0,00	0,25				
	8,76	12,12	0,00	0,01	0,00	0,01	0,00	0,01	0,01	0,03	2,79	3,88				
	2,45	1,72	0,69	0,57	0,04	0,04	0,02	0,01	0,09	0,08	1,04	0,78				
50	111,39	49,84	0,02	0,21	-0,06	0,15	0,00	0,05	-0,01	0,07	0,02	0,11	0,01	0,26		
	0,49	0,74	0,00	0,01	-0,01	0,03	0,00	0,02	0,00	0,04	0,19	0,47	2,17	2,08		
	1,95	1,56	0,57	0,46	0,17	0,33	0,01	0,03	0,04	0,15	0,13	0,10	0,64	0,53		
100	146,69	72,10	0,00	0,20	-1,28	5,12	0,00	0,03	0,00	0,03	-0,02	0,31	-0,05	0,20	-0,30	0,56
	0,90	1,20	0,00	0,03	-0,05	0,09	0,00	0,04	0,03	0,11	0,56	0,89	1,41	1,44	0,30	0,63
	1,87	1,27	0,48	0,41	0,62	1,96	0,02	0,02	0,03	0,03	0,15	0,26	0,50	0,49	2,18	1,85

k=0,01	0,2		0,5		1		2		5		10		20		50	
0,5	1,59	0,82														
	1801,25	2659,95														
	9,37	10,99														
1	3,64	0,86	0,71	0,42												
	586,78	882,13	607,75	832,60												
	8,02	6,15	7,49	5,29												
2	8,29	1,66	2,04	0,72	0,50	0,34										
	397,15	480,79	300,80	439,34	342,76	450,88										
	8,06	6,89	5,32	3,26	5,43	3,71										
5	18,95	5,37	3,94	2,27	0,39	0,61	0,20	0,31								
	83,40	114,05	62,94	108,11	11,24	39,11	22,21	54,49								
	6,83	4,91	2,73	2,61	0,89	0,87	1,43	0,91								
10	42,64	8,47	6,38	4,22	0,07	0,31	0,06	0,18	0,08	0,27						
	33,58	40,14	16,25	29,67	0,01	0,04	0,01	0,03	13,08	21,02						
	5,12	3,84	1,65	1,11	0,19	0,26	0,12	0,14	1,22	0,94						
20	73,38	19,35	6,49	7,60	0,04	0,15	0,01	0,09	0,03	0,15	0,04	0,29				
	9,06	9,93	1,21	3,01	0,00	0,01	0,00	0,02	0,03	0,07	7,36	7,56				
	2,56	2,28	1,26	1,26	0,10	0,09	0,03	0,02	0,20	0,18	1,06	0,78				
50	109,77	44,11	6,34	20,94	-0,03	0,16	0,01	0,06	-0,05	0,17	0,05	0,20	-0,02	0,30		
	1,80	2,25	0,43	1,05	-0,01	0,04	0,00	0,04	0,01	0,12	1,53	2,03	3,36	2,96		
	2,91	3,76	1,07	1,01	0,18	0,22	0,02	0,02	0,18	0,31	0,19	0,13	0,97	1,13		
100	149,60	76,84	3,86	17,66	-0,52	3,21	-0,24	1,62	-0,29	1,23	-0,01	0,11	-0,19	0,22	-0,43	0,82
	1,14	1,55	0,15	0,69	-0,01	0,12	0,02	0,12	0,20	0,65	2,00	1,91	1,84	1,62	1,24	1,79
	3,47	3,12	0,94	0,89	0,32	0,97	0,36	2,04	0,52	1,93	0,32	0,20	0,63	0,25	3,17	2,61

R=0.2

τ_2 × frame rate

k=0,02	0,2	0,5	1	2	5	10	20	50
0,5	2,36 1,70<br>1831,48 2493,30<br>17,73 27,61							
1	0,33 23,18<br>794,81 1047,06<br>10,49 10,88	0,22 6,21<br>923,13 1070,10<br>9,72 9,33						
2	-104,04 813,92<br>334,90 322,04<br>10,04 10,42	2,05 1,71<br>292,53 374,56<br>7,05 6,51	-30,24 216,85<br>371,70 393,33<br>7,47 6,43					
5	20,43 4,91<br>104,71 118,38<br>7,85 7,50	5,65 1,98<br>104,12 132,88<br>5,36 6,07	1,16 0,95<br>39,42 81,65<br>2,46 2,27	0,47 0,40<br>84,55 97,95<br>3,12 3,08				
10	42,81 8,45<br>40,06 44,99<br>6,77 5,59	10,85 4,81<br>23,99 32,97<br>3,07 3,43	1,14 1,46<br>9,74 21,16<br>0,84 0,74	0,24 0,49<br>3,67 13,36<br>0,53 0,53	0,18 0,30<br>22,94 31,11<br>2,04 2,17			
20	73,17 19,64<br>7,52 9,20<br>4,32 4,40	17,06 9,40<br>6,39 8,22<br>2,24 3,17	0,73 1,55<br>0,46 1,86<br>0,68 1,44	0,17 0,46<br>0,11 0,82<br>0,13 0,17	0,08 0,28<br>1,54 3,74<br>0,35 0,34	0,11 0,34<br>10,67 9,46<br>2,01 2,38		
50	106,60 48,88<br>2,55 3,16<br>5,35 5,43	20,71 14,86<br>2,24 2,86<br>2,41 2,63	0,00 3,24<br>0,12 0,81<br>0,53 1,20	0,02 0,16<br>0,04 0,09<br>0,07 0,09	-0,06 0,73<br>0,53 1,68<br>0,26 0,68	0,08 0,25<br>5,69 4,60<br>0,43 0,40	0,00 0,31<br>5,10 4,48<br>2,50 2,78	
100	108,20 52,53<br>2,41 2,43<br>4,37 3,44	17,95 20,88<br>1,29 2,12<br>1,99 2,65	-0,98 5,14<br>0,14 0,57<br>0,73 2,05	0,04 0,13<br>0,47 1,30<br>0,14 0,12	-0,13 0,81<br>1,30 1,93<br>0,36 1,20	-0,02 0,17<br>3,96 3,23<br>0,48 0,24	-0,29 1,12<br>2,78 2,71<br>1,25 1,46	-0,41 0,23<br>3,16 4,16<br>4,11 2,89

τ_2 × frame rate

k=0,05	0,2	0,5	1	2	5	10	20	50
0,5	-90592,50 437086,91<br>-50534,72 239962,74<br>516,95 1691,71							
1	-46,07 487,88<br>789,87 989,49<br>22,42 24,85	-8620,34 60312,29<br>-1876,67 20109,00<br>211,65 1348,82						
2	9,00 2,63<br>331,00 345,58<br>20,48 18,15	-24,29 269,02<br>271,61 323,42<br>20,07 19,09	-10,61 113,43<br>380,17 398,76<br>14,89 16,63					
5	22,16 5,98<br>108,24 121,55<br>17,40 14,35	7,25 2,28<br>109,44 104,47<br>13,38 13,26	2,44 1,07<br>96,02 121,88<br>8,18 10,30	0,82 0,41<br>99,92 124,67<br>11,40 11,93				
10	43,69 9,98<br>42,03 47,27<br>8,99 9,15	13,64 4,36<br>30,92 38,24<br>7,63 9,35	4,46 2,26<br>25,07 37,94<br>4,90 6,60	1,13 0,90<br>28,14 42,36<br>2,39 3,97	0,35 0,37<br>37,37 45,56<br>5,54 7,08			
20	70,69 18,80<br>10,29 12,29<br>6,51 6,87	22,89 8,92<br>10,98 11,25<br>6,34 11,32	6,00 3,51<br>7,92 10,59<br>2,33 3,89	1,18 1,13<br>3,70 6,81<br>0,66 1,25	0,55 0,47<br>12,74 13,82<br>0,83 1,43	0,28 0,32<br>12,37 14,25<br>2,43 3,54		
50	83,74 39,97<br>6,37 6,19<br>9,23 8,42	27,71 15,13<br>6,43 7,52<br>7,15 8,46	5,03 5,92<br>3,19 5,53<br>3,71 7,51	0,37 1,02<br>1,82 3,67<br>0,52 1,23	0,11 0,35<br>7,52 8,87<br>0,38 0,37	0,10 0,35<br>11,37 8,30<br>2,05 3,44	-0,03 0,32<br>8,99 7,49<br>5,86 6,20	
100	188,77 301,88<br>2,60 2,81<br>10,23 9,39	39,92 66,24<br>2,92 3,92<br>8,40 8,98	3,68 5,71<br>4,32 5,25<br>3,00 4,89	-293,34 2918,28<br>-0,11 50,64<br>155,77 1541,88	-0,11 0,62<br>6,80 6,40<br>0,61 1,50	-0,08 0,30<br>7,00 5,17<br>1,38 2,65	-0,23 0,68<br>6,61 6,02<br>5,40 6,70	-0,53 0,22<br>9,90 10,86<br>6,97 5,89

R=0.3

τ_1 × frame rate

τ_2 × frame rate

k = 0,001	0,2		0,5		1		2		5		10		20		50	
0,5	1,45	0,24														
	4504,96	4392,81														
	3,70	0,85														
1	-249,04	2513,85	0,15	0,33												
	2650,82	2485,29	206,15	722,55												
	3,30	0,85	1,33	0,73												
2	7,65	1,68	0,01	0,18	0,01	0,12										
	1059,18	1049,92	0,00	0,01	0,02	0,04										
	2,72	1,04	0,31	0,31	0,27	0,24										
5	15,70	5,60	0,01	0,06	0,00	0,02	0,00	0,03								
	194,80	255,26	0,00	0,00	0,00	0,00	0,00	0,01								
	1,68	1,16	0,10	0,08	0,01	0,01	0,05	0,04								
10	32,13	11,26	0,25	1,20	0,00	0,01	0,00	0,01	0,00	0,03						
	33,95	52,01	0,00	0,00	0,00	0,00	0,00	0,00	0,00	0,01						
	1,28	0,99	0,11	0,17	0,01	0,00	0,01	0,00	0,08	0,06						
20	36,92	23,67	0,00	0,03	0,00	0,01	0,00	0,01	0,00	0,01	0,00	0,05				
	2,17	7,36	0,00	0,00	0,00	0,00	0,00	0,00	0,00	0,01	0,01	0,04				
	1,10	0,72	0,06	0,04	0,01	0,00	0,00	0,00	0,01	0,01	0,19	0,16				
50	52,04	47,25	-8,91	24,15	0,00	0,01	0,00	0,00	-0,89	2,38	0,00	0,02	0,00	0,11		
	0,05	0,10	-0,02	0,04	0,00	0,00	0,00	0,00	-0,04	0,09	0,00	0,03	0,22	0,27		
	0,95	0,44	0,26	0,50	0,01	0,00	0,00	0,00	0,73	1,91	0,02	0,02	0,32	0,26		
100	93,03	68,24	0,00	0,03	0,00	0,01	0,00	0,00	0,00	0,00	0,00	0,01	0,00	0,06	-0,27	0,21
	0,11	0,20	0,00	0,01	0,00	0,01	0,00	0,01	0,00	0,02	0,03	0,07	0,22	0,37	-0,07	0,12
	1,23	0,32	0,06	0,04	0,01	0,01	0,01	0,00	0,02	0,06	0,02	0,01	0,14	0,30	0,64	0,38

τ_2 × frame rate

k=0,002	0,2		0,5		1		2		5		10		20		50	
0,5	1,42	0,34														
	3372,82	3581,27														
	3,83	1,54														
1	3,70	0,58	0,29	0,40												
	2336,82	2253,22	218,86	656,95												
	3,99	1,68	2,07	1,22												
2	-99,90	1039,89	0,13	0,41	0,05	0,24										
	741,10	830,42	1,81	17,06	15,79	112,25										
	3,34	1,76	0,74	0,53	0,79	0,55										
5	18,11	4,85	0,01	0,13	0,00	0,04	0,00	0,06								
	169,98	211,07	0,00	0,00	0,00	0,01	0,00	0,02								
	2,31	1,53	0,26	0,22	0,02	0,01	0,11	0,11								
10	36,33	10,95	0,61	1,84	0,00	0,03	-0,01	0,06	0,00	0,05						
	62,57	82,72	0,00	0,00	0,00	0,00	0,00	0,01	0,01	0,02						
	1,70	1,23	0,26	0,33	0,02	0,01	0,03	0,09	0,16	0,13						
20	53,20	23,90	0,01	0,09	0,00	0,02	0,00	0,01	0,00	0,02	0,01	0,09				
	1,79	5,46	0,00	0,00	0,00	0,00	0,00	0,00	0,00	0,01	0,07	0,13				
	1,37	1,09	0,15	0,14	0,01	0,01	0,00	0,00	0,02	0,02	0,37	0,29				
50	90,86	41,81	-2,20	12,58	0,00	0,02	0,00	0,01	-1,08	2,84	0,00	0,03	-0,05	0,14		
	0,16	0,23	0,00	0,02	0,00	0,01	0,00	0,01	-0,05	0,11	0,01	0,06	0,35	0,54		
	1,24	0,72	0,20	0,28	0,01	0,01	0,00	0,00	0,95	2,34	0,05	0,03	0,38	0,27		
100	146,57	70,56	0,01	0,07	-0,21	2,08	0,00	0,01	0,00	0,01	0,00	0,02	0,00	0,09	-0,21	0,24
	0,32	0,47	0,00	0,02	0,00	0,03	0,00	0,02	0,01	0,04	0,06	0,16	0,62	0,68	-0,01	0,19
	1,87	2,96	0,12	0,10	0,08	0,59	0,01	0,01	0,03	0,10	0,03	0,04	0,36	1,18	0,73	0,57

τ_2 × frame rate

k=0,005	0,2		0,5		1		2		5		10		20		50	
0,5	1,54	0,56														
	2548,08	2763,17														
	5,11	3,44														
1	3,80	0,59	0,54	0,36												
	1153,26	1181,50	651,83	1032,34												
	4,59	2,71	3,50	2,27												
2	-68,62	754,28	0,90	0,71	0,19	0,31										
	368,25	414,36	130,35	277,96	216,32	389,31										
	4,32	3,14	1,63	0,86	1,91	0,75										
5	19,53	4,62	0,38	0,87	0,00	0,13	0,01	0,12								
	147,75	179,78	9,46	50,84	0,00	0,02	0,03	0,07								
	3,84	2,74	0,61	0,44	0,07	0,05	0,34	0,30								
10	38,37	9,67	0,54	1,67	0,01	0,07	-0,01	0,08	0,01	0,14						
	39,47	49,24	0,00	0,01	0,00	0,01	0,00	0,01	0,08	0,15						
	2,85	2,35	0,62	0,52	0,04	0,03	0,07	0,16	0,49	0,37						
20	70,01	19,16	1,06	3,40	0,01	0,06	0,01	0,03	0,00	0,05	0,00	0,17				
	6,70	8,55	0,00	0,01	0,00	0,01	0,00	0,01	0,01	0,03	1,30	2,37				
	2,24	1,81	0,38	0,34	0,04	0,03	0,01	0,01	0,07	0,05	0,64	0,45				
50	112,60	45,83	-0,41	7,61	0,00	0,06	0,00	0,04	-0,50	1,88	0,01	0,08	-0,09	0,22		
	0,80	1,17	0,00	0,02	0,00	0,02	0,00	0,03	-0,01	0,09	0,22	0,47	1,91	1,79		
	1,91	1,43	0,44	0,57	0,04	0,07	0,02	0,07	0,44	1,52	0,09	0,07	0,43	0,31		
100	132,45	79,28	-0,62	6,14	-0,21	2,15	0,00	0,04	0,00	0,04	0,00	0,05	-0,09	0,15	-0,30	0,54
	0,69	1,02	0,00	0,05	0,01	0,06	0,01	0,06	0,05	0,12	0,65	1,00	1,29	1,09	0,21	0,60
	2,27	1,58	0,34	0,29	0,12	0,78	0,03	0,03	0,10	0,16	0,16	0,16	0,53	0,21	1,50	1,16

τ_2 × frame rate

k=0,01	0,2		0,5		1		2		5		10		20		50	
0,5	1,59	1,01														
	2407,53	2908,72														
	6,97	8,58														
1	3,70	0,92	0,65	0,36												
	799,09	807,04	682,71	965,23												
	5,88	5,24	4,59	3,72												
2	-26,74	314,48	1,67	1,02	0,33	0,35										
	266,70	366,79	239,15	399,81	321,30	389,40										
	5,99	5,06	2,80	1,74	2,77	1,45										
5	19,79	4,36	1,97	1,81	0,11	0,30	0,01	0,24								
	140,25	149,48	40,32	87,75	1,38	13,25	2,57	17,55								
	4,35	3,32	1,16	1,26	0,29	0,27	0,62	0,46								
10	41,68	9,09	2,49	3,33	0,03	0,15	0,01	0,13	-0,01	0,18						
	35,86	50,99	5,15	16,28	0,00	0,02	0,00	0,03	6,08	15,37						
	4,24	3,45	0,86	0,96	0,09	0,08	0,10	0,18	0,57	0,46						
20	69,81	22,02	2,02	3,96	-0,85	9,08	0,01	0,07	0,04	0,11	-0,10	0,25				
	10,35	11,71	0,55	2,05	0,00	0,02	0,00	0,02	0,05	0,09	5,76	6,50				
	2,69	2,32	0,70	0,61	0,10	0,28	0,02	0,02	0,16	0,12	0,56	0,42				
50	103,82	49,39	0,33	8,81	-0,04	0,14	0,01	0,05	-0,08	0,76	0,03	0,15	-0,14	0,28		
	1,42	1,78	0,07	0,50	-0,01	0,05	0,01	0,05	0,03	0,10	1,59	2,10	3,08	2,57		
	3,06	2,45	0,64	0,51	0,12	0,14	0,02	0,01	0,13	0,78	0,23	0,15	0,66	0,52		
100	108,90	66,01	-1,72	24,67	-0,43	3,02	0,00	0,04	0,00	0,05	-0,01	0,08	-0,19	0,21	-0,40	0,44
	1,46	1,44	0,05	0,10	0,02	0,10	0,04	0,13	0,36	0,65	1,87	1,83	1,95	1,49	0,87	1,57
	2,59	1,82	0,55	0,51	0,21	0,99	0,06	0,05	0,15	0,16	0,34	0,22	0,71	0,24	2,26	1,57

R=0.3

τ_2 × frame rate

k=0,02	0,2		0,5		1		2		5		10		20		50	
0,5	-20,72<br>1605,17<br>17,16	223,98<br>2300,00<br>24,26														
1	0,28<br>634,64<br>8,95	25,52<br>807,04<br>9,81	-0,50<br>841,14<br>7,01	9,00<br>904,26<br>7,32												
2	-72,34<br>328,95<br>7,85	791,96<br>388,90<br>8,31	2,00<br>305,45<br>5,52	0,78<br>557,99<br>5,93	-8,36<br>394,50<br>4,19	88,62<br>401,84<br>3,49										
5	21,02<br>106,83<br>6,83	4,61<br>124,30<br>6,08	4,61<br>85,14<br>2,70	2,01<br>113,57<br>3,94	0,64<br>29,26<br>1,16	0,80<br>64,27<br>0,90	0,26<br>47,91<br>1,70	0,41<br>79,56<br>1,71								
10	42,38<br>39,65<br>5,03	8,56<br>43,53<br>4,75	8,09<br>21,40<br>1,86	4,96<br>32,24<br>2,97	0,25<br>0,68<br>0,45	0,66<br>4,96<br>0,98	0,08<br>0,05<br>0,23	0,23<br>0,13<br>0,21	0,05<br>24,61<br>0,98	0,29<br>31,24<br>1,12						
20	73,19<br>10,74<br>4,23	20,85<br>10,98<br>4,16	10,55<br>3,58<br>1,82	8,29<br>6,25<br>3,01	0,23<br>0,01<br>0,21	0,85<br>0,04<br>0,22	0,06<br>0,03<br>0,06	0,15<br>0,05<br>0,04	0,05<br>1,07<br>0,25	0,21<br>3,07<br>0,22	0,01<br>12,30<br>0,64	0,25<br>10,07<br>0,81				
50	96,64<br>3,26<br>4,41	70,92<br>3,08<br>3,62	10,88<br>1,30<br>1,55	11,45<br>2,35<br>2,03	-0,28<br>0,01<br>0,27	2,72<br>0,08<br>0,48	0,03<br>0,06<br>0,05	0,11<br>0,12<br>0,07	-0,05<br>0,82<br>0,19	0,73<br>2,03<br>0,64	-0,02<br>4,31<br>0,50	0,19<br>3,65<br>0,34	-0,09<br>5,58<br>1,85	0,27<br>4,38<br>2,03		
100	93,00<br>1,42<br>4,85	69,39<br>1,63<br>3,78	11,76<br>0,88<br>1,72	15,40<br>1,49<br>2,08	-0,22<br>0,25<br>0,34	2,15<br>0,95<br>1,21	-0,15<br>0,37<br>0,29	1,57<br>0,87<br>1,56	0,00<br>2,27<br>0,29	0,08<br>2,64<br>0,24	-0,06<br>3,57<br>0,56	0,12<br>2,98<br>0,27	-0,21<br>2,71<br>1,17	0,25<br>2,34<br>1,15	-0,56<br>2,38<br>3,43	0,65<br>3,05<br>2,64

τ_2 × frame rate

k=0,05	0,2		0,5		1		2		5		10		20		50	
0,5	-93751,50<br>-23592,28<br>207,97	547422,09<br>148761,09<br>850,41														
1	-32559,86<br>-3748,82<br>71,07	322349,38<br>46481,57<br>520,95	-17946,39<br>-5465,96<br>415,44	133205,63<br>46223,41<br>2591,46												
2	9,06<br>308,18<br>15,34	2,89<br>348,55<br>17,45	2,62<br>291,62<br>13,29	1,03<br>309,87<br>15,11	-7,55<br>301,88<br>11,53	82,56<br>346,05<br>11,91										
5	22,62<br>81,94<br>16,17	6,46<br>88,33<br>14,92	6,35<br>102,89<br>8,06	2,34<br>128,37<br>10,36	1,85<br>84,72<br>4,52	1,02<br>105,62<br>6,12	0,61<br>111,84<br>4,87	0,37<br>115,72<br>6,60								
10	41,86<br>45,61<br>9,04	11,45<br>42,85<br>8,60	12,38<br>36,48<br>3,83	5,12<br>38,49<br>5,77	2,93<br>19,44<br>2,48	1,93<br>29,11<br>4,02	0,51<br>11,48<br>1,10	0,66<br>23,64<br>2,71	0,19<br>39,53<br>3,13	0,33<br>41,15<br>4,40						
20	69,84<br>13,09<br>8,24	21,44<br>11,28<br>12,08	22,19<br>12,70<br>5,73	8,40<br>12,81<br>9,71	3,05<br>4,47<br>0,59	2,98<br>7,91<br>1,09	0,39<br>3,15<br>0,27	0,60<br>6,66<br>0,40	0,18<br>10,82<br>0,33	0,37<br>12,57<br>0,25	0,13<br>16,28<br>0,91	0,25<br>14,41<br>1,82				
50	78,54<br>6,61<br>9,07	39,52<br>6,14<br>9,31	20,84<br>5,48<br>6,06	14,52<br>5,69<br>6,45	1,45<br>1,98<br>1,33	2,98<br>3,84<br>2,48	0,14<br>1,64<br>0,25	0,43<br>3,69<br>0,48	-0,01<br>5,68<br>0,36	0,22<br>6,55<br>0,23	-0,05<br>9,67<br>1,26	0,31<br>8,31<br>2,10	-0,35<br>7,74<br>4,74	2,02<br>6,55<br>5,30		
100	223,59<br>2,16<br>11,83	321,63<br>2,51<br>10,66	44,07<br>3,19<br>8,09	83,31<br>3,40<br>9,08	0,18<br>3,98<br>1,21	4,84<br>4,09<br>2,79	-0,26<br>4,25<br>0,64	2,06<br>4,82<br>1,97	-0,29<br>5,88<br>0,73	1,44<br>5,24<br>1,48	-0,16<br>6,15<br>0,75	0,22<br>4,98<br>0,32	-0,16<br>5,06<br>5,36	0,35<br>4,05<br>6,27	-0,59<br>9,73<br>6,67	0,56<br>11,82<br>6,26

R=0.4

τ_1 × frame rate

τ_2 × frame rate (rows)

k = 0,001	0,2	0,5	1	2	5	10	20	50
0,5	1,25 0,40<br>3513,75 4123,09<br>2,46 1,09							
1	3,69 0,48<br>2629,44 2818,84<br>2,33 0,62	0,19 0,26<br>595,05 1313,41<br>1,35 0,54						
2	6,81 1,92<br>783,64 994,37<br>1,52 0,94	-0,01 0,13<br>0,00 0,01<br>0,22 0,21	-0,01 0,09<br>0,01 0,04<br>0,20 0,18					
5	12,07 5,49<br>123,06 199,17<br>0,84 0,69	-0,01 0,05<br>0,00 0,00<br>0,08 0,07	0,00 0,02<br>0,00 0,00<br>0,01 0,01	0,00 0,02<br>0,00 0,01<br>0,03 0,03				
10	24,13 10,73<br>22,18 41,52<br>0,83 0,68	0,00 0,05<br>0,00 0,00<br>0,07 0,05	0,00 0,01<br>0,00 0,00<br>0,01 0,00	0,00 0,01<br>0,00 0,00<br>0,01 0,00	0,00 0,02<br>0,00 0,01<br>0,08 0,06			
20	25,04 21,72<br>0,81 4,47<br>1,14 0,67	0,00 0,03<br>0,00 0,00<br>0,05 0,04	0,00 0,01<br>0,00 0,00<br>0,01 0,00	0,00 0,01<br>0,00 0,00<br>0,00 0,00	0,00 0,01<br>0,00 0,01<br>0,01 0,01	0,00 0,04<br>0,01 0,04<br>0,16 0,11		
50	36,09 37,61<br>0,05 0,12<br>1,31 0,45	-0,06 0,14<br>-0,01 0,02<br>0,20 0,38	0,00 0,01<br>0,00 0,00<br>0,01 0,00	0,00 0,00<br>0,00 0,01<br>0,00 0,00	0,00 0,00<br>0,00 0,01<br>0,00 0,00	0,00 0,01<br>0,00 0,03<br>0,02 0,01	0,00 0,07<br>0,34 0,54<br>0,24 0,18	
100	58,75 69,03<br>0,03 0,14<br>1,27 0,45	-0,01 0,03<br>0,00 0,01<br>0,05 0,05	-0,01 0,05<br>0,00 0,03<br>0,02 0,05	0,00 0,00<br>0,00 0,01<br>0,01 0,01	0,00 0,00<br>0,01 0,02<br>0,01 0,01	0,00 0,01<br>0,02 0,07<br>0,02 0,02	0,09 0,12<br>0,18 0,46<br>1,38 1,48	-0,18 0,15<br>-0,11 0,12<br>0,72 0,47

k=0,002	0,2	0,5	1	2	5	10	20	50
0,5	1,34 0,48<br>2849,25 3413,91<br>3,13 1,80							
1	3,66 0,67<br>1744,13 1659,65<br>2,78 1,27	0,36 0,33<br>1120,57 1514,10<br>1,75 0,63						
2	7,83 1,49<br>662,35 724,25<br>2,42 1,32	0,04 0,24<br>0,01 0,02<br>0,44 0,32	-0,02 0,15<br>12,54 123,90<br>0,43 0,33					
5	17,42 5,02<br>134,38 170,53<br>1,64 1,09	0,00 0,11<br>0,00 0,00<br>0,19 0,15	0,00 0,03<br>0,00 0,01<br>0,01 0,01	0,00 0,04<br>0,00 0,02<br>0,08 0,06				
10	33,27 10,42<br>44,48 61,96<br>1,21 1,05	0,00 0,09<br>0,00 0,00<br>0,14 0,12	0,00 0,02<br>0,00 0,00<br>0,01 0,01	0,00 0,01<br>0,00 0,01<br>0,01 0,01	0,00 0,04<br>0,00 0,03<br>0,13 0,11			
20	21,89 208,21<br>2,58 7,35<br>1,39 1,10	0,00 0,06<br>0,00 0,00<br>0,11 0,10	0,00 0,01<br>0,00 0,00<br>0,01 0,01	0,00 0,01<br>0,00 0,00<br>0,00 0,00	0,00 0,01<br>0,00 0,01<br>0,02 0,02	0,00 0,06<br>0,05 0,11<br>0,27 0,18		
50	95,99 53,70<br>0,18 0,30<br>1,51 0,93	-0,12 0,18<br>-0,01 0,02<br>0,36 0,43	0,00 0,01<br>0,00 0,01<br>0,01 0,01	0,00 0,01<br>0,00 0,01<br>0,00 0,00	0,00 0,01<br>0,00 0,02<br>0,00 0,00	0,00 0,03<br>0,03 0,08<br>0,03 0,03	-0,06 0,14<br>0,81 1,39<br>0,39 0,29	
100	99,83 99,14<br>0,22 0,39<br>1,78 0,75	-0,01 0,08<br>0,00 0,02<br>0,11 0,10	0,00 0,04<br>0,00 0,03<br>0,02 0,03	0,00 0,01<br>0,01 0,03<br>0,02 0,01	0,00 0,01<br>0,03 0,06<br>0,03 0,02	0,00 0,01<br>0,11 0,20<br>0,05 0,05	0,02 0,12<br>0,54 0,73<br>0,85 1,18	-0,26 0,21<br>-0,13 0,14<br>0,70 0,48

k=0,005	0,2	0,5	1	2	5	10	20	50
0,5	1,55 0,68<br>2316,48 2266,35<br>4,84 4,65							
1	3,51 0,92<br>898,81 1076,65<br>3,68 3,03	0,52 0,28<br>746,84 1065,65<br>2,46 1,75						
2	8,33 1,56<br>524,01 608,29<br>3,81 2,88	0,63 0,64<br>74,33 196,69<br>1,00 0,62	0,07 0,27<br>160,92 343,15<br>1,06 0,68					
5	18,11 5,27<br>101,87 141,39<br>2,82 2,76	0,09 0,42<br>0,01 0,03<br>0,49 0,39	0,01 0,09<br>0,00 0,02<br>0,05 0,04	0,01 0,10<br>0,02 0,05<br>0,18 0,20				
10	37,83 9,63<br>40,33 55,55<br>2,34 2,10	0,25 1,21<br>0,00 0,01<br>0,41 0,40	0,01 0,05<br>0,00 0,01<br>0,03 0,02	0,00 0,04<br>0,00 0,01<br>0,03 0,02	0,00 0,09<br>0,05 0,10<br>0,32 0,25			
20	65,21 21,36<br>4,66 8,23<br>2,59 2,21	0,02 0,16<br>0,00 0,01<br>0,29 0,25	0,00 0,04<br>0,00 0,01<br>0,03 0,02	0,00 0,02<br>0,00 0,01<br>0,01 0,01	0,01 0,04<br>0,01 0,03<br>0,06 0,04	-0,03 0,14<br>1,31 2,85<br>0,46 0,31		
50	98,40 47,02<br>0,80 1,07<br>2,32 1,62	-0,10 0,19<br>-0,01 0,03<br>0,40 0,34	0,00 0,04<br>0,00 0,03<br>0,04 0,03	0,00 0,02<br>0,00 0,03<br>0,01 0,01	0,00 0,02<br>0,01 0,06<br>0,01 0,01	0,01 0,06<br>0,28 0,62<br>0,09 0,09	-0,13 0,21<br>2,00 1,96<br>0,53 0,38	
100	132,93 63,11<br>0,66 0,90<br>2,64 1,41	-0,60 5,72<br>0,01 0,06<br>0,31 0,47	0,00 0,05<br>0,01 0,06<br>0,05 0,05	-0,19 1,94<br>0,01 0,08<br>0,16 1,18	0,00 0,02<br>0,05 0,18<br>0,06 0,06	-0,01 0,04<br>0,65 0,95<br>0,18 0,17	-0,10 0,16<br>1,08 1,23<br>0,72 0,54	-0,38 0,56<br>0,00 0,37<br>1,74 1,32

k=0,01	0,2	0,5	1	2	5	10	20	50
0,5	1,99 1,05<br>1670,50 1954,99<br>6,85 8,03							
1	3,80 0,95<br>787,85 1023,60<br>5,19 5,79	0,59 0,33<br>830,84 989,01<br>3,99 3,33						
2	8,33 1,81<br>323,64 382,95<br>5,38 5,33	1,26 0,79<br>216,71 354,16<br>1,52 0,84	0,23 0,29<br>306,53 471,29<br>1,91 1,49					
5	20,33 5,05<br>119,44 123,36<br>4,32 4,11	1,12 1,22<br>22,52 59,85<br>0,70 0,47	0,07 0,20<br>0,03 0,08<br>0,14 0,16	-0,01 0,19<br>2,64 17,63<br>0,38 0,28				
10	39,41 21,63<br>36,29 45,11<br>3,65 3,52	0,92 1,95<br>2,76 10,85<br>0,52 0,35	0,01 0,11<br>0,00 0,02<br>0,07 0,06	0,01 0,08<br>0,01 0,03<br>0,06 0,05	-0,02 0,17<br>4,96 12,20<br>0,47 0,37			
20	73,26 18,49<br>10,75 13,09<br>3,42 2,97	0,89 2,93<br>0,16 1,11<br>0,51 0,41	-0,01 0,08<br>0,00 0,02<br>0,06 0,06	0,01 0,05<br>0,01 0,03<br>0,02 0,01	0,00 0,08<br>0,03 0,10<br>0,11 0,09	-0,05 0,21<br>7,66 7,65<br>0,48 0,37		
50	98,28 44,98<br>1,62 1,91<br>3,47 2,42	0,16 1,88<br>0,00 0,05<br>0,51 0,50	0,00 0,08<br>0,00 0,05<br>0,07 0,12	0,00 0,04<br>0,01 0,06<br>0,03 0,02	0,00 0,06<br>0,07 0,18<br>0,04 0,04	-0,01 0,10<br>1,22 1,83<br>0,24 0,19	-0,16 0,24<br>3,62 2,93<br>0,64 0,51	
100	105,13 60,88<br>1,15 1,34<br>3,31 1,86	-2,52 13,45<br>0,02 0,14<br>0,63 0,94	0,00 0,07<br>0,07 0,18<br>0,12 0,09	-0,17 2,03<br>0,08 0,24<br>0,21 1,27	-0,01 0,05<br>0,51 0,97<br>0,18 0,17	-0,02 0,06<br>1,76 1,70<br>0,39 0,26	-0,15 0,19<br>2,06 1,59<br>0,89 0,45	-0,36 0,36<br>0,44 0,96<br>2,40 1,70

R=0.4

τ_2 × frame rate

k=0,02	0,2		0,5		1		2		5		10		20		50	
0,5	3,34<br>2161,44<br>11,35	4,55<br>2725,14<br>16,54														
1	4,01<br>690,25<br>8,23	1,30<br>909,56<br>10,17	0,70<br>704,37<br>5,71	0,48<br>745,52<br>6,22												
2	-44,66<br>275,39<br>8,23	526,45<br>339,00<br>9,35	1,75<br>268,35<br>4,37	0,74<br>334,05<br>5,11	0,38<br>273,82<br>4,28	0,30<br>341,08<br>5,25										
5	19,97<br>100,11<br>6,18	5,98<br>119,46<br>6,36	3,57<br>63,75<br>1,81	2,36<br>99,66<br>2,57	0,42<br>16,98<br>0,66	0,53<br>53,66<br>0,52	0,11<br>40,42<br>0,83	0,30<br>78,99<br>0,67								
10	-8,33<br>39,20<br>4,77	498,12<br>41,37<br>4,84	5,16<br>20,67<br>0,83	4,07<br>36,55<br>1,04	0,08<br>0,02<br>0,18	0,28<br>0,04<br>0,19	0,04<br>0,07<br>0,14	0,17<br>0,35<br>0,13	-0,03<br>17,92<br>0,67	0,22<br>27,00<br>0,95						
20	66,96<br>8,47<br>5,28	19,61<br>9,85<br>4,51	6,81<br>3,26<br>0,99	6,53<br>6,14<br>1,37	0,03<br>0,01<br>0,13	0,17<br>0,04<br>0,12	0,01<br>0,01<br>0,04	0,10<br>0,05<br>0,03	0,01<br>0,29<br>0,20	0,15<br>1,24<br>0,15	-0,05<br>12,87<br>0,50	0,26<br>11,57<br>0,36				
50	94,99<br>2,74<br>5,01	51,78<br>3,03<br>3,84	7,22<br>0,86<br>1,43	9,30<br>1,79<br>1,75	0,03<br>0,04<br>0,14	0,15<br>0,09<br>0,15	0,02<br>0,06<br>0,05	0,07<br>0,13<br>0,04	0,00<br>1,04<br>0,13	0,10<br>2,27<br>0,15	-0,03<br>4,53<br>0,61	0,16<br>3,89<br>0,24	-0,17<br>5,71<br>1,61	0,25<br>5,17<br>1,67		
100	87,31<br>1,38<br>5,09	164,78<br>1,54<br>4,14	4,21<br>0,46<br>0,99	11,25<br>1,12<br>1,28	0,04<br>0,50<br>0,23	0,15<br>1,00<br>0,18	-0,20<br>0,80<br>0,30	1,97<br>1,58<br>0,98	-0,01<br>2,51<br>0,36	0,06<br>2,64<br>0,28	-0,09<br>2,60<br>0,61	0,12<br>2,18<br>0,32	-0,28<br>2,40<br>1,24	0,23<br>2,22<br>0,86	-0,42<br>2,07<br>3,61	0,21<br>2,78<br>2,31

τ_2 × frame rate

k=0,05	0,2		0,5		1		2		5		10		20		50	
0,5	-174985,6<br>-34786,4<br>392,8	734116,9<br>183713,3<br>1025,9														
1	-94,6<br>648,0<br>20,5	673,1<br>799,1<br>22,6	-3946,77<br>-756,15<br>35,73	39239,12<br>15229,27<br>215,71												
2	9,1<br>272,8<br>12,9	3,3<br>315,0<br>14,9	-12509,58<br>-2057,47<br>248,55	124493,98<br>23834,43<br>2367,50	0,62<br>287,34<br>10,61	0,44<br>319,16<br>12,68										
5	-202,9<br>116,9<br>11,7	2231,7<br>130,4<br>11,6	5,89<br>122,58<br>6,18	2,45<br>120,22<br>8,10	1,45<br>81,48<br>3,25	1,00<br>109,05<br>5,94	0,39<br>92,20<br>2,76	0,42<br>119,47<br>3,67								
10	44,0<br>40,3<br>8,0	13,2<br>40,5<br>8,8	11,26<br>31,80<br>4,49	5,04<br>40,34<br>7,12	1,45<br>13,00<br>0,82	1,61<br>25,43<br>1,68	0,41<br>14,63<br>0,62	0,54<br>26,89<br>1,21	0,11<br>33,80<br>3,46	0,29<br>37,95<br>5,17						
20	69,1<br>11,8<br>9,8	26,9<br>12,1<br>12,4	20,37<br>11,37<br>6,10	11,20<br>10,44<br>10,71	1,50<br>1,79<br>1,51	2,43<br>5,28<br>4,15	0,13<br>0,95<br>0,14	0,36<br>3,04<br>0,11	0,03<br>9,39<br>0,32	0,26<br>12,79<br>0,24	0,02<br>13,16<br>0,53	0,29<br>12,91<br>0,85				
50	86,0<br>4,0<br>10,8	116,8<br>4,3<br>9,6	16,74<br>4,32<br>6,88	13,41<br>5,26<br>7,15	0,56<br>1,92<br>0,86	3,71<br>3,89<br>2,05	0,00<br>1,25<br>0,19	0,20<br>3,51<br>0,19	-0,11<br>7,34<br>0,54	1,02<br>8,07<br>0,67	-0,14<br>9,28<br>1,02	0,81<br>6,28<br>0,93	-0,12<br>7,82<br>5,39	0,25<br>6,35<br>6,42		
100	151,7<br>2,1<br>12,0	238,0<br>2,7<br>11,5	41,24<br>3,71<br>5,50	76,86<br>4,66<br>7,16	0,35<br>4,45<br>0,87	1,52<br>4,43<br>2,04	-0,19<br>4,76<br>0,56	2,15<br>4,72<br>0,86	-0,06<br>5,97<br>0,58	0,12<br>4,51<br>0,37	-0,17<br>5,60<br>0,88	0,23<br>3,74<br>0,43	-0,39<br>3,51<br>4,28	1,29<br>3,49<br>3,81	-0,56<br>5,64<br>6,72	0,50<br>7,26<br>5,89

R=0.5

τ_1 × frame rate (columns); τ_2 × frame rate (rows)

k = 0,001	0,2		0,5		1		2		5		10		20		50	
0,5	1,45<br>3362,60<br>2,51	0,31<br>3180,67<br>1,15														
1	3,30<br>735,28<br>1,90	0,72<br>1270,12<br>1,17	-0,34<br>1184,95<br>1,18	3,53<br>1968,92<br>0,40												
2	7,23<br>1017,17<br>1,35	1,85<br>1146,87<br>0,66	0,01<br>0,00<br>0,13	0,12<br>0,01<br>0,15	-0,01<br>0,01<br>0,19	0,08<br>0,04<br>0,13										
5	9,32<br>83,07<br>0,80	6,21<br>178,09<br>0,54	0,00<br>0,00<br>0,07	0,05<br>0,00<br>0,05	-0,01<br>0,00<br>0,01	0,01<br>0,00<br>0,00	0,00<br>0,00<br>0,04	0,02<br>0,01<br>0,03								
10	15,98<br>10,96<br>1,00	11,11<br>24,82<br>0,58	-0,01<br>0,00<br>0,05	0,03<br>0,00<br>0,05	0,00<br>0,00<br>0,00	0,01<br>0,00<br>0,00	0,01<br>0,01<br>0,01	0,08<br>0,09<br>0,06	0,00<br>0,00<br>0,07	0,02<br>0,02<br>0,05						
20	15,36<br>0,89<br>1,18	18,46<br>4,26<br>0,64	0,00<br>0,00<br>0,04	0,02<br>0,00<br>0,03	0,00<br>0,00<br>0,00	0,01<br>0,00<br>0,00	0,00<br>0,00<br>0,00	0,00<br>0,00<br>0,00	0,00<br>0,00<br>0,01	0,01<br>0,01<br>0,01	-0,01<br>0,01<br>0,15	0,03<br>0,05<br>0,11				
50	36,02<br>0,07<br>1,46	39,34<br>0,17<br>0,56	-0,27<br>-0,05<br>0,63	0,26<br>0,05<br>0,56	0,00<br>0,00<br>0,01	0,01<br>0,01<br>0,00	0,00<br>0,00<br>0,00	0,00<br>0,01<br>0,00	0,00<br>0,00<br>0,00	0,00<br>0,01<br>0,00	0,00<br>0,01<br>0,01	0,01<br>0,04<br>0,01	-0,01<br>0,40<br>0,23	0,06<br>0,73<br>0,18		
100	50,77<br>0,08<br>1,72	69,80<br>0,24<br>0,46	0,00<br>0,00<br>0,05	0,04<br>0,02<br>0,06	-0,02<br>-0,01<br>0,03	0,08<br>0,06<br>0,08	0,00<br>0,00<br>0,01	0,00<br>0,02<br>0,01	0,00<br>0,01<br>0,02	0,00<br>0,03<br>0,01	0,00<br>0,01<br>0,03	0,01<br>0,08<br>0,02	-0,01<br>0,30<br>0,16	0,03<br>0,40<br>0,09	-0,33<br>-0,12<br>1,04	0,20<br>0,28<br>0,48

k=0,002	0,2		0,5		1		2		5		10		20		50	
0,5	1,40<br>2798,40<br>2,60	0,40<br>2989,50<br>1,46														
1	3,23<br>398,32<br>2,22	0,82<br>723,85<br>1,46	0,46<br>1583,98<br>1,71	0,28<br>1725,59<br>0,57												
2	7,48<br>788,71<br>1,67	2,86<br>792,29<br>1,03	0,02<br>10,05<br>0,39	0,25<br>99,88<br>0,31	-0,01<br>0,05<br>0,34	0,14<br>0,11<br>0,27										
5	14,80<br>106,83<br>1,06	6,01<br>149,88<br>0,81	0,01<br>0,00<br>0,13	0,08<br>0,01<br>0,09	0,00<br>0,00<br>0,01	0,03<br>0,01<br>0,01	0,00<br>0,00<br>0,06	0,04<br>0,02<br>0,06								
10	28,37<br>35,63<br>1,17	12,62<br>57,37<br>1,08	0,00<br>0,00<br>0,10	0,07<br>0,00<br>0,08	0,00<br>0,00<br>0,01	0,02<br>0,00<br>0,01	0,00<br>0,00<br>0,01	0,01<br>0,01<br>0,01	-0,01<br>0,00<br>0,15	0,04<br>0,03<br>0,10						
20	41,76<br>2,47<br>1,59	27,00<br>8,34<br>1,13	-0,01<br>0,00<br>0,09	0,06<br>0,00<br>0,07	0,00<br>0,00<br>0,01	0,01<br>0,00<br>0,01	0,00<br>0,00<br>0,00	0,01<br>0,01<br>0,00	0,00<br>0,00<br>0,02	0,01<br>0,02<br>0,02	-0,01<br>0,06<br>0,23	0,05<br>0,13<br>0,16				
50	80,15<br>0,23<br>1,97	53,41<br>0,52<br>0,86	-0,21<br>-0,04<br>0,54	0,22<br>0,04<br>0,48	0,00<br>0,00<br>0,01	0,01<br>0,01<br>0,01	0,00<br>0,00<br>0,01	0,01<br>0,01<br>0,00	0,00<br>0,00<br>0,00	0,01<br>0,03<br>0,00	0,00<br>0,03<br>0,03	0,02<br>0,08<br>0,02	-0,04<br>1,21<br>0,39	0,11<br>1,63<br>0,31		
100	102,29<br>0,28<br>2,11	68,27<br>0,45<br>0,65	-1,30<br>0,00<br>0,29	7,40<br>0,06<br>1,17	-0,02<br>-0,01<br>0,03	0,07<br>0,06<br>0,06	0,00<br>0,01<br>0,02	0,01<br>0,04<br>0,02	0,00<br>0,02<br>0,03	0,01<br>0,07<br>0,03	0,00<br>0,14<br>0,08	0,01<br>0,33<br>0,09	-0,04<br>0,70<br>0,40	0,07<br>0,81<br>0,18	-0,35<br>-0,10<br>1,14	0,22<br>0,27<br>0,59

k=0,005	0,2		0,5		1		2		5		10		20		50	
0,5	1,56<br>1831,34<br>4,45	0,77<br>2184,79<br>5,26														
1	3,53<br>662,53<br>3,23	0,87<br>843,53<br>3,26	0,53<br>1472,40<br>2,16	0,24<br>1544,04<br>1,37												
2	8,10<br>491,25<br>3,03	1,70<br>617,43<br>2,66	0,46<br>124,46<br>0,72	0,55<br>298,16<br>0,44	0,02<br>94,86<br>0,75	0,27<br>252,30<br>0,44										
5	17,07<br>75,18<br>2,66	5,85<br>115,18<br>2,40	0,00<br>0,00<br>0,39	0,21<br>0,01<br>0,30	0,00<br>0,00<br>0,04	0,07<br>0,02<br>0,03	-0,02<br>0,01<br>0,18	0,09<br>0,06<br>0,16								
10	36,75<br>34,93<br>2,25	9,90<br>44,80<br>2,16	0,05<br>0,00<br>0,29	0,59<br>0,01<br>0,22	0,00<br>0,00<br>0,02	0,04<br>0,01<br>0,02	0,00<br>0,00<br>0,03	0,03<br>0,02<br>0,02	-0,03<br>0,07<br>0,30	0,08<br>0,28<br>0,24						
20	54,38<br>7,65<br>2,52	76,05<br>10,83<br>2,27	0,00<br>0,00<br>0,28	0,15<br>0,01<br>0,20	0,01<br>0,00<br>0,02	0,03<br>0,01<br>0,02	0,00<br>0,00<br>0,01	0,02<br>0,02<br>0,01	0,00<br>0,01<br>0,05	0,03<br>0,04<br>0,04	-0,01<br>1,57<br>0,38	0,11<br>3,05<br>0,29				
50	113,64<br>1,45<br>2,58	49,58<br>2,28<br>1,80	-0,17<br>-0,02<br>0,48	0,23<br>0,04<br>0,39	0,00<br>0,00<br>0,03	0,03<br>0,02<br>0,02	0,00<br>0,00<br>0,01	0,04<br>0,03<br>0,03	0,00<br>0,00<br>0,01	0,02<br>0,06<br>0,01	0,00<br>0,28<br>0,10	0,05<br>0,55<br>0,09	-0,13<br>2,46<br>0,65	0,17<br>2,47<br>0,41		
100	116,77<br>0,74<br>3,10	56,36<br>0,76<br>1,50	-1,81<br>0,00<br>0,50	8,72<br>0,09<br>1,22	0,00<br>0,03<br>0,06	0,05<br>0,09<br>0,05	-0,01<br>0,01<br>0,05	0,05<br>0,10<br>0,04	0,00<br>0,22<br>0,12	0,02<br>0,53<br>0,13	-0,01<br>0,91<br>0,27	0,03<br>1,02<br>0,23	-0,12<br>1,06<br>0,74	0,12<br>1,17<br>0,28	-0,36<br>-0,04<br>2,00	0,22<br>0,38<br>1,13

k=0,01	0,2		0,5		1		2		5		10		20		50	
0,5	1,88<br>1825,15<br>6,73	1,34<br>2354,04<br>9,20														
1	3,61<br>548,38<br>4,41	1,15<br>837,44<br>5,37	0,53<br>975,23<br>2,98	0,32<br>1166,66<br>3,00												
2	7,91<br>394,72<br>4,06	1,66<br>442,30<br>4,23	0,93<br>129,21<br>1,05	0,78<br>249,48<br>0,66	0,18<br>251,67<br>1,35	0,28<br>387,90<br>0,77										
5	20,99<br>142,53<br>4,78	4,52<br>172,15<br>4,84	0,38<br>4,33<br>0,53	0,81<br>22,61<br>0,35	0,01<br>0,02<br>0,11	0,14<br>0,04<br>0,10	-2,63<br>0,11<br>0,35	25,93<br>0,29<br>0,33								
10	38,75<br>37,38<br>3,23	11,20<br>42,50<br>3,49	0,35<br>1,49<br>0,44	1,21<br>7,82<br>0,36	0,00<br>0,00<br>0,06	0,07<br>0,02<br>0,04	0,00<br>0,01<br>0,05	0,06<br>0,03<br>0,04	-0,03<br>3,03<br>0,44	0,14<br>9,74<br>0,33						
20	68,19<br>9,39<br>4,46	19,87<br>12,26<br>3,70	-3,67<br>0,12<br>0,49	44,26<br>1,22<br>0,37	-0,02<br>-0,01<br>0,09	0,10<br>0,03<br>0,17	0,00<br>0,00<br>0,02	0,04<br>0,03<br>0,01	0,01<br>0,04<br>0,12	0,07<br>0,10<br>0,08	-0,09<br>6,96<br>0,53	0,18<br>6,82<br>0,39				
50	86,80<br>2,03<br>3,78	53,49<br>2,38<br>2,99	0,01<br>0,00<br>0,41	0,34<br>0,05<br>0,33	0,00<br>0,01<br>0,06	0,06<br>0,06<br>0,04	-0,01<br>0,01<br>0,03	0,03<br>0,07<br>0,02	0,00<br>0,11<br>0,05	0,04<br>0,25<br>0,05	-0,01<br>2,27<br>0,38	0,08<br>2,48<br>0,25	-0,24<br>3,73<br>1,08	0,24<br>3,18<br>0,61		
100	80,76<br>1,13<br>3,95	66,86<br>1,31<br>3,02	-1,25<br>0,08<br>0,56	7,49<br>0,24<br>1,16	0,01<br>0,15<br>0,13	0,06<br>0,38<br>0,11	0,00<br>0,16<br>0,11	0,05<br>0,27<br>0,10	-0,01<br>0,71<br>0,24	0,05<br>1,20<br>0,22	-0,03<br>1,97<br>0,49	0,06<br>1,67<br>0,29	-0,23<br>1,70<br>1,05	0,19<br>1,39<br>0,36	-0,49<br>0,37<br>2,86	0,69<br>0,98<br>1,40

R=0.5

Rows: τ_2 × frame rate

k=0,02	0,2	0,5	1	2	5	10	20	50
0,5	15,20 98,44<br>1786,12 2510,69<br>17,92 23,24							
1	1,88 19,34<br>606,89 749,26<br>8,15 11,66	0,61 0,51<br>604,03 765,18<br>4,61 6,05						
2	8,22 2,31<br>296,57 393,44<br>7,58 9,74	1,49 0,83<br>213,51 286,90<br>3,21 5,09	0,26 0,35<br>297,56 378,29<br>3,23 3,43					
5	20,38 5,17<br>100,63 133,94<br>6,74 6,81	2,51 1,93<br>47,94 87,31<br>0,96 1,36	0,18 0,33<br>6,23 28,15<br>0,44 0,48	-6,14 42,89<br>21,45 48,59<br>0,54 0,43				
10	41,40 12,53<br>29,32 37,29<br>4,69 5,41	3,05 3,32<br>9,56 21,11<br>0,73 1,01	0,05 0,31<br>0,33 3,01<br>0,15 0,14	0,01 0,14<br>0,03 0,09<br>0,11 0,09	-0,06 0,25<br>28,17 37,14<br>0,59 0,50			
20	23,03 444,45<br>9,86 11,57<br>7,37 8,05	4,35 5,76<br>1,38 4,38<br>1,11 1,67	-0,05 0,19<br>0,00 0,06<br>0,24 0,29	0,02 0,09<br>0,03 0,07<br>0,04 0,03	0,02 0,13<br>0,66 2,64<br>0,18 0,15	-0,14 0,23<br>11,02 11,82<br>0,61 0,40		
50	72,84 77,90<br>3,22 3,04<br>5,11 4,19	2,36 6,10<br>0,36 1,24<br>1,15 2,62	0,01 0,12<br>0,03 0,14<br>0,11 0,09	0,02 0,07<br>0,10 0,18<br>0,07 0,05	-0,01 0,06<br>0,91 2,01<br>0,16 0,18	-0,06 0,13<br>5,17 4,12<br>0,76 0,29	-0,43 2,01<br>5,57 4,22<br>2,18 1,80	
100	69,64 127,00<br>1,13 1,38<br>5,96 4,13	0,19 5,66<br>0,81 1,38<br>0,68 0,73	0,02 0,12<br>0,81 1,44<br>0,26 0,22	-0,01 0,06<br>1,26 2,03<br>0,31 0,28	-0,03 0,05<br>1,99 2,56<br>0,38 0,30	-0,09 0,11<br>3,34 2,11<br>0,81 0,33	-0,30 0,41<br>2,40 3,41<br>1,67 1,46	-0,49 0,48<br>2,03 2,80<br>3,78 2,56

Rows: τ_2 × frame rate

k=0,05	0,2	0,5	1	2	5	10	20	50
0,5	-96338,92 573261,14<br>-20478,57 123968,51<br>164,16 648,46							
1	-47720,09 288714,02<br>-5599,80 40565,36<br>102,04 460,97	-11752,40 109403,29<br>-3274,34 40339,56<br>166,20 1457,36						
2	-18524,46 182970,65<br>-1156,24 14592,11<br>167,17 1505,66	-11943,88 118861,51<br>-1920,06 22718,50<br>189,68 1806,33	0,52 0,41<br>392,83 336,75<br>8,97 10,66					
5	-31,49 537,70<br>88,07 107,47<br>13,24 13,87	5,41 2,74<br>77,15 94,61<br>8,13 10,50	0,99 0,83<br>59,34 93,13<br>2,15 4,11	0,27 0,33<br>87,09 111,01<br>2,94 4,97				
10	43,79 13,46<br>31,27 38,26<br>8,23 9,32	9,45 5,13<br>25,55 33,82<br>3,84 5,66	0,88 1,14<br>10,62 22,65<br>0,44 0,51	0,17 0,39<br>12,44 22,51<br>0,39 1,55	-0,06 0,28<br>42,60 49,90<br>1,31 2,23			
20	67,37 30,44<br>12,45 13,20<br>11,98 12,32	14,84 8,98<br>10,25 12,31<br>7,15 10,56	0,78 1,29<br>2,25 6,32<br>0,67 1,92	0,08 0,30<br>1,46 4,90<br>0,15 0,16	-0,02 0,24<br>11,07 14,53<br>0,40 0,27	0,00 0,24<br>17,97 17,88<br>0,52 0,37		
50	64,39 38,75<br>4,18 4,16<br>10,20 10,04	14,07 12,50<br>4,09 5,35<br>7,59 9,65	0,23 0,91<br>1,55 3,15<br>0,53 1,09	0,02 0,19<br>2,71 4,21<br>0,30 0,26	-0,02 0,15<br>7,02 7,22<br>0,60 0,41	-0,17 0,24<br>8,46 6,09<br>1,05 0,41	-0,20 0,26<br>7,80 6,34<br>6,10 5,61	
100	97,19 124,66<br>2,74 4,65<br>13,24 12,78	17,44 55,38<br>4,52 5,03<br>4,67 7,86	0,02 0,30<br>4,64 4,60<br>0,56 0,43	-0,03 0,13<br>4,16 4,08<br>0,50 0,36	-0,19 1,23<br>5,83 4,67<br>0,74 0,57	-0,18 0,21<br>4,41 3,74<br>1,10 0,66	-0,29 0,26<br>3,42 3,94<br>4,94 5,41	-0,56 0,29<br>6,54 7,88<br>5,67 4,50

R=0.6

τ_1 × frame rate (columns); τ_2 × frame rate (rows)

k = 0,001	0,2	0,5	1	2	5	10	20	50
0,5	1,40 0,28<br>3018,71 3157,93<br>1,98 0,81							
1	2,34 1,03<br>42,24 280,61<br>1,18 0,93	0,08 0,27<br>875,04 1802,26<br>0,85 0,45						
2	6,36 1,97<br>610,75 707,64<br>0,94 0,68	0,01 0,10<br>0,00 0,02<br>0,25 0,20	-0,01 0,06<br>0,01 0,05<br>0,18 0,14					
5	5,92 5,07<br>27,00 82,72<br>1,12 0,61	0,01 0,04<br>0,00 0,00<br>0,05 0,04	0,00 0,01<br>0,00 0,01<br>0,01 0,01	-61,08 114,36<br>-0,03 0,06<br>0,58 0,92				
10	12,91 11,79<br>11,16 25,59<br>1,50 1,09	0,01 0,03<br>0,00 0,00<br>0,04 0,03	0,00 0,01<br>0,00 0,00<br>0,00 0,00	0,00 0,01<br>0,00 0,00<br>0,01 0,00	0,00 0,02<br>0,00 0,02<br>0,08 0,06			
20	10,08 15,30<br>0,55 3,89<br>1,51 0,62	0,00 0,02<br>0,00 0,00<br>0,03 0,02	0,00 0,01<br>0,00 0,00<br>0,00 0,00	0,00 0,00<br>0,00 0,00<br>0,00 0,00	0,00 0,01<br>0,00 0,01<br>0,01 0,01	0,00 0,03<br>0,02 0,07<br>0,17 0,13		
50	25,65 34,89<br>0,11 0,42<br>1,70 0,50	0,00 0,03<br>0,00 0,01<br>0,04 0,03	0,00 0,01<br>0,00 0,01<br>0,00 0,00	0,00 0,00<br>0,00 0,01<br>0,00 0,00	0,00 0,00<br>0,00 0,02<br>0,00 0,00	0,00 0,01<br>0,01 0,05<br>0,01 0,01	-0,03 0,06<br>0,31 0,64<br>0,35 0,25	
100	23,08 37,35<br>0,08 0,23<br>1,94 0,35	0,00 0,02<br>0,00 0,02<br>0,04 0,03	0,00 0,03<br>0,00 0,03<br>0,01 0,02	0,00 0,00<br>0,00 0,03<br>0,01 0,01	0,00 0,00<br>0,01 0,04<br>0,02 0,02	0,00 0,01<br>0,07 0,18<br>0,06 0,06	-0,02 0,03<br>0,38 0,56<br>0,24 0,15	-0,40 0,22<br>-0,24 0,10<br>1,55 0,63

k=0,002	0,2	0,5	1	2	5	10	20	50
0,5	1,52 0,43<br>1808,62 2098,36<br>3,04 2,17							
1	2,61 1,11<br>136,47 447,99<br>1,46 1,99	0,19 0,30<br>1618,55 2113,29<br>1,12 0,56						
2	7,40 1,73<br>504,72 585,83<br>1,70 1,54	-0,02 0,22<br>0,01 0,04<br>0,32 0,24	-0,04 0,12<br>0,04 0,10<br>0,29 0,23					
5	13,49 5,79<br>82,34 128,53<br>1,28 1,36	0,00 0,08<br>0,00 0,01<br>0,12 0,09	0,00 0,02<br>0,00 0,01<br>0,01 0,01	-47,26 103,31<br>-0,03 0,07<br>0,55 0,88				
10	24,41 12,66<br>21,54 36,51<br>1,51 1,29	0,00 0,06<br>0,00 0,00<br>0,08 0,07	-0,01 0,05<br>0,00 0,01<br>0,02 0,06	0,00 0,01<br>0,00 0,01<br>0,01 0,01	0,00 0,03<br>0,01 0,04<br>0,15 0,11			
20	2,78 357,01<br>2,48 5,27<br>1,91 1,30	0,00 0,05<br>0,00 0,01<br>0,08 0,07	0,00 0,01<br>0,00 0,01<br>0,01 0,01	0,00 0,01<br>0,00 0,01<br>0,00 0,00	0,00 0,01<br>0,00 0,02<br>0,02 0,02	-0,01 0,05<br>0,07 0,16<br>0,29 0,21		
50	71,19 47,97<br>0,80 1,25<br>2,20 0,96	-0,01 0,06<br>0,00 0,02<br>0,07 0,07	0,00 0,01<br>0,00 0,01<br>0,01 0,01	0,00 0,01<br>0,00 0,02<br>0,01 0,01	0,00 0,01<br>0,01 0,04<br>0,01 0,00	0,00 0,02<br>0,03 0,10<br>0,03 0,02	-0,07 0,10<br>1,48 2,00<br>0,63 0,39	
100	88,71 66,46<br>0,38 0,51<br>2,58 0,84	0,00 0,04<br>0,00 0,04<br>0,08 0,06	0,00 0,03<br>0,00 0,05<br>0,03 0,02	0,00 0,01<br>0,01 0,04<br>0,02 0,02	0,00 0,01<br>0,02 0,10<br>0,05 0,03	0,00 0,01<br>0,36 0,53<br>0,16 0,15	-0,03 0,06<br>1,02 1,04<br>0,50 0,22	-0,35 0,19<br>-0,20 0,14<br>1,62 0,64

k=0,005	0,2	0,5	1	2	5	10	20	50
0,5	1,51 0,84<br>1527,27 1918,89<br>4,44 5,82							
1	2,80 1,24<br>135,88 376,58<br>2,51 4,14	0,36 0,33<br>1013,68 1154,60<br>1,59 1,17						
2	7,92 1,53<br>425,58 432,92<br>2,90 3,07	0,28 0,48<br>119,20 291,27<br>0,70 0,42	-0,02 0,26<br>98,97 328,69<br>0,62 0,48					
5	18,49 4,94<br>93,12 123,38<br>2,41 2,95	0,00 0,22<br>0,00 0,02<br>0,35 0,25	0,00 0,06<br>0,00 0,03<br>0,04 0,03	-30,93 85,00<br>-0,01 0,09<br>0,56 0,68				
10	34,44 10,70<br>39,62 53,39<br>2,37 2,36	0,01 0,13<br>0,00 0,01<br>0,19 0,15	-0,01 0,05<br>0,00 0,02<br>0,04 0,07	0,00 0,03<br>0,00 0,02<br>0,03 0,02	-0,02 0,08<br>0,08 0,17<br>0,33 0,25			
20	-20,36 798,84<br>9,23 10,71<br>2,94 2,70	-0,02 0,11<br>0,00 0,01<br>0,20 0,15	0,00 0,03<br>0,00 0,01<br>0,02 0,03	0,00 0,02<br>0,00 0,02<br>0,01 0,01	0,00 0,03<br>0,02 0,06<br>0,06 0,05	-0,04 0,10<br>2,25 3,45<br>0,50 0,34		
50	99,56 45,26<br>1,71 2,18<br>3,29 2,02	-0,02 0,13<br>0,00 0,03<br>0,22 0,17	0,00 0,04<br>0,00 0,04<br>0,03 0,04	0,00 0,01<br>0,01 0,04<br>0,01 0,01	0,00 0,02<br>0,02 0,10<br>0,02 0,01	-0,01 0,04<br>0,66 1,17<br>0,19 0,18	-0,16 0,18<br>4,01 3,04<br>1,11 0,52	
100	100,60 77,89<br>0,71 0,89<br>3,62 1,82	-0,03 0,11<br>0,01 0,09<br>0,22 0,17	-0,01 0,05<br>0,02 0,11<br>0,06 0,05	0,00 0,01<br>0,04 0,15<br>0,07 0,07	0,00 0,04<br>0,39 0,59<br>0,18 0,18	-0,01 0,03<br>1,23 1,27<br>0,40 0,30	-0,14 0,14<br>1,30 1,45<br>1,00 0,41	-0,32 0,22<br>-0,06 0,35<br>2,18 1,13

k=0,01	0,2	0,5	1	2	5	10	20	50
0,5	1,61 6,08<br>1873,06 2384,23<br>14,02 17,53							
1	3,34 1,26<br>313,42 585,64<br>5,25 7,36	0,49 0,30<br>844,09 980,18<br>2,81 3,37						
2	8,00 2,28<br>319,61 421,59<br>6,68 6,45	0,94 0,63<br>213,90 309,20<br>0,98 0,63	0,07 0,28<br>243,56 329,81<br>1,42 1,70					
5	19,42 4,84<br>100,94 150,20<br>4,35 5,35	0,25 0,64<br>3,20 15,69<br>0,39 0,27	0,01 0,14<br>0,02 0,07<br>0,10 0,09	-17,29 79,66<br>0,13 0,22<br>0,47 0,47				
10	39,75 10,14<br>38,92 43,56<br>3,89 3,95	0,08 0,40<br>0,01 0,03<br>0,36 0,30	0,01 0,07<br>0,01 0,03<br>0,05 0,04	0,00 0,07<br>0,01 0,05<br>0,06 0,05	-0,03 0,13<br>4,89 11,98<br>0,48 0,39			
20	63,28 37,39<br>9,44 10,57<br>4,30 3,94	0,21 1,38<br>0,13 1,27<br>0,39 0,36	-0,01 0,07<br>0,00 0,03<br>0,05 0,09	0,00 0,04<br>0,01 0,04<br>0,02 0,02	0,01 0,05<br>0,07 0,13<br>0,11 0,08	-0,10 0,16<br>8,09 7,49<br>0,66 0,47		
50	101,52 109,72<br>2,54 2,47<br>5,05 3,87	-0,03 0,21<br>0,00 0,06<br>0,34 0,25	0,01 0,05<br>0,02 0,08<br>0,05 0,04	0,00 0,03<br>0,01 0,09<br>0,03 0,02	0,00 0,03<br>0,19 0,47<br>0,07 0,08	-0,02 0,07<br>2,31 2,44<br>0,49 0,26	-0,28 0,23<br>3,46 3,48<br>1,44 0,84	
100	70,23 77,02<br>1,04 1,24<br>4,14 2,47	-1,83 13,75<br>0,26 0,59<br>0,51 1,33	0,00 0,05<br>0,17 0,32<br>0,14 0,11	0,00 0,05<br>0,38 0,79<br>0,19 0,18	0,00 0,03<br>1,95 2,17<br>0,46 0,40	-0,03 0,05<br>1,94 1,89<br>0,57 0,40	-0,21 0,18<br>1,96 1,66<br>1,32 0,46	-0,34 0,21<br>0,50 1,15<br>3,00 1,67

R=0.6

τ_2 × frame rate

k=0,02	0,2		0,5		1		2		5		10		20		50	
0,5	6,91	52,79														
	2347,58	2967,99														
	19,17	22,67														
1	4,05	1,64	-0,31	8,81												
	710,36	860,39	701,11	812,59												
	9,64	13,87	4,01	5,57												
2	8,38	2,32	1,41	0,68	-13,39	134,58										
	260,32	360,05	218,07	296,48	345,84	465,54										
	8,02	9,25	4,04	6,28	1,96	2,69										
5	-181,89	1577,53	1,96	1,72	0,05	0,30	-6,15	35,46								
	101,68	118,06	51,98	86,93	5,18	22,61	19,08	52,13								
	6,63	7,32	0,93	1,93	0,32	0,23	0,59	0,51								
10	42,66	12,25	2,62	3,05	0,01	0,16	0,01	0,12	-0,04	0,19						
	34,63	39,50	11,13	23,85	0,01	0,07	0,05	0,13	26,06	33,48						
	6,00	5,48	0,60	0,90	0,12	0,09	0,12	0,10	0,75	1,04						
20	64,05	27,24	2,65	4,12	-3,16	13,66	0,02	0,08	0,01	0,10	-0,16	0,22				
	9,80	11,54	0,99	3,00	0,00	0,08	0,04	0,11	1,37	3,61	12,36	13,57				
	6,83	6,67	0,85	1,31	0,66	2,17	0,04	0,03	0,22	0,16	0,90	0,53				
50	75,96	51,63	0,40	6,80	0,02	0,12	0,01	0,07	-0,01	0,07	-0,08	0,15	-0,20	0,22		
	2,71	3,39	0,21	0,87	0,11	0,22	0,29	1,02	1,92	3,38	5,29	4,59	5,18	4,78		
	7,61	6,43	0,82	2,10	0,12	0,10	0,09	0,12	0,29	0,33	0,99	0,41	2,86	2,22		
100	74,47	172,60	-0,84	12,16	0,01	0,12	-0,01	0,05	-0,01	0,05	-0,10	0,10	-0,29	0,21	-0,41	0,20
	1,68	2,08	1,32	1,83	1,24	1,97	1,78	2,35	3,29	2,96	2,60	1,96	2,09	2,15	1,45	2,24
	6,36	5,89	0,73	1,18	0,35	0,31	0,41	0,34	0,65	0,44	0,90	0,37	1,88	1,35	4,46	2,49

τ_2 × frame rate

k=0,05	0,2		0,5		1		2		5		10		20		50	
0,5	-140176,3	664331,6														
	-34148,1	182863,0														
	268,4	916,9														
1	-273616,5	835940,1	-32990,61	188870,73												
	-36001,2	112502,2	-10301,99	62096,01												
	423,5	1160,0	237,53	1396,36												
2	-59,2	669,2	-7745,10	76716,94	-3356,07	33396,50										
	325,7	381,1	-1143,89	14309,21	-871,52	12353,71										
	18,5	20,4	182,93	1725,09	124,11	1144,90										
5	23,4	8,5	4,40	2,36	-1,88	24,48	0,12	0,33								
	92,6	101,9	100,21	128,14	49,34	82,74	87,10	114,94								
	15,2	15,5	4,10	6,73	1,89	4,30	1,91	4,17								
10	40,3	24,7	4,79	23,45	0,44	0,70	-0,01	0,26	0,00	0,25						
	40,7	38,7	24,57	33,60	7,23	20,46	4,43	14,06	46,72	44,31						
	11,2	13,8	4,80	6,47	0,39	0,29	0,29	0,46	2,54	4,10						
20	67,9	33,9	12,75	10,48	0,41	1,10	0,04	0,20	-0,04	0,17	-0,09	0,24				
	11,6	13,0	13,09	14,89	1,19	4,54	1,12	4,32	13,18	14,18	17,71	16,42				
	17,6	16,1	6,65	10,79	0,37	0,43	0,13	0,12	0,63	0,42	0,81	0,48				
50	60,0	63,7	8,43	9,28	0,07	0,29	0,00	0,15	-0,05	0,15	-0,19	0,21	-0,23	0,23		
	4,4	4,7	4,96	5,63	2,43	4,47	4,51	5,80	8,17	6,89	8,49	6,98	6,36	6,22		
	18,8	18,3	6,16	9,59	0,43	0,33	0,46	0,41	0,79	0,48	1,44	0,87	6,08	5,04		
100	125,2	179,8	8,08	26,78	0,01	0,22	-0,02	0,11	-0,08	0,10	-0,17	0,18	-0,27	0,22	-0,55	0,23
	3,3	4,1	5,78	6,42	5,84	5,12	5,44	4,60	6,17	4,84	4,91	3,98	3,16	3,09	4,52	6,48
	19,9	22,8	5,01	10,89	0,73	0,49	0,74	0,50	0,88	0,54	1,21	0,60	5,67	4,84	6,46	5,10

R=0.7

τ_1 × frame rate (columns); τ_2 × frame rate (rows)

k = 0,001	0,2	0,5	1	2	5	10	20	50
0,5	1,35 0,35<br>2290,53 2665,98<br>2,20 1,69							
1	3,37 0,65<br>1225,55 1559,70<br>1,25 0,82	-0,76 0,27<br>130,72 510,52<br>3,26 1,09						
2	5,40 2,17<br>325,02 591,29<br>0,92 0,77	-0,02 0,11<br>0,00 0,03<br>0,14 0,23	-0,01 0,05<br>0,02 0,06<br>0,20 0,16					
5	5,61 4,42<br>41,09 88,23<br>1,43 0,72	0,00 0,03<br>0,00 0,01<br>0,04 0,03	0,00 0,01<br>0,00 0,01<br>0,01 0,01	0,00 0,01<br>0,00 0,02<br>0,07 0,12				
10	11,13 11,21<br>3,46 12,91<br>2,03 0,83	0,00 0,02<br>0,00 0,00<br>0,04 0,03	0,00 0,01<br>0,00 0,00<br>0,01 0,06	0,00 0,00<br>0,00 0,01<br>0,01 0,00	0,00 0,01<br>0,01 0,03<br>0,08 0,07			
20	9,71 14,95<br>0,13 0,89<br>1,98 0,96	0,00 0,02<br>0,00 0,00<br>0,04 0,03	-0,01 0,07<br>-0,01 0,03<br>0,15 0,33	0,00 0,00<br>0,00 0,01<br>0,00 0,00	0,00 0,00<br>0,00 0,01<br>0,01 0,01	0,00 0,02<br>0,03 0,08<br>0,18 0,12		
50	11,76 33,17<br>0,10 0,42<br>1,92 0,65	0,00 0,02<br>0,00 0,01<br>0,03 0,02	0,00 0,01<br>0,00 0,01<br>0,01 0,00	0,00 0,00<br>0,00 0,01<br>0,00 0,00	0,00 0,00<br>0,00 0,02<br>0,00 0,00	0,00 0,01<br>0,01 0,08<br>0,02 0,01	-0,03 0,06<br>0,32 0,57<br>0,46 0,40	
100	-2,08 75,69<br>0,04 0,18<br>2,29 0,66	0,00 0,02<br>0,00 0,02<br>0,04 0,02	0,00 0,00<br>0,00 0,02<br>0,02 0,01	0,00 0,00<br>0,00 0,03<br>0,06 0,16	0,00 0,00<br>0,02 0,07<br>0,03 0,03	0,00 0,01<br>0,14 0,29<br>0,09 0,09	-0,02 0,03<br>0,68 0,93<br>0,41 0,27	-0,41 0,21<br>-0,29 0,12<br>2,22 0,81

k=0,002	0,2	0,5	1	2	5	10	20	50
0,5	1,44 0,56<br>2124,99 2352,05<br>2,93 3,14							
1	3,47 0,70<br>1112,38 1247,10<br>1,76 1,85	-0,40 0,39<br>864,85 1439,89<br>2,19 1,54						
2	6,57 1,87<br>237,00 394,13<br>1,57 2,02	-0,02 0,18<br>0,01 0,05<br>0,24 0,22	-0,04 0,10<br>0,07 0,15<br>0,34 0,25					
5	11,75 6,06<br>105,86 158,27<br>1,25 1,13	0,01 0,08<br>0,00 0,01<br>0,11 0,10	0,00 0,02<br>0,00 0,01<br>0,02 0,01	0,00 0,02<br>0,00 0,03<br>0,13 0,17				
10	21,88 12,89<br>24,08 39,48<br>2,09 1,74	0,00 0,06<br>0,00 0,01<br>0,09 0,07	0,00 0,01<br>0,00 0,01<br>0,02 0,09	0,00 0,01<br>0,00 0,01<br>0,02 0,01	0,00 0,02<br>0,02 0,06<br>0,17 0,17			
20	-25,35 471,53<br>2,36 5,37<br>2,74 1,52	0,00 0,05<br>0,00 0,01<br>0,07 0,05	-0,02 0,07<br>-0,01 0,03<br>0,18 0,30	0,00 0,01<br>0,00 0,01<br>0,00 0,00	0,00 0,01<br>0,00 0,03<br>0,02 0,02	-0,01 0,04<br>0,17 0,63<br>0,33 0,21		
50	62,16 49,81<br>0,49 0,98<br>2,75 1,37	-0,01 0,06<br>0,00 0,02<br>0,08 0,11	0,00 0,01<br>0,00 0,02<br>0,01 0,01	0,00 0,01<br>0,00 0,02<br>0,01 0,01	0,00 0,01<br>0,01 0,05<br>0,01 0,01	0,00 0,01<br>0,11 0,26<br>0,04 0,05	-0,11 0,11<br>1,05 1,57<br>1,03 0,64	
100	53,02 105,67<br>0,26 0,51<br>3,30 1,20	0,00 0,04<br>0,01 0,05<br>0,08 0,05	0,00 0,01<br>0,01 0,06<br>0,03 0,03	-0,02 0,07<br>-0,01 0,09<br>0,09 0,23	0,00 0,01<br>0,06 0,14<br>0,06 0,06	0,00 0,01<br>0,33 0,59<br>0,18 0,18	-0,05 0,06<br>1,58 1,88<br>0,87 0,38	-0,37 0,22<br>-0,18 0,34<br>2,33 0,98

k=0,005	0,2	0,5	1	2	5	10	20	50
0,5	1,75 0,98<br>1418,39 1770,46<br>6,07 8,38							
1	3,37 0,97<br>683,59 890,93<br>3,20 5,16	0,12 0,35<br>823,84 1282,44<br>2,42 2,54						
2	7,45 1,87<br>184,92 288,40<br>5,17 6,42	0,19 0,39<br>78,99 225,88<br>0,56 0,34	-0,07 0,21<br>99,80 286,07<br>0,53 0,33					
5	18,28 5,27<br>74,60 110,94<br>3,53 3,79	0,00 0,20<br>0,01 0,03<br>0,47 0,36	0,01 0,05<br>0,01 0,04<br>0,04 0,03	-14,05 62,10<br>0,02 0,09<br>0,34 0,53				
10	33,18 13,53<br>41,46 45,72<br>3,22 3,16	-0,01 0,13<br>0,00 0,01<br>0,31 0,25	0,00 0,03<br>0,00 0,02<br>0,03 0,06	0,00 0,02<br>0,00 0,03<br>0,03 0,02	-0,01 0,06<br>0,27 1,10<br>0,35 0,25			
20	51,38 54,38<br>7,50 9,48<br>4,08 3,57	-0,02 0,11<br>0,00 0,02<br>0,19 0,17	-0,01 0,06<br>0,00 0,03<br>0,11 0,23	0,00 0,04<br>0,00 0,03<br>0,02 0,07	0,00 0,02<br>0,03 0,07<br>0,06 0,04	-0,07 0,10<br>2,39 4,31<br>0,74 0,53		
50	78,06 51,93<br>1,15 1,80<br>4,83 3,08	-0,03 0,13<br>-0,01 0,05<br>0,21 0,17	0,00 0,03<br>0,02 0,06<br>0,04 0,02	0,00 0,01<br>0,02 0,06<br>0,02 0,02	0,00 0,02<br>0,06 0,17<br>0,03 0,03	-0,01 0,04<br>1,35 1,73<br>0,36 0,26	-0,17 0,19<br>3,82 3,70<br>1,38 0,74	
100	67,47 76,06<br>0,79 1,00<br>4,58 2,37	-0,02 0,10<br>0,06 0,16<br>0,20 0,16	0,00 0,02<br>0,07 0,20<br>0,10 0,09	-0,01 0,07<br>0,11 0,40<br>0,18 0,23	0,00 0,01<br>0,60 0,95<br>0,27 0,26	-0,01 0,02<br>1,50 1,40<br>0,53 0,37	-0,16 0,13<br>1,47 1,46<br>1,40 0,55	-0,38 0,17<br>-0,13 0,40<br>3,00 1,12

k=0,01	0,2	0,5	1	2	5	10	20	50
0,5	-37,17 320,51<br>1999,38 2594,64<br>17,30 19,09							
1	3,41 1,30<br>521,56 715,99<br>4,78 7,71	-31,68 317,63<br>867,43 1024,66<br>3,00 4,11						
2	7,58 1,94<br>201,51 275,94<br>7,08 8,52	0,68 0,54<br>190,84 325,97<br>0,88 0,57	0,07 0,24<br>342,62 419,34<br>1,38 1,95					
5	-51,37 705,04<br>104,16 116,89<br>5,73 6,32	0,10 0,41<br>1,24 12,14<br>0,59 0,43	-3,30 32,68<br>0,01 0,08<br>0,14 0,38	-12,11 52,69<br>1,65 10,87<br>0,52 0,61				
10	37,66 11,95<br>36,10 46,90<br>5,75 6,08	0,04 0,32<br>0,01 0,04<br>0,47 0,32	0,00 0,07<br>0,00 0,04<br>0,08 0,16	0,00 0,06<br>0,02 0,07<br>0,12 0,18	-0,07 0,12<br>6,39 14,37<br>0,69 0,47			
20	66,61 26,31<br>8,20 8,79<br>5,97 4,89	0,02 0,22<br>0,00 0,04<br>0,34 0,30	0,00 0,06<br>0,00 0,04<br>0,10 0,18	-0,01 0,06<br>0,00 0,06<br>0,03 0,05	0,00 0,04<br>0,07 0,17<br>0,12 0,09	-0,16 0,15<br>9,06 10,49<br>1,15 0,65		
50	54,67 141,11<br>2,00 2,51<br>6,50 6,05	-0,71 6,73<br>0,01 0,10<br>0,44 0,90	0,00 0,05<br>0,02 0,11<br>0,06 0,05	0,00 0,03<br>0,05 0,17<br>0,04 0,05	-0,01 0,03<br>0,35 0,98<br>0,11 0,15	-0,03 0,07<br>2,88 2,77<br>0,72 0,42	-0,25 0,20<br>3,81 3,86<br>2,37 1,63	
100	65,48 105,31<br>0,87 1,16<br>6,86 4,41	-0,04 0,23<br>0,40 0,91<br>0,46 0,40	0,00 0,05<br>0,56 1,06<br>0,26 0,25	-0,01 0,06<br>0,88 1,66<br>0,38 0,39	0,00 0,02<br>2,01 2,18<br>0,58 0,48	-0,04 0,07<br>2,18 1,94<br>0,81 0,47	-0,25 0,18<br>1,90 1,72<br>1,91 0,94	-0,36 0,18<br>0,55 1,20<br>3,91 1,75

R=0.7

τ_2 × frame rate

k=0,02	0,2		0,5		1		2		5		10		20		50	
0,5	2320,54	23341,81														
	3191,48	12663,87														
	38,55	115,21														
1	4,21	2,16	0,43	0,42												
	860,44	1167,99	784,93	1035,01												
	8,05	13,37	5,01	7,47												
2	8,28	2,80	0,23	7,85	0,08	0,26										
	388,62	470,92	267,39	356,99	289,75	349,19										
	11,00	14,74	3,28	5,70	2,20	3,64										
5	-179,61	999,40	1,15	1,23	0,04	0,23	-5,35	30,53								
	94,76	120,97	30,98	63,85	3,25	17,31	28,17	76,60								
	8,09	10,83	0,76	2,13	0,26	0,18	0,64	0,58								
10	38,60	13,53	-0,54	17,76	0,02	0,18	-0,04	0,10	-0,14	0,19						
	28,77	36,28	6,57	16,45	0,04	0,09	0,03	0,15	27,51	38,85						
	7,64	7,63	0,66	0,77	0,11	0,10	0,21	0,25	1,03	0,80						
20	59,24	33,59	1,22	2,84	-0,01	0,11	0,00	0,09	-0,01	0,09	-0,15	0,20				
	8,18	9,40	0,99	3,41	0,03	0,08	0,04	0,12	2,69	5,64	17,54	14,13				
	10,93	12,00	0,65	0,60	0,10	0,11	0,07	0,17	0,33	0,25	1,56	1,23				
50	74,14	65,00	-1,49	20,48	0,00	0,11	-0,01	0,06	-0,01	0,06	-0,12	0,14	-0,22	0,21		
	2,79	2,98	0,62	1,57	0,35	1,05	0,32	0,94	2,63	4,21	5,51	4,33	5,00	3,97		
	11,10	9,51	0,83	2,01	0,17	0,16	0,13	0,16	0,44	0,49	1,37	0,48	3,58	2,46		
100	114,01	192,29	-1,22	13,06	-0,01	0,10	-0,01	0,07	-0,02	0,04	-0,11	0,11	-0,30	0,24	-0,43	0,18
	2,05	2,02	2,59	3,34	2,37	2,68	3,05	3,14	3,22	3,08	3,30	2,75	2,09	2,13	1,16	1,99
	8,64	8,08	0,79	0,77	0,61	0,45	0,71	0,54	0,75	0,56	1,29	0,48	3,18	2,40	5,51	2,59

τ_2 × frame rate

k=0,05	0,2		0,5		1		2		5		10		20		50	
0,5	-185521,3	763742,8														
	-34515,7	185406,6														
	480,6	1210,9														
1	-147348,3	602824,0	-28927,49	183317,82												
	-15057,4	65645,9	-9490,01	66431,96												
	311,1	929,3	142,02	752,10												
2	-1150,0	11189,5	-39,88	288,15	0,36	0,40										
	255,3	935,8	359,42	370,46	343,87	387,92										
	52,3	326,3	14,41	19,41	10,57	14,23										
5	-68,7	639,6	-17,31	218,05	-4,23	32,80	0,17	0,28								
	97,8	105,7	85,26	121,13	56,96	93,39	127,46	138,97								
	15,9	18,8	9,73	14,07	2,55	6,10	4,35	7,51								
10	43,1	20,7	6,67	4,99	-1,64	18,74	-1,05	10,38	-0,06	0,23						
	40,4	43,2	26,43	34,24	4,17	12,59	12,07	22,66	53,43	43,24						
	13,8	14,2	4,23	7,41	0,61	2,01	0,48	0,61	3,37	4,47						
20	66,6	46,4	7,37	6,87	0,11	0,41	0,01	0,18	-0,09	0,19	-0,11	0,19				
	12,5	12,2	8,12	11,60	1,77	5,51	2,47	7,22	16,16	18,50	20,29	19,27				
	22,0	19,8	5,52	10,44	0,33	0,33	0,21	0,28	1,02	0,54	1,50	1,03				
50	92,1	131,3	4,76	6,91	0,03	0,31	-0,01	0,12	-0,07	0,11	-0,17	0,17	-0,21	0,21		
	4,8	4,5	7,61	10,55	5,44	8,23	6,49	7,89	10,14	8,01	9,38	6,92	7,62	6,05		
	20,1	26,4	4,83	9,87	0,62	0,63	0,71	0,65	1,18	0,62	1,88	0,86	8,07	6,95		
100	130,4	185,9	11,57	61,64	-0,06	0,22	-0,04	0,12	-0,09	0,12	-0,20	0,18	-0,26	0,21	-0,53	0,19
	4,4	6,3	6,16	5,60	7,10	5,37	6,67	5,66	5,89	4,31	4,48	4,01	2,63	2,87	5,81	6,70
	24,1	32,6	2,82	4,86	0,92	0,66	1,02	0,72	1,17	0,63	2,06	2,03	5,94	5,29	8,74	6,30

R=0.8

τ_1 × frame rate

τ_2 × frame rate

k = 0,001	0,2	0,5	1	2	5	10	20	50
0,5	1,30 0,42<br>1300,14 2010,26<br>7,06 4,45							
1	3,06 0,70<br>902,16 1302,10<br>1,50 1,51	-0,06 0,29<br>311,32 1141,39<br>1,28 0,86						
2	5,02 2,04<br>400,18 616,62<br>1,00 0,81	-0,02 0,07<br>0,00 0,03<br>0,07 0,06	-0,02 0,05<br>0,02 0,08<br>0,30 0,24					
5	4,11 4,11<br>17,14 57,02<br>2,15 0,94	0,00 0,03<br>0,00 0,01<br>0,55 0,73	0,00 0,01<br>0,00 0,01<br>0,01 0,01	0,00 0,01<br>0,00 0,02<br>0,05 0,04				
10	4,74 7,34<br>2,57 12,03<br>2,56 1,10	0,00 0,02<br>0,00 0,00<br>0,03 0,02	0,00 0,03<br>0,00 0,02<br>0,03 0,30	0,00 0,00<br>0,00 0,01<br>0,01 0,01	-0,01 0,03<br>0,00 0,05<br>0,32 0,47			
20	7,74 14,32<br>0,19 1,23<br>2,75 1,29	0,00 0,02<br>0,00 0,01<br>0,03 0,02	0,00 0,00<br>0,00 0,01<br>0,00 0,00	0,00 0,00<br>0,00 0,01<br>0,00 0,00	0,00 0,00<br>0,00 0,02<br>0,02 0,01	-0,01 0,02<br>0,04 0,15<br>0,31 0,27		
50	-11,84 68,91<br>0,03 0,17<br>2,70 1,09	-0,01 0,04<br>0,00 0,02<br>0,04 0,08	0,00 0,00<br>0,00 0,02<br>0,01 0,01	0,00 0,00<br>0,00 0,02<br>0,01 0,00	0,00 0,00<br>0,00 0,04<br>0,01 0,01	0,00 0,01<br>0,04 0,13<br>0,03 0,03	-0,06 0,07<br>0,12 0,48<br>0,92 0,78	
100	6,96 22,78<br>0,06 0,23<br>2,87 0,87	0,00 0,04<br>0,00 0,05<br>0,05 0,04	0,00 0,00<br>0,01 0,04<br>0,02 0,02	0,00 0,00<br>0,02 0,05<br>0,03 0,02	0,00 0,00<br>0,04 0,11<br>0,05 0,05	0,00 0,01<br>0,36 0,57<br>0,21 0,21	-0,03 0,03<br>-0,05 0,46<br>0,37 0,31	-0,33 0,20<br>-0,19 0,33<br>2,93 1,26

τ_2 × frame rate

k=0,002	0,2	0,5	1	2	5	10	20	50
0,5	1,45 0,73<br>1376,22 2001,61<br>8,28 7,64							
1	3,26 0,79<br>719,18 1013,47<br>2,42 3,37	-0,04 0,29<br>446,95 1183,13<br>1,42 1,39						
2	6,63 1,90<br>443,82 579,53<br>1,46 2,02	-0,03 0,15<br>0,02 0,07<br>0,22 0,22	-0,06 0,12<br>13,87 136,27<br>0,58 0,43					
5	9,35 6,46<br>56,60 113,56<br>1,96 1,42	0,01 0,06<br>0,00 0,02<br>1,14 0,95	0,00 0,02<br>0,00 0,03<br>0,03 0,02	0,00 0,04<br>0,01 0,06<br>0,20 0,26				
10	16,96 12,19<br>19,25 33,77<br>2,87 2,29	0,00 0,05<br>0,00 0,01<br>0,17 0,33	-0,01 0,05<br>0,00 0,03<br>0,11 0,42	0,00 0,01<br>0,00 0,02<br>0,02 0,01	-0,01 0,02<br>0,02 0,08<br>0,28 0,33			
20	26,35 21,23<br>2,61 5,34<br>3,92 2,76	-0,01 0,04<br>0,00 0,01<br>0,06 0,04	0,00 0,01<br>0,00 0,01<br>0,01 0,01	0,00 0,01<br>0,00 0,02<br>0,01 0,00	0,00 0,01<br>0,00 0,04<br>0,03 0,03	-0,01 0,04<br>0,51 1,39<br>0,51 0,46		
50	26,62 69,78<br>0,68 1,14<br>4,03 1,86	-0,01 0,07<br>0,00 0,04<br>0,09 0,16	0,00 0,01<br>0,00 0,03<br>0,01 0,01	0,00 0,00<br>0,00 0,04<br>0,01 0,01	0,00 0,01<br>0,03 0,09<br>0,02 0,02	0,00 0,01<br>0,30 0,68<br>0,13 0,14	-0,13 0,13<br>0,88 1,53<br>1,50 1,15	
100	39,44 61,38<br>0,34 0,60<br>4,63 2,23	-0,02 0,08<br>0,00 0,10<br>0,12 0,10	0,00 0,01<br>0,02 0,10<br>0,06 0,05	0,00 0,00<br>0,02 0,13<br>0,06 0,06	0,00 0,00<br>0,18 0,48<br>0,14 0,16	0,00 0,01<br>0,95 1,14<br>0,44 0,38	-0,10 0,09<br>0,56 1,26<br>1,33 0,88	-0,36 0,19<br>-0,22 0,28<br>3,40 1,28

τ_2 × frame rate

k=0,005	0,2	0,5	1	2	5	10	20	50
0,5	-179,45 1066,13<br>1443,82 2197,11<br>11,37 14,13							
1	3,62 1,11<br>464,40 697,02<br>8,56 11,45	-0,01 0,35<br>638,75 1023,44<br>3,35 4,64						
2	7,33 1,89<br>294,71 447,22<br>4,49 7,04	0,04 0,33<br>19,46 134,55<br>0,63 0,29	-0,07 0,18<br>170,88 327,68<br>0,72 0,61					
5	12,91 5,90<br>57,39 83,52<br>3,37 3,83	-0,01 0,17<br>0,01 0,04<br>1,09 0,89	0,00 0,06<br>0,01 0,06<br>0,07 0,05	-0,03 0,07<br>0,04 0,14<br>0,54 0,49				
10	32,16 12,65<br>38,61 50,68<br>4,16 4,02	0,00 0,11<br>0,01 0,03<br>0,60 0,72	0,02 0,19<br>-0,02 0,07<br>0,18 0,56	-0,02 0,08<br>-0,01 0,06<br>0,29 0,69	-0,04 0,07<br>0,75 3,41<br>0,75 0,59			
20	53,78 24,95<br>9,03 9,94<br>7,20 6,56	-0,02 0,10<br>0,00 0,03<br>0,18 0,19	0,00 0,02<br>0,00 0,03<br>0,02 0,01	0,00 0,01<br>0,01 0,04<br>0,01 0,01	-0,01 0,04<br>0,02 0,10<br>0,15 0,31	-0,08 0,11<br>7,50 8,88<br>1,35 0,90		
50	51,06 306,19<br>1,95 2,24<br>7,35 4,89	0,00 0,11<br>0,02 0,08<br>0,18 0,14	0,00 0,04<br>0,02 0,09<br>0,05 0,06	0,00 0,01<br>0,03 0,11<br>0,03 0,03	0,00 0,04<br>0,15 0,39<br>0,09 0,14	-0,02 0,04<br>2,06 2,41<br>0,69 0,48	-0,22 0,19<br>3,78 4,16<br>2,38 1,14	
100	65,21 103,81<br>1,18 1,29<br>7,86 4,82	0,00 0,11<br>0,20 0,33<br>0,25 0,21	-0,01 0,06<br>0,36 0,84<br>0,25 0,31	0,00 0,01<br>0,48 0,91<br>0,26 0,30	0,00 0,01<br>1,26 1,58<br>0,52 0,49	-0,02 0,03<br>1,61 1,55<br>0,76 0,47	-0,21 0,16<br>1,53 1,79<br>2,34 1,14	-0,38 0,18<br>-0,14 0,37<br>4,84 1,71

τ_2 × frame rate

k=0,01	0,2	0,5	1	2	5	10	20	50
0,5	12,22 118,63<br>2221,36 2732,04<br>29,69 38,96							
1	3,99 2,15<br>700,83 742,70<br>11,35 17,28	0,18 0,33<br>636,30 943,59<br>3,78 5,33						
2	7,86 2,92<br>300,82 362,56<br>12,79 17,46	0,35 0,46<br>137,41 248,27<br>0,96 1,21	-0,02 0,22<br>323,14 437,65<br>1,59 1,96					
5	16,93 6,04<br>64,46 114,65<br>8,68 10,43	0,08 0,39<br>1,24 11,70<br>0,73 0,73	0,00 0,11<br>0,05 0,16<br>0,15 0,19	-2,61 25,30<br>4,05 21,75<br>0,67 0,66				
10	-12,01 475,12<br>37,13 39,85<br>7,38 6,13	-0,05 0,23<br>0,01 0,05<br>0,69 0,57	0,01 0,11<br>0,01 0,06<br>0,07 0,18	-1,04 10,08<br>0,01 0,12<br>0,64 1,01	-0,09 0,13<br>21,53 34,72<br>1,22 0,86			
20	54,53 29,57<br>7,82 9,31<br>9,88 9,44	0,20 1,39<br>0,00 0,06<br>0,53 0,51	0,00 0,06<br>0,01 0,06<br>0,04 0,03	0,00 0,03<br>0,02 0,08<br>0,03 0,02	-0,01 0,05<br>0,51 2,39<br>0,21 0,23	-0,19 0,17<br>11,76 12,66<br>2,18 1,28		
50	69,59 76,98<br>2,73 3,08<br>12,91 11,17	0,01 0,19<br>0,09 0,17<br>0,35 0,30	0,00 0,06<br>0,07 0,20<br>0,09 0,10	0,00 0,02<br>0,24 0,85<br>0,11 0,19	0,00 0,03<br>1,97 3,06<br>0,49 0,55	-0,07 0,07<br>4,02 3,69<br>1,42 0,56	-0,22 0,20<br>4,65 4,11<br>3,94 2,23	
100	149,98 259,84<br>1,58 1,71<br>10,84 8,88	-0,01 0,21<br>1,70 2,07<br>0,70 0,56	0,00 0,05<br>1,35 1,92<br>0,52 0,55	0,00 0,03<br>2,11 2,30<br>0,73 0,59	-0,01 0,02<br>3,14 2,68<br>1,01 0,72	-0,05 0,06<br>2,82 2,26<br>1,31 0,56	-0,25 0,17<br>2,17 2,07<br>3,49 1,88	-0,38 0,18<br>0,36 1,05<br>5,52 2,20

R=0.8

τ_2 × frame rate

k=0,02	0,2		0,5		1		2		5		10		20		50	
0,5	39,37	197,26														
	1513,64	2347,65														
	40,14	42,52														
1	6,27	5,16	0,18	1,84												
	509,70	695,84	760,64	943,20												
	29,07	37,69	7,21	9,86												
2	-18,91	270,05	-0,18	10,52	-0,04	1,09										
	283,70	366,89	216,10	305,93	320,65	381,02										
	22,23	27,31	4,35	7,54	3,97	5,95										
5	18,48	7,85	0,81	1,02	0,00	0,20	-10,63	46,25								
	99,50	116,40	26,24	61,64	5,01	23,24	57,84	97,73								
	13,82	17,98	0,98	2,67	0,36	0,33	0,98	0,92								
10	-192,58	2294,60	0,49	1,28	0,00	0,14	-0,04	0,12	-0,14	0,17						
	47,86	43,38	0,56	3,27	0,08	0,21	0,12	0,31	44,49	54,12						
	12,99	14,88	0,72	0,66	0,13	0,17	0,54	0,69	2,03	1,80						
20	57,71	30,26	0,70	2,08	0,01	0,11	-0,01	0,08	-0,04	0,08	-0,21	0,20				
	10,83	10,13	0,53	2,59	0,07	0,19	0,13	0,28	5,82	9,26	16,85	16,42				
	15,53	15,70	0,62	0,56	0,08	0,11	0,15	0,33	0,75	0,54	2,34	0,99				
50	67,50	177,46	-0,74	8,12	0,00	0,08	0,00	0,05	-0,03	0,06	-0,14	0,14	-0,22	0,19		
	3,23	3,90	0,83	1,77	1,61	2,93	2,10	3,41	5,46	4,82	6,73	5,44	4,94	4,72		
	15,95	17,07	0,92	2,10	0,37	0,45	0,46	0,54	1,03	0,66	2,14	0,76	5,99	4,31		
100	199,75	214,98	0,00	0,37	0,00	0,08	-0,01	0,04	-0,04	0,04	-0,12	0,13	-0,27	0,27	-0,41	0,19
	1,53	1,88	3,61	3,47	4,12	3,57	4,11	3,89	4,44	3,75	3,96	2,84	2,20	2,36	1,15	2,02
	15,67	15,22	1,04	0,84	1,06	0,75	1,06	0,87	1,26	0,84	2,14	0,95	5,01	3,88	7,70	3,22

τ_2 × frame rate

k=0,05	0,2		0,5		1		2		5		10		20		50	
0,5	-177514,0	762294,3														
	-37415,8	167967,7														
	456,8	1420,7														
1	-12520,2	575463,2	-79185,41	284836,15												
	4813,1	117048,5	-27617,04	101868,92												
	356,3	1412,0	491,46	1570,68												
2	-118144,0	539191,3	-22355,49	186496,49	-15,41	153,39										
	-7648,5	37529,2	-3580,38	32207,37	299,73	374,59										
	316,0	1167,7	283,53	1912,87	13,47	17,18										
5	-8205,4	81894,1	-6162,74	61086,99	0,21	0,52	-2,19	21,72								
	-161,6	2535,9	-378,77	4741,29	83,29	115,99	100,98	123,57								
	61,5	287,2	273,24	2612,88	2,21	4,42	4,71	7,87								
10	29,7	137,8	4,54	4,05	-1,35	14,42	-1,88	13,62	-0,05	0,23						
	33,1	33,7	34,16	45,06	10,83	25,09	23,52	32,92	49,51	54,15						
	23,2	26,8	4,49	8,74	0,69	1,82	0,96	2,00	5,83	8,04						
20	66,2	87,5	5,72	6,64	0,04	0,33	-0,02	0,15	-0,17	0,42	-0,13	0,22				
	14,2	14,2	12,20	15,15	5,79	11,02	10,53	17,85	23,07	21,54	19,51	18,73				
	27,5	34,3	8,08	18,21	0,58	0,64	0,63	0,70	2,31	2,87	3,49	3,58				
50	149,4	247,1	8,29	31,86	-0,04	0,23	-0,04	0,11	-0,11	0,13	-0,17	0,18	-0,26	0,21		
	6,5	6,9	10,26	9,51	8,53	9,77	9,48	9,73	12,32	8,78	9,93	7,30	5,53	5,33		
	36,9	45,2	2,98	5,89	1,15	0,89	1,20	0,99	2,09	1,47	2,73	1,28	10,95	8,49		
100	-5,3	2122,2	9,58	34,89	-0,03	0,37	-0,06	0,11	-0,11	0,13	-0,16	0,16	-0,22	0,19	-0,56	0,17
	3,7	6,0	8,14	6,22	8,05	6,50	7,68	6,04	7,66	5,66	4,67	3,87	2,57	2,68	5,19	5,50
	31,8	38,0	4,44	9,28	1,67	2,22	1,42	0,97	2,09	1,45	2,71	1,78	10,23	9,16	10,60	6,14

R=0.9

τ_1 × frame rate

τ_2 × frame rate

k = 0,001	0,2	0,5	1	2	5	10	20	50
0,5	-159,01 1070,59<br>5090,20 5372,22<br>17,36 69,59							
1	2,74 0,77<br>763,57 970,26<br>2,52 4,40	0,01 0,16<br>1168,15 1849,85<br>0,92 0,90						
2	3,44 1,87<br>105,89 239,73<br>1,99 1,50	0,00 0,07<br>0,01 0,06<br>5,85 2,50	-0,03 0,06<br>0,10 0,24<br>0,84 0,81					
5	2,23 2,35<br>3,17 16,12<br>3,89 1,88	0,02 0,17<br>-0,01 0,05<br>2,53 2,25	0,00 0,01<br>0,00 0,02<br>0,06 0,29	0,00 0,01<br>0,01 0,06<br>0,13 0,08				
10	2,73 4,68<br>2,34 11,41<br>3,50 1,94	0,00 0,02<br>0,00 0,01<br>0,03 0,02	0,00 0,01<br>0,00 0,01<br>0,01 0,01	0,00 0,00<br>0,01 0,02<br>0,02 0,01	-0,01 0,06<br>0,03 0,09<br>0,35 0,63			
20	2,69 6,91<br>0,21 1,42<br>3,74 1,82	0,00 0,02<br>0,00 0,01<br>0,03 0,02	0,00 0,00<br>0,00 0,01<br>0,00 0,00	0,00 0,00<br>0,00 0,02<br>0,01 0,00	-0,04 0,11<br>-0,02 0,08<br>1,03 1,71	-0,01 0,03<br>0,32 0,88<br>0,79 0,85		
50	1,33 22,64<br>0,13 0,58<br>4,46 2,01	0,00 0,02<br>0,00 0,03<br>0,04 0,03	0,00 0,00<br>0,01 0,03<br>0,04 0,29	0,00 0,00<br>0,00 0,04<br>0,01 0,01	0,00 0,00<br>0,03 0,09<br>0,02 0,02	0,00 0,01<br>0,31 0,66<br>0,34 0,77	-0,14 0,11<br>-0,35 0,12<br>3,41 2,17	
100	3,29 13,24<br>0,04 0,10<br>4,62 2,00	0,00 0,02<br>0,02 0,10<br>0,07 0,05	0,00 0,00<br>0,03 0,11<br>0,06 0,06	0,00 0,00<br>0,04 0,14<br>0,07 0,06	0,00 0,00<br>0,28 0,54<br>0,53 0,93	0,00 0,00<br>0,75 1,05<br>0,58 0,73	-0,08 0,07<br>-0,54 0,32<br>1,99 1,63	-0,38 0,21<br>-0,34 0,25<br>7,39 2,34

τ_2 × frame rate

k=0,002	0,2	0,5	1	2	5	10	20	50
0,5	-275,44 1094,54<br>2546,74 3260,58<br>18,23 56,10							
1	2,81 0,91<br>566,38 708,40<br>6,92 8,09	0,05 0,18<br>736,88 1316,02<br>1,37 1,63						
2	5,79 2,07<br>224,80 322,03<br>3,61 6,02	-0,02 0,16<br>0,05 0,15<br>5,85 2,61	-0,11 0,16<br>102,69 330,38<br>1,37 1,23					
5	7,94 5,21<br>22,81 48,80<br>5,59 4,66	-0,01 0,13<br>-0,01 0,05<br>3,18 2,39	0,00 0,02<br>0,01 0,05<br>0,17 0,59	0,00 0,02<br>0,03 0,10<br>0,23 0,23				
10	2,59 124,15<br>19,29 35,03<br>5,78 6,81	0,00 0,04<br>0,00 0,02<br>0,67 1,32	0,00 0,01<br>0,00 0,03<br>0,02 0,01	0,00 0,01<br>0,00 0,03<br>0,04 0,03	-0,03 0,08<br>0,07 0,25<br>0,88 0,97			
20	20,36 17,74<br>3,09 5,73<br>6,84 6,11	0,00 0,04<br>0,00 0,02<br>0,06 0,04	0,00 0,01<br>0,00 0,02<br>0,01 0,01	0,00 0,01<br>0,01 0,04<br>0,01 0,01	-0,03 0,10<br>0,01 0,12<br>0,91 1,43	-0,05 0,07<br>3,60 6,14<br>2,29 1,69		
50	24,54 45,86<br>0,86 1,71<br>8,82 6,06	0,00 0,03<br>0,00 0,06<br>0,06 0,05	-0,02 0,07<br>0,00 0,10<br>0,43 1,23	0,00 0,00<br>0,01 0,08<br>0,03 0,03	-0,01 0,05<br>0,08 0,19<br>0,13 0,49	-0,01 0,01<br>1,84 2,38<br>0,76 0,71	-0,17 0,15<br>0,49 1,34<br>3,45 2,36	
100	29,72 39,27<br>0,65 1,08<br>8,69 5,36	0,00 0,03<br>0,12 0,26<br>0,16 0,15	-0,01 0,06<br>0,18 0,55<br>0,44 0,94	0,00 0,00<br>0,28 0,67<br>0,22 0,30	0,00 0,00<br>1,21 1,55<br>0,88 0,92	0,00 0,01<br>2,05 1,62<br>1,17 0,91	-0,16 0,14<br>-0,15 0,82<br>3,10 2,01	-0,34 0,18<br>-0,29 0,27<br>7,05 2,66

τ_2 × frame rate

k=0,005	0,2	0,5	1	2	5	10	20	50
0,5	-68,09 807,42<br>2386,87 3260,73<br>42,30 54,42							
1	3,31 1,71<br>610,47 796,47<br>17,26 24,50	0,08 0,22<br>447,10 838,89<br>3,69 6,10						
2	7,03 2,59<br>249,09 313,96<br>15,89 24,72	-0,07 0,25<br>45,89 183,09<br>2,81 2,10	-0,06 0,17<br>468,40 549,23<br>1,62 2,58					
5	14,23 8,74<br>57,14 86,57<br>14,49 16,11	0,00 0,15<br>0,01 0,08<br>2,01 2,16	-0,04 0,11<br>-0,02 0,13<br>1,02 1,76	-0,03 0,07<br>4,91 26,22<br>0,81 0,79				
10	33,78 13,83<br>44,12 46,79<br>14,10 13,90	-0,03 0,12<br>0,00 0,05<br>1,07 1,43	-0,01 0,06<br>0,01 0,08<br>0,13 0,54	0,00 0,02<br>0,02 0,10<br>0,09 0,07	-0,10 0,12<br>13,48 28,37<br>2,38 1,78			
20	42,18 28,42<br>5,96 8,56<br>21,00 18,83	-0,02 0,10<br>0,00 0,06<br>0,69 1,24	-0,02 0,08<br>0,00 0,10<br>0,29 0,91	0,00 0,01<br>0,01 0,10<br>0,03 0,02	-0,01 0,05<br>0,16 0,33<br>0,33 0,60	-0,14 0,16<br>16,93 16,49<br>3,88 2,36		
50	42,14 207,78<br>3,52 3,51<br>18,52 12,34	0,09 0,93<br>0,09 0,21<br>0,35 0,79	-0,01 0,08<br>0,06 0,29<br>0,33 1,10	0,00 0,01<br>0,17 0,41<br>0,12 0,14	-0,01 0,06<br>1,98 3,15<br>0,68 0,87	-0,04 0,05<br>4,47 4,03<br>2,06 1,22	-0,25 0,18<br>3,24 3,92<br>6,58 3,44	
100	121,11 160,22<br>1,88 2,15<br>20,08 17,20	0,00 0,09<br>1,35 2,22<br>0,86 1,00	-0,04 0,10<br>1,66 2,31<br>1,25 1,40	0,00 0,01<br>2,21 2,58<br>1,00 0,93	-0,01 0,05<br>3,45 2,99<br>1,62 1,29	-0,04 0,05<br>2,78 2,42<br>2,14 1,08	-0,30 0,21<br>0,89 1,81<br>6,06 2,91	-0,39 0,18<br>-0,13 0,47<br>9,07 4,46

τ_2 × frame rate

k=0,01	0,2	0,5	1	2	5	10	20	50
0,5	-523,98 3390,13<br>1736,48 2881,55<br>92,21 112,83							
1	4,32 7,45<br>841,61 1109,55<br>38,13 50,57	0,14 0,26<br>588,43 998,63<br>6,39 8,99						
2	7,41 3,57<br>294,09 341,01<br>30,38 43,03	0,13 0,38<br>176,42 365,53<br>3,08 5,22	-0,05 0,20<br>463,41 463,63<br>4,08 7,17					
5	18,01 8,25<br>82,60 103,81<br>26,64 28,83	0,04 0,31<br>0,87 7,31<br>1,31 1,74	-0,06 0,13<br>1,07 9,15<br>1,14 1,59	-0,08 0,14<br>41,46 101,77<br>1,34 1,21				
10	38,99 14,35<br>33,69 37,71<br>20,13 17,78	-0,03 0,23<br>0,04 0,12<br>1,08 1,44	-0,01 0,08<br>0,01 0,13<br>0,22 0,63	-0,02 0,04<br>0,02 0,17<br>0,24 0,24	-0,13 0,14<br>41,97 52,50<br>3,54 2,50			
20	54,66 50,03<br>11,30 12,04<br>32,73 30,55	0,01 0,20<br>0,04 0,14<br>0,74 1,05	-0,01 0,07<br>0,03 0,15<br>0,20 0,85	0,00 0,03<br>0,13 0,23<br>0,06 0,05	-0,01 0,04<br>7,31 11,30<br>0,94 0,81	-0,17 0,16<br>24,36 23,52<br>4,38 2,34		
50	82,75 213,59<br>3,89 3,74<br>27,59 29,59	0,00 0,20<br>1,18 2,61<br>0,78 0,90	-0,01 0,06<br>1,04 2,37<br>0,51 0,92	0,00 0,02<br>1,51 3,08<br>0,45 0,70	-0,01 0,04<br>5,31 5,32<br>1,42 1,16	-0,10 0,13<br>8,23 6,31<br>3,55 1,61	-0,18 0,15<br>4,51 4,84<br>9,42 5,51	
100	246,28 303,67<br>2,33 2,53<br>32,82 37,91	-0,03 0,19<br>3,79 3,87<br>1,71 1,56	-0,02 0,08<br>4,46 4,20<br>1,92 1,57	-0,01 0,02<br>4,36 4,32<br>1,62 1,37	-0,02 0,04<br>4,64 4,03<br>1,82 1,24	-0,11 0,12<br>3,85 3,34<br>3,52 1,64	-0,21 0,17<br>2,23 2,64<br>8,15 4,71	-0,34 0,16<br>0,49 1,10<br>10,61 4,66

R=0.9

τ_2 × frame rate

k=0,02	0,2	0,5	1	2	5	10	20	50
0,5	74,95 228,74<br>1313,41 2510,90<br>75,84 88,46							
1	12833,17 127256,03<br>3902,20 32879,13<br>163,94 816,60	0,20 0,40<br>814,23 1100,61<br>11,44 19,15						
2	14,95 41,59<br>311,13 452,17<br>60,74 70,13	0,49 0,54<br>270,00 404,03<br>8,11 18,51	0,03 0,23<br>396,06 483,35<br>8,16 11,27					
5	-70,75 754,96<br>91,52 117,86<br>43,07 47,78	-28,78 288,53<br>27,74 63,84<br>1,37 1,47	-0,10 0,23<br>33,20 67,51<br>1,24 1,39	-0,12 0,26<br>109,81 149,88<br>2,48 4,38				
10	38,65 24,34<br>38,77 40,59<br>33,43 34,61	0,09 0,48<br>1,29 6,74<br>1,04 1,20	-0,03 0,15<br>2,06 8,31<br>0,38 0,91	-0,07 0,26<br>8,86 22,52<br>0,85 0,91	-0,11 0,17<br>52,63 69,15<br>5,84 6,52			
20	50,24 44,10<br>9,32 11,29<br>45,43 51,84	0,26 1,07<br>1,17 4,64<br>0,92 1,11	0,00 0,09<br>0,85 4,18<br>0,25 0,59	-0,01 0,06<br>3,51 7,95<br>0,49 0,66	-0,05 0,09<br>24,87 23,24<br>2,58 1,49	-0,15 0,17<br>21,33 22,22<br>5,19 3,13		
50	154,60 244,90<br>5,86 5,37<br>40,90 48,58	-0,01 0,33<br>5,68 7,63<br>1,77 1,75	-0,02 0,08<br>6,55 6,68<br>1,59 1,58	-1,91 18,35<br>6,26 7,91<br>1,49 1,60	-0,03 0,06<br>11,39 8,57<br>2,62 1,37	-0,14 0,15<br>9,82 8,16<br>4,79 2,88	-0,22 0,18<br>3,85 4,73<br>11,85 7,91	
100	1912,73 16528,05<br>7,38 43,57<br>77,86 236,24	0,04 0,99<br>8,19 6,77<br>3,68 12,00	-0,02 0,09<br>7,26 5,59<br>2,33 1,59	-0,02 0,04<br>6,21 5,67<br>2,03 1,58	-0,07 0,10<br>7,80 5,19<br>3,40 2,65	-0,19 0,16<br>4,42 3,95<br>4,76 2,38	-0,22 0,16<br>1,87 2,28<br>10,72 6,15	-0,44 0,20<br>1,62 2,18<br>13,79 6,22

τ_2 × frame rate

k=0,05	0,2	0,5	1	2	5	10	20	50
0,5	-141035,3 581905,3<br>-27245,1 167306,2<br>861,9 2328,3							
1	-181081,6 741456,4<br>-20415,0 89386,8<br>840,0 2614,8	-52044,49 256725,96<br>-15854,82 79345,14<br>571,87 2149,22						
2	-113143,4 576635,1<br>-5539,2 27981,3<br>557,9 1812,2	-14728,56 115114,11<br>-1887,77 17458,81<br>201,77 1078,86	-0,10 2,65<br>363,32 411,62<br>21,53 32,16					
5	-27116,2 283275,1<br>-613,4 7811,9<br>235,4 1116,6	-9,92 154,80<br>116,51 149,02<br>28,42 37,98	-15946,62 111976,29<br>-2200,13 16202,44<br>1183,97 8248,02	-5,71 56,08<br>131,04 133,42<br>16,46 22,73				
10	-15162,3 157627,3<br>-169,8 2383,5<br>146,2 793,6	3,32 4,05<br>44,46 55,15<br>15,37 28,28	-2333,14 23213,95<br>-133,76 1717,76<br>310,78 3074,11	-1,54 12,14<br>45,17 68,33<br>4,72 10,72	-0,10 0,23<br>57,66 65,46<br>12,48 15,07			
20	-32012,6 273828,3<br>-158,4 1473,2<br>252,7 1012,9	4,51 17,56<br>29,35 28,87<br>14,31 69,86	-2898,00 28834,84<br>-98,56 1178,19<br>543,70 5392,55	-0,06 0,16<br>28,17 28,75<br>2,73 5,41	-0,14 0,20<br>25,83 26,13<br>7,85 9,31	-0,11 0,18<br>21,66 18,78<br>9,17 10,85		
50	2360,7 15083,0<br>19,7 79,8<br>112,8 240,0	9,79 39,93<br>19,00 13,72<br>8,79 19,38	-1554,80 15469,66<br>-7,14 247,61<br>302,33 2978,83	-1,06 9,57<br>15,48 13,02<br>3,28 4,55	-0,12 0,14<br>15,75 12,80<br>6,46 7,85	-0,15 0,16<br>8,37 8,18<br>6,44 4,75	-0,24 0,17<br>4,93 5,53<br>17,58 13,50	
100	2688,6 23625,1<br>12,5 61,9<br>102,4 291,9	-85,31 1036,55<br>10,86 10,37<br>43,81 292,39	-0,09 0,21<br>11,12 8,22<br>4,37 10,58	-0,08 0,16<br>11,11 8,16<br>4,76 8,24	-0,13 0,18<br>6,29 5,86<br>8,86 10,74	-0,19 0,16<br>4,06 3,77<br>5,65 4,55	-0,25 0,19<br>2,47 2,90<br>19,45 23,56	-0,53 0,16<br>4,05 5,38<br>21,23 13,06
