# Supplementary material for: FRAP to Characterize Molecular Diffusion and Interaction in Various Membrane Environments
Source: PLoS One. 2016 Jul 7;11(7):e0158457. doi: 10.1371/journal.pone.0158457 (PMC4936743; doi:10.1371/journal.pone.0158457)
Supplement: S1 Text — (PDF) [file pone.0158457.s009.pdf]

## Experimental determination of the time t0 of the recovery phase

In order to take into account the contribution of fluorescence recovery that occurs during the photobleaching phase, the time t0 of the recovery phase in all FRAP and FRAPP experiments was set as the time of the last bleaching frame. We will show below that this approximation is valid using the FRAP experiment of Fig 2A as an example.

We will first present a protocol to experimentally determine the actual time t0 of the recovery phase. Then, we will show that our approximation (setting t0 as the time of the last bleaching frame) allows one to estimate the diffusion coefficient with a better than 10% accuracy.

In the following, we set t=0 as the time of the last bleaching frame, t=1 the time of the first post-bleach picture, t=2 the time of the second post-bleach picture, *etc*. The actual time t0 will be determined from the evolution of the post-bleach profile in the first five pictures of the recovery phase (S6 Fig).

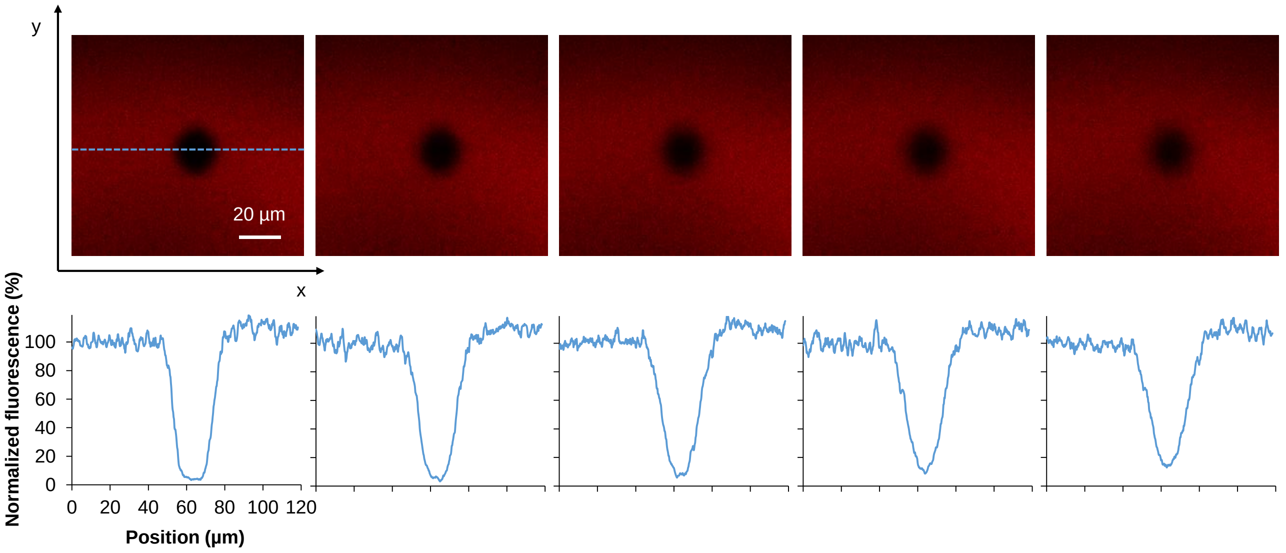


**S6 Figure.** First five post-bleach pictures and corresponding fluorescence profiles following photobleaching of a fluorescent disk of d = 20 µm diameter in the outer monolayer of a supported DOPC:DOPE-Rho (99:1) lipid bilayer (Fig 2A). The post-bleach profiles were obtained by measuring the fluorescence intensity (using ImageJ) along the blue dashed line as illustrated on the first post-bleach picture.

The local fluorescence recovery (at the edge of the bleaching pattern defining x=0) varies initially as:

$$F(x,t) = \frac{1}{2}\left(1 + \mathrm{erf}(\frac{x}{\sqrt{4Dt}})\right)$$

Where erf is the error function and:

$$F(x,0) = \begin{cases} 1 & for\ x > 0 \\ 1/2 & for\ x = 0 \\ 0 & for\ x < 0 \end{cases}$$

The slope S defining the post-bleach profile at x=0 (S7A Fig) is therefore proportional to $1/\sqrt{t}$, *i.e.* t varies as $1/S^2$. Plotting t as a function of $1/S^2$ should thus give a straight line, whose intercept with the y-axis is t0: $t = \alpha/S^2 + t0$. The five post-bleach profiles of S6 Fig were used to measure S at the different post-bleach times and to deduce the actual time t0 of the recovery phase. We found t0 = -0.4 sec (S7B Fig).

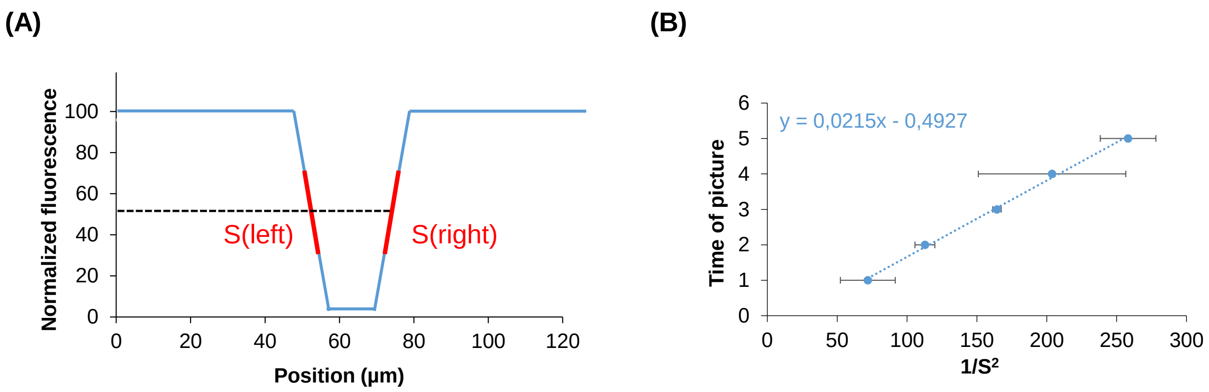


**S7 Figure.** (A) Schematic representation of the post-bleach profiles of S6 Fig and definition of the two local slopes that are used to characterize them. (B) The slopes on both sides, left and right, were measured and averaged for the five different time points. The linear fit directly provides the actual time t0 of the recovery phase as the intercept with the y-axis. Here, t0 = - 0.5 frame = - 0.4 sec (the scanning rate was 0.8 sec per frame in this FRAP experiment).

In theory, the protocol presented above to determine the actual time t0 of the recovery phase can be applied to any FRAP experiment. However, this renders the analysis quite complicated. Here, we will show that setting t0 = 0 allows one to obtain D with a good approximation and that it is therefore not necessary to systematically apply this protocol.

Let us show that this works on the same example (Fig 2A). Using the approximation t0 = 0, we find D = 2.1 $\mu m^2/s$ (S8A Fig). Using the protocol above to determine the actual time t0 (here t0 = - 0.5 frames = - 0.4 sec), we obtain D = 2.2 $\mu m^2/s$ (S8B Fig).

The difference between the two D values is therefore within the experimental error (1.9 ± 0.3 $\mu m^2/s$) over several experiments (Fig 3 and Table 2).

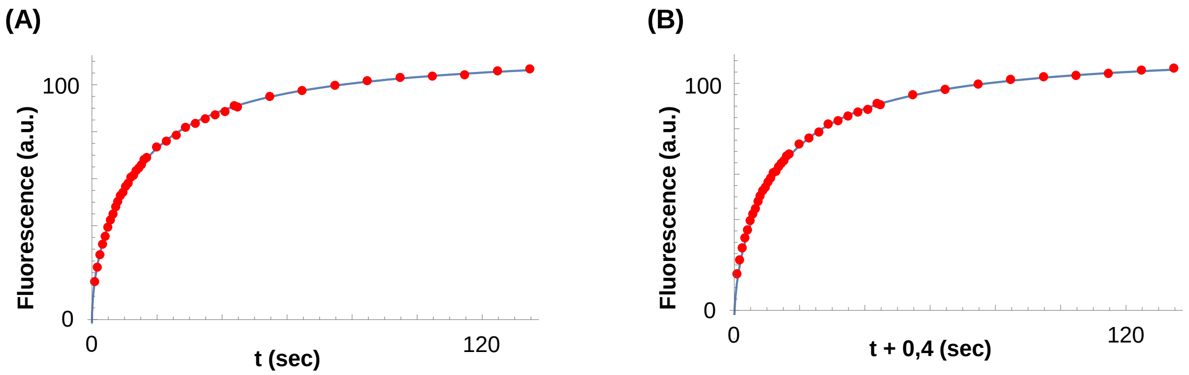


**S8 Figure.** Fitting of the experimental data from the FRAP experiment of Fig 2A using (A) t0 = 0 or (B) t0 = - 0.4 sec.

One should note that in most FRAP experiments, the error on t0 will usually be lower than the one measured in the present example. In fact, we have considered here an extreme case in which three bleaching frames were used, whereas a single bleaching frame is typically sufficient for bleaching. Using three bleaching frames obviously increases the error on t0. Since the error on D is negligible in this extreme case, our choice (setting t0 = 0 as the time of the last bleaching frame) will allow a good approximation of D in most cases.
